# One-Pot Regio- and Diastereoselective Gold-Catalyzed Propargylation of in Situ Activated Chromones and Consecutive Cyclization

**DOI:** 10.1021/acs.orglett.4c03812

**Published:** 2024-11-28

**Authors:** Julio Álvarez-Valle, Sergio Fernández, Cecilia Merino-Robledillo, Ignacio Funes-Ardoiz, Diego Sampedro, Javier Santamaría

**Affiliations:** † Departamento de Química Orgánica e Inorgánica e Instituto Universitario de Química Organometálica “Enrique Moles”, Unidad Asociada al C.S.I.C., Universidad de Oviedo, C/Julián Clavería 8, 33006 Oviedo, Spain; ‡ Departamento de Química, Instituto de Investigación en Química de la Universidad de La Rioja (IQUR), Universidad de La Rioja, c/Madre de Dios, 53, 26006 Logroño, Spain

## Abstract

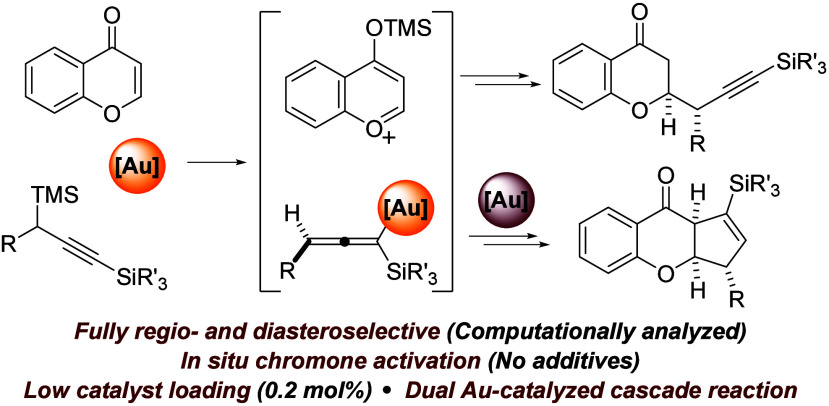

Herein, we report a gold-catalyzed propargylation of chromone derivatives by propargylsilanes. Chromones are synergistically activated by the silylium cation resulting from the gold activation of the propargylsilane. The reaction exclusively occurs at the C2-position of the chromone, and a single diastereoisomer is formed. Computational studies performed on the reaction mechanism rationalize the formation of the preferred diastereoisomer. Finally, a dual consecutive cascade reaction based on different gold catalysts enables the formation of complex tricyclic compounds.

Chromones (4*H*-chromen-4-ones), and their corresponding reduced analogues chromanones, can be considered as privileged structures as their skeleton is present in several compounds with biological activity.^1^ In this sense, this family of compounds and their derivatives have exhibited relevant pharmacological properties such as antitumoral,^2^ antiinflamatory,^3^ antiplatelet aggregation activity,^4^ or even, interesting effects against Alzheimer^5^ or Parkinson^6^ diseases, among others. Interestingly, chromanone derivatives bearing a substitution at position 2 have acquired higher relevance in this field. Therefore, several catalytic procedures involving alkylation^7^ or alkynylation^8^ at that position of the chromone skeleton have been reported. However, to our knowledge, no examples of catalytic propargylations of chromones have been described to date.

In this sense, we recently developed a new protocol for propargylation of carbonyl compounds^9^ through a gold(I)-catalyzed reaction with propargylsilanes without additive addition, as the carbonyl compound is synergistically activated by the silylium ion resulting from the gold-mediated activation of the propargylsilane. Taking advantage of this methodology, we opened the field to the synthesis of dihydrofurans,^10^ benzofulvenes^11^ or, in a process involving a double propargylation reaction, bis-propargylxanthene derivatives^12^ (Figure 1). Remarkably, among the scarce examples of catalytic propargylations starting from propargyl derivatives,^13^ gold-catalyzed propargylations are still restricted to our results, together with the works reported by Zhang.^14^ In addition, no examples of conjugated catalytic propargylation reactions of α,β-unsaturated carbonylic derivatives have been reported to date.

**Figure 1:**
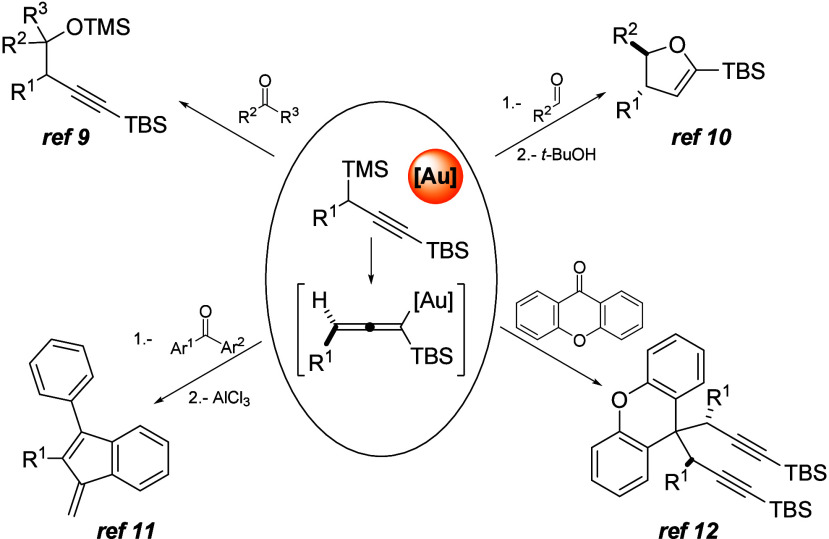
Propargylsilanes in gold-catalyzed propargylations.

With this information in hand and considering the interest of the chromanone skeleton, especially regarding those substituted at position 2, we envisioned chromone derivatives as attractive substrates to experiment gold-catalyzed propargylations. Thus, as they could be synergistically activated by the silylium cation as a benzopyrylium ion, they emerge as good candidates to accept a propargylic nucleophile at the conjugated position of the carbonyl functional group. Moreover, the proximity of the newly incorporated triple bond, in the enol ether intermediate, could allow for a consecutive cyclization to access tricyclic structures, in an overall formal [3 + 2] cycloaddition (Figure 2).

**Figure 2:**
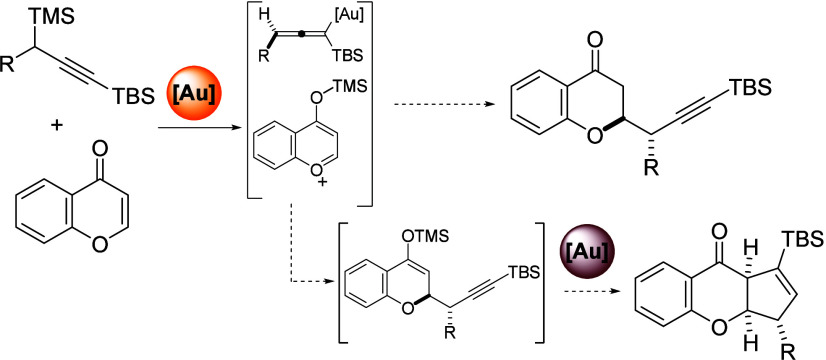
Working hypothesis.

Herein, we present a simple, high yielding, and fully regio- and diastereoselective gold-catalyzed propargylation of synergistically activated chromone derivatives, using propargylsilanes as starting material. In addition, consecutive gold-catalyzed propargylation and cyclization reactions were also achieved.

In the course of our investigations of propargylation of xanthone derivatives,^12^ we envisioned the use of chromone **1** as a potential electrophile for a related but different reactivity, due to the lack of one of the fused rings. In this sense, we initiated our study with chromone **1a** and propargylsilane **2a** as model compounds, and a phosphite ligand for the gold(I) catalyst (Scheme 1). After 2 h of reaction in methylene chloride, the reaction crude was analyzed and the formation of propargylated silyl enol ether **3a** was observed, together with a small amount of hydrolyzed chromanone **4a**. In both cases, propargyl derivatives were obtained as an almost equimolecular diastereoisomeric mixture. Due to the difficulties in isolating compound **3a** we decided to perform an *in situ* hydrolysis by adding methanol to the reaction vessel at the end of the experimental procedure. As a result of this, propargyl chromanone **4a** was obtained in a quantitative manner. The structures of compounds **3a** and **4a** were determined by NMR experiments and subsequently confirmed by an X-ray diffraction analysis performed on a 7-hydroxy substituted propargyl chromanone crystal (*vide infra*).

**Scheme 1:**
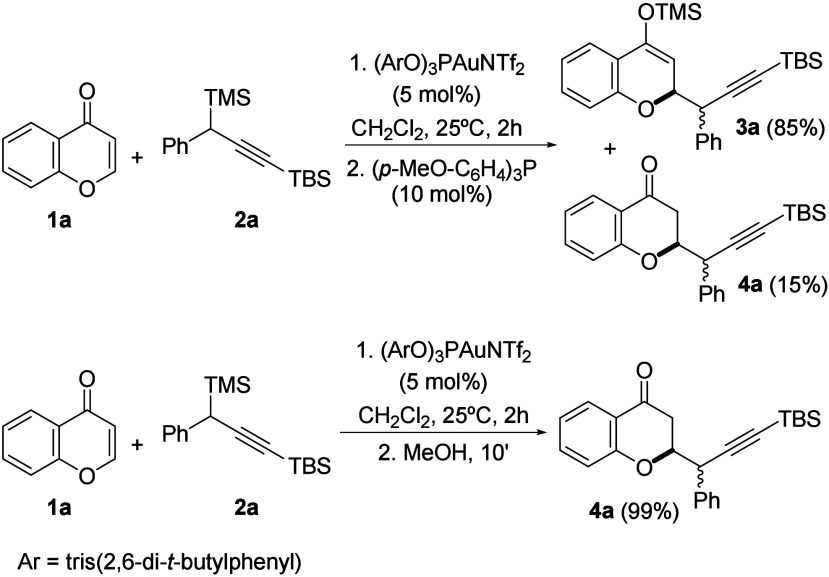
Preliminary Results Yields calculated by ^1^H-NMR, using dibromomethane as internal standard.

This reaction represents the first example of a conjugated catalytic nucleophilic propargylation of an α,β-unsaturated carbonyl compound, and on the contrary to other catalytic additions to carbonyls, it does not require the participation of any additive,^7,8,14^ as the carbonyl compound is synergistically activated by a silylium ion, exerted from the propargylsilane by the gold catalyst.^9^

At this point, due to the interest of the reaction, we decided to optimize the experimental conditions by modifying the ligand, reaction time, and catalyst loading (Table 1). In addition, the influence of several solvents in the reaction course was also studied. This study was performed at shorter reaction times and by lowering the catalyst loading in a quest to test the limits of the reaction (Table 1).

**Table 1:**
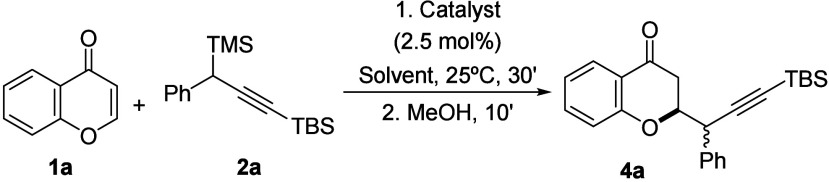
Catalyst and Solvent Screening

entry	catalyst	solvent	Yield^a^(%)	Conv.^a^(%)	dr
1	(ArO)_3_PAuNTf_2_	CH_2_Cl_2_	99	100	1:1
2	IPrAuNTf_2_	CH_2_Cl_2_	80	100	1:1
3	JohnPhosAuNTf_2_	CH_2_Cl_2_	50	60	1:1
4	PicAuCl_2_	CH_2_Cl_2_	–	–	–
5	Ph_3_PAuNTf_2_	CH_2_Cl_2_	90	93	1:1
6	AgNTf_2_	CH_2_Cl_2_	–	–	–
**7**	**(ArO)**_**3**_**PAuNTf**_**2**_	**Et**_**2**_**O**	**99**	**100**	**1:0**
**8**	**(ArO)**_**3**_**PAuNTf**_**2**_^b^	**Et**_**2**_**O**	**99**	**100**	**1:0**
**9**	**(ArO)**_**3**_**PAuNTf**_**2**_^c^	**Et**_**2**_**O**	**92**	**92**	**1:0**
10	(ArO)_3_PAuNTf_2_	THF	92	100	9:1
11	(ArO)_3_PAuNTf_2_	Dioxane	96	96	1:0
12	(ArO)_3_PAuNTf_2_	DCE	99	100	2:1
14	(ArO)_3_PAuNTf_2_	CH_3_Cl	99	100	5:1
14	(ArO)_3_PAuNTf_2_	CH_3_CN	80	80	3:1
**15**	**(ArO)**_**3**_**PAuNTf**_**2**_	**Toluene**	**99**	**100**	**1:0**
16	(ArO)_3_PAuNTf_2_	Hexane	88	88	1:0

a Yields and conversions calculated by ^1^H NMR, using dibromomethane as internal standard.

b Performed using a 1 mol % loading of the catalyst.

c 0.2 mol % catalyst loading and 1 h of reaction time.

From Table 1, it can be inferred that the phosphite ligand emerged as the best gold ligand for the catalytic propargylation, and to our delight, a modification in the solvent nature allowed for the formation of the propargylated compound **4a**, as a single diastereoisomer. Among the solvents tested, diethyl ether (entries 7–9) gave the best results, as the catalyst loading could be lowered to even 0.2 mol % (entry 9).^15^

As the next step, we focused our efforts on studying the scope of the reaction in terms of the substitution pattern of both chromone derivative **1** and propargylsilane **2**. For this study, although a lower catalyst loading could be employed, we selected 2.5 mol % for the standard reaction conditions in order to avoid pushing the reaction to its limits (Scheme 2).

**Scheme 2:**
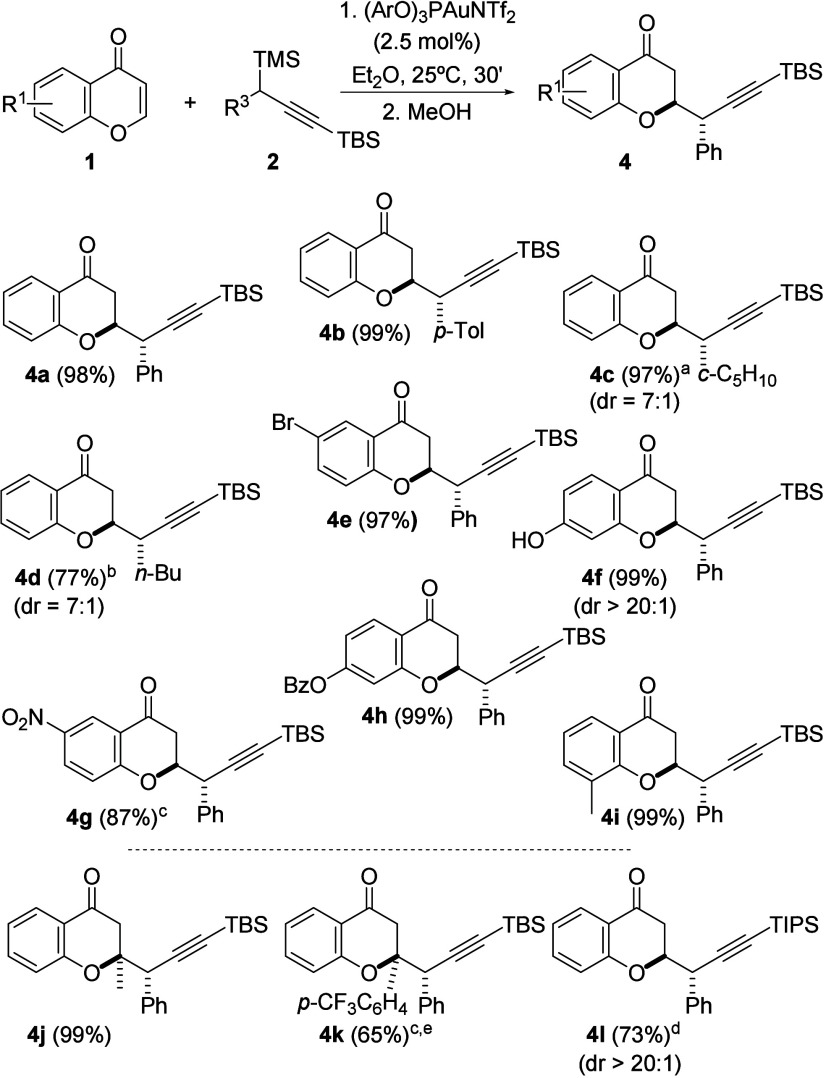
Scope of the Gold-Catalyzed Diastereoselective Synthesis of Propargyl Chromanones **4** 8 h of reaction. 5 h. 1 h. 3 h. Performed in refluxing Et_2_O.

The reaction works with high efficiency, almost quantitatively in most of the cases, and for the first time for gold-catalyzed propargylations of carbonyl derivatives, with full diastereoselectivity for a wide variety of substrates (Scheme 2). Thus, chromones bearing electron donating (for compounds **4f,h,i**) or electron withdrawing groups (for compounds **4e,g**) could be efficiently propargylated. Excitingly, 2-substituted chromones also gave positive results in their propargylation reaction (compounds **4j**,**k**). This reaction can be also extended to aliphatic and aromatic propargylsilanes **2**, although a slight decay of the diastereoselectivity was observed for the aliphatic ones (compounds **4c,d**). In order to establish the relative configuration of the major diastereoisomer—illustrated in compounds in Scheme 2—an X-ray diffraction analysis could be performed on compound **4f** (Figure 3).

**Figure 3:**
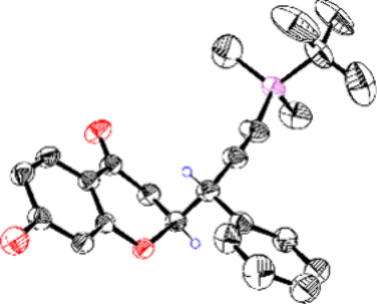
ORTEP view of propargyl chromanone **4f** with thermal ellipsoids at the 40% level.

Next, we turned our attention to the reaction mechanism and explored it via DFT calculations (see Supporting Information for the computational details). This mechanism is based on our previous results on the topic.^9−12^ First, both the initial propargylsilane and the chromone are activated through the nucleophilic attack of the chromone carbonyl to the electrophilic TMS unit on the alkyne, via an S_N_2 transition state **TS**_**I–II**_ (18.8 kcal/mol). In this step, an activated and highly electrophilic chromone and the corresponding gold-allene unit (**II**) are formed (Scheme 3A). Then, we explored the diastereoselectivity of the key determining step, which is defined by the nucleophilic attack of the σ-allenyl gold intermediate on the activated chromone at position 2 resulting in two possible transition states. Interestingly, **TS**_**II–III**_ is the most stable transition state (10.2 kcal/mol) but leads to an endergonic product formation, 3.7 kcal/mol higher than **II** (Scheme 3B) that then could release product **3a’** after coordination of a new alkyne molecule. However, these species are in equilibrium and can evolve toward the most stable adduct intermediate **IV**, which is 5.5 kcal/mol more stable than **II** and 2.1 kcal/mol more stable than **3a′**, through **TS**_**II–IV**_. This transition state has an affordable free energy barrier of 15.6 kcal/mol and leads to the most stable intermediate **IV**, which slightly shows a product inhibition effect but displaces the overall equilibrium toward the favored formation of diasteroselective product **3a**.

**Scheme 3:**
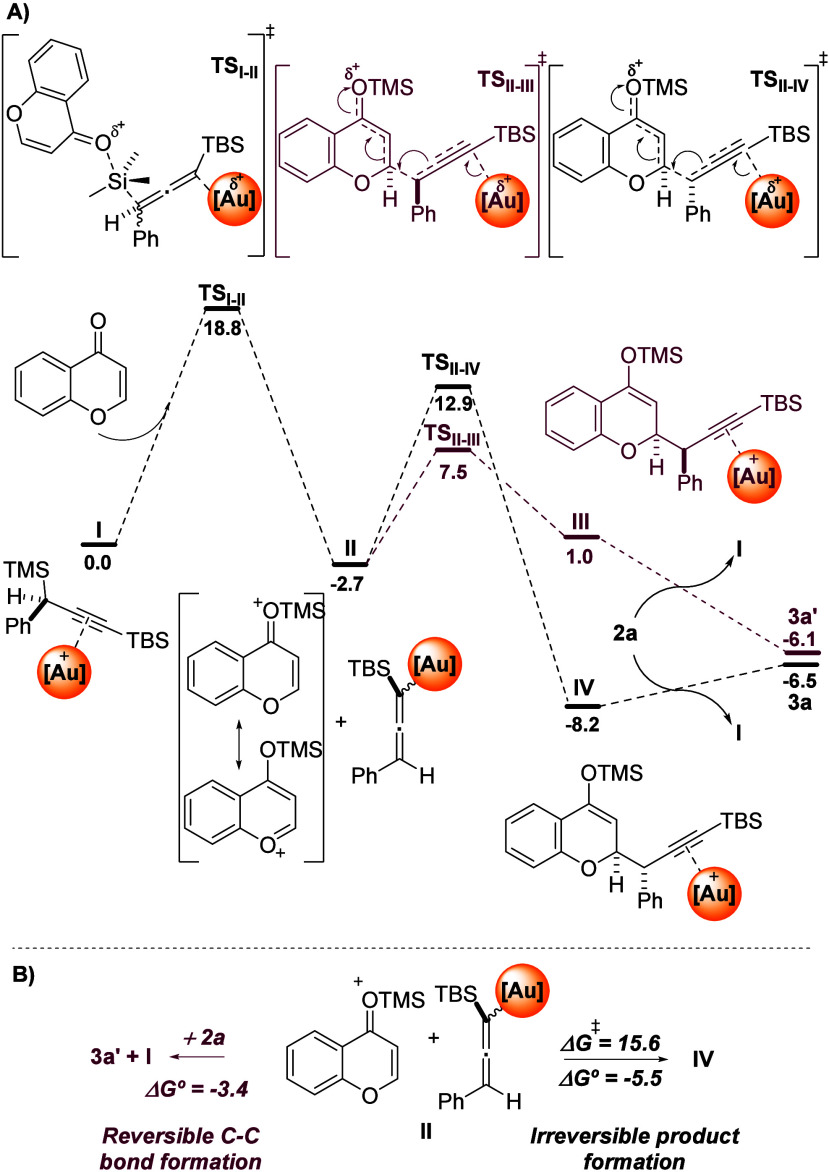
(A) Free Energy Profile of the Reaction Mechanism; (B) Thermodynamically Driven Formation of Intermediate IV All the energies in are expressed in kcal/mol.

The energy difference between intermediates **III** and **IV** comes from the steric hindrance of the phenyl group in **III**, which is very close to the chromone ring, compared to the longer distance in **IV** (see Figure S1 in the Supporting Information). Also, there are more effective noncovalent interactions between the gold center and the TMS moiety in **IV** than in **III.**

At this point, considering the nucleophilic nature of the silyl enol ether, which participates as a reaction intermediate, we envisioned the possibility of developing a more challenging transformation involving a consecutive gold-catalyzed cyclization. After several infructuous attempts with phosphite ligand, we observed that the addition of gold picolinate catalyst (PicAuCl_2_) after the propargylation step is completed resulted in the formation of cyclopentenbenzopyranone derivatives **5**, through a gold activation of the triple bond of enol ether **3** (Scheme 4).^16^ The one-pot procedure described here corresponds to a formal gold-catalyzed [3 + 2] cycloaddition of the propargylsilane **2** and the chromone derivative **1**, obtaining a single diastereoisomer and involving the formation of three new stereogenic centers. After the optimization stage, this transformation could also be achieved by starting from different chromones and propargylsilanes. Cyclopentenbenzopyranone compounds also represent interesting structures, as some derivatives exhibited antifungal properties.^17^

**Scheme 4:**
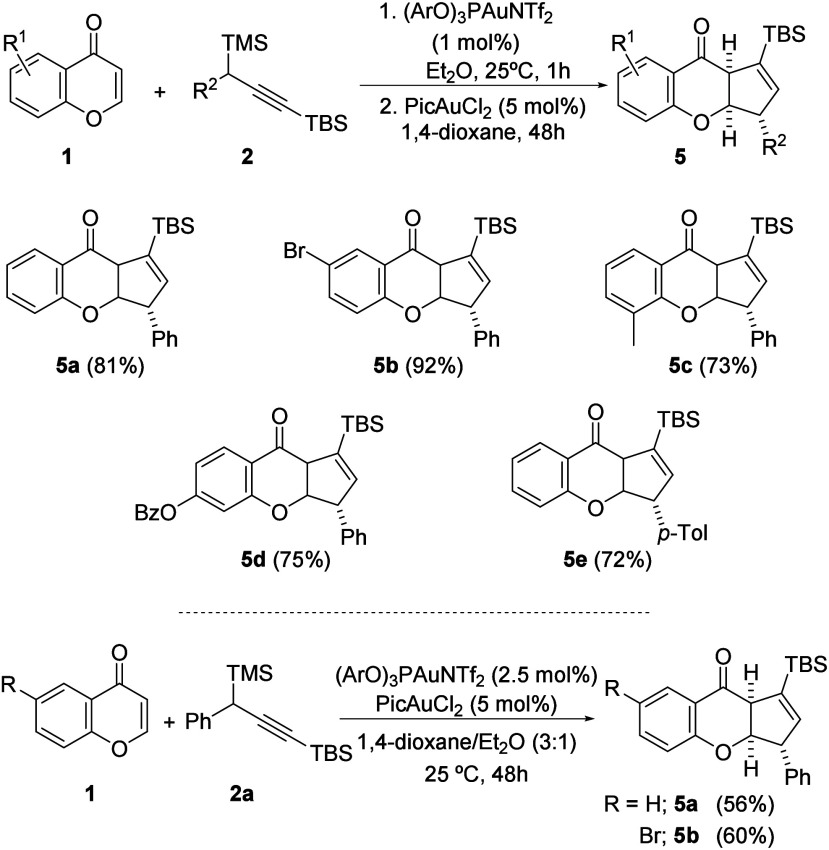
Gold-Catalyzed Consecutive Propargylation and Cyclization

Interestingly from a synthetic perspective, as it is described in Scheme 4*(bottom)*, consecutive gold-catalyzed propargylation and cyclization could also be performed with a simultaneous addition of both catalysts, affording the tricyclic compound **5a** with a slight decay in the reaction yield. This result indicates that the picolinate gold catalyst could remain under a kind of latency during the propargylation reaction accomplished by the phosphite gold catalyst.

In summary, in this work, we report a smooth gold-catalyzed propargylation of chromone derivatives using propargylsilanes as nucleophiles. The reaction proceeds in the absence of any additive because it is facilitated by the synergistic activation of the carbonylic group of the chromone by the silicon cation exerted during the propargysilane activation by the gold catalyst. In addition, it occurs with total regioselectivity at C2 carbon, facilitated by the formation of a benzopyrylium cation. Since that carbon can be considered as the conjugated position of the α,β-unsaturated carbonylic compound, this procedure represents the first example of a catalytic conjugated propargylation reaction of a carbonylic compound. On the other hand, the reaction takes place with total diastereoselectivity in most of the cases, a relevant novelty for gold-catalyzed propargylations. Moreover, a computational study performed on the reaction mechanism rationalizes the experimental results in terms of regio- and diastereoselectivity. The formation of the major diastereoisomer can be considered thermodynamically driven due to its higher stability and the reversibility observed for the key determining step. Finally, the major diastereoisomer could be transformed into an interesting tricyclic compound through the combination of two different gold catalysts. This reaction could be performed through a sequential addition of the catalysts or even as a dual consecutive cascade with a combination of both catalytic species.

## Data Availability

The data underlying this study are available in the published article and its Supporting Information.

## Supporting Information Available

Experimental procedures, NMR spectra, crystallographic data for compound **4f** and computational details (PDF)
FAIR data, including the primary NMR FID files, for compound **4** (ZIP)
FAIR data, including the primary NMR FID files, for compound **5** (ZIP)
