# Supplementary material for: One-Pot Regio- and Diastereoselective Gold-Catalyzed Propargylation of in Situ Activated Chromones and Consecutive Cyclization
Source: Org Lett. 2024 Nov 28;26(49):10487–92. doi: 10.1021/acs.orglett.4c03812 (PMC11650761; doi:10.1021/acs.orglett.4c03812)
Supplement: Supplementary file 1 — ol4c03812_si_001.pdf [file ol4c03812_si_001.pdf]

Supporting information for

One-pot Regio- and Diastereoselective Gold-Catalyzed Propargylation of In Situ Activated Chromones and Consecutive Cyclization

Julio Álvarez-Valle,† Sergio Fernández,† Cecilia Merino-Robledillo,‡ Ignacio Funes-Ardoiz,‡ Diego Sampedro‡,*, and Javier Santamaría†,*

†Departamento de Química Orgánica e Inorgánica and Instituto Universitario de Química Organometálica "Enrique Moles", Unidad Asociada al C.S.I.C. Universidad de Oviedo, C/ Julián Clavería, 8, 33006, Oviedo, Spain.

‡Departamento de Química, Instituto de Investigación en Química de la Universidad de La Rioja (IQUR), Universidad de La Rioja. c/ Madre de Dios, 53, 26006, Logroño, Spain

*Javier Santamaría jsv@uniovi.es

*Diego Sampedro diego.sampedro@unirioja.es

Table of Contents

1. Experimental methods....3

2. Experimental procedure for the diastereoselective synthesis of propargylchromanones **4**. 5

3. Experimental procedure for the one-pot synthesis of tricyclic compounds **5**. ..18

4. NMR Spectra....24

5. X-Ray diffraction analysis data....46

6. Computational Details....47

3D structures of adducts **III** and **IV** 48

Energies and Cartesian coordinates....49

1. Experimental methods.

All operations were carried out under argon atmosphere using conventional Schlenck techniques. unless otherwise noted. Diethyl ether and methanol were distilled from calcium hydride prior to its use. Hexane and ethyl acetate were used from commercial suppliers. TLC was performed on aluminium-backed plates coated with silica gel 60, with F254 indicator and flash chromatographic columns were carried out on silica gel (50-200 micron). NMR spectra were run on Bruker AV-300 and DPX-300 spectrometers using $CDCl_3$ as solvent. The X-ray analysis data has been collected at room temperature (298 K) using a Rigaku Oxford-Diffraction Xcalibur Nova diffractometer with microfocus source (Cu K_alpha radiation) and an Onyx CCD detector. High-resolution mass spectra (HRMS) were determined on a spectrometer Impact II (Bruker) with a ElectroSpray Ion source coupled to a quadrupole-*time of flight* mass analyzer (ESI-QTOF) and for compound **4c** HRMS were obtained in a GC-Q-TOF Agilent 7250 with Electron ionization (EI) source operating at 70 eV. Melting points were measured on a Büchi-Tottoli apparatus and were not corrected. Propargylsilanes **1** were synthesized following our previously published experimental procedure.[1] Commercially available 4*H*-chromen-4-one, 6-bromo-4*H*-chromen-4-one, 6-nitro-4*H*-chromen-4-one and 7-hydroxy-4*H*-chromen-4-one were used from their commercial sources without purification. 8-Methyl-4*H*-chromen-4-one,[2] 2-Methyl-4*H*-chromen-4-one[3] and 2-(4-(Trifluoromethyl)phenyl)-4*H*-chromen-4-one[3] were synthesized according to experimental literature and experimental data agree with those reported. 7-Benzyloxy-4*H*-chromen-4-one was synthesized according to the following procedure:

Experimental procedure for the synthesis of 7-benzoyloxychromen-4H-one:

Under argon atmosphere, to a solution at 0 ºC, of 486.3 mg (3 mmol) of 7-hydroxy-chromen-4H-one, 1 .26 mL (9 mmol) of triethylamine and 33 mg of DMAP (0.3 mmol) in 10 mL of dichloromethane, 523 µL (4.5 mmol; 1.5 equivalents.) of benzoyl chloride was added. The mixture was then allowed to stir at room temperature for 3 hours. After the reaction time, the solvents were removed under vacuum and a flash column chromatography through silica gel (Hexanes/ Ethyl acetate, (1:1)) afforded 695 mg (87 % yield) of the desired compound.

---

[1] Fernández, S.; González, J.; Santamaría, J.; Ballesteros, A. *Angew. Chem. Int. Ed.* **2019**, *58*, 10703-10707.
[2] Zhao, D.; Beiring, B.; Glorius, F. *Angew. Chem. Int. Ed.* **2013**, *52*, 8454-8458.
[3] Yan Zhou, Y.; Liang, H.; Sheng, Y.; Wang, S.; Gao, Y.; Zhan, L.; Zheng, Z.; Yang, M.; Liang, G.; Zhou, J.; Deng, J.; Song, Z. *J. Org. Chem.* **2020**, *85*, 9230-9243.

4-Oxo-4*H*-chromen-7-yl benzoate

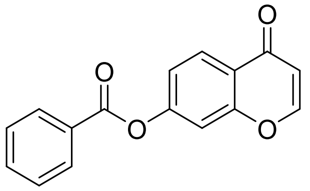

Yield: 87%, 695 mg

White solid: m.p.: 152.5-154.5 ºC

$R_f(SiO_2)$ = 0.44 (Hexanes/ Ethyl acetate, (1:1)).

^{1}H NMR (300 MHz, $CDCl_3$) δ 8.28 (d, *J* = 8.8 Hz, 1H), 8.22 (dt, *J* = 7.0, 1.4 Hz, 2H), 7.86 (d, *J* = 6.0 Hz, 1H), 7.74-7.64 (m, 1H), 7.54 (td, *J* = 7.2, 1.2 Hz, 2H), 7.42 (d, *J* = 2.1 Hz, 1H), 7.29 (dd, *J* = 8.7, 2.2 Hz, 1H), 6.36 (d, *J* = 6.0 Hz, 1H).

^{13}C NMR (75 MHz, $CDCl_3$) δ 176.9 (C), 164.4 (C), 157.0 (C), 155.5 (CH), 154.9 (C), 134.2 (CH), 130.3 (2 x CH), 128.8 (2 x CH), 128.7 (C), 127.4 (CH), 122.8 (C), 119.7 (CH), 113.2 (CH), 111.4 (CH),

HRMS (ESI) m/z $[M + H]^+$ Calcd. for $C_{16}H_{11}O_4$ 267.0652; Found 267.0650.

2. Experimental procedure for the diastereoselective synthesis of propargylchromanones 4.

In a schlenk under argon atmosphere, to 1 mL of a diethyl ether solution, at 25ºC, 0.2 mmol of the corresponding chromone **1**, 0.3 mmol (1.5 equivalents) of propargylsilane **2** (0.6 mmol; 3 equivalents for **4b-d,f,k** and 0.4 mmol; 2 equivalents for **4l**) and 5.6 mg of the gold catalyst (2.5 mol%) were added. The mixture was stirred at that temperature (refluxing Et_2O for **4k**) for the period described in Scheme 2 of the manuscript. At that point, 161 µL (128 mg; 4 mmol; 20 equivalents) of methyl alcohol was added and the mixture stirred for 10 minutes. Next, solvents were removed under vacuum and the residue purified under chromatographic column through silica-gel, using a mixture of hexanes and ethyl acetate as eluent. Following this procedure pure propargylchromanones **4** were obtained.

*For at 1 mmol scale of a representative compound **4a**:*

In a schlenk under argon atmosphere, to 5 mL of a diethyl ether solution, at 25ºC, 146 mg (1 mmol) of chromone, 453 mg (1.5 mmol) of the propargylsilane and 28 mg of the gold catalyst (2.5 mol%) were added. The mixture was stirred at 25ºC for 30 minutes and for an additional 10 minutes in the presence of 805 µL (640 mg; 20 mmol) of methyl alcohol. After solvents removal under vacuum and chromatographic purification of the residue through silica-gel (hexanes/ethyl acetate, (20:1)), 357 mg (95%) of propargylchromanone **4a** were obtained, as pure compound.

(R*)-2-[(R*)-3-(*tert*-butyldimethylsilyl)-1-phenylprop-2-yn-1-yl]chroman-4-one (4a)

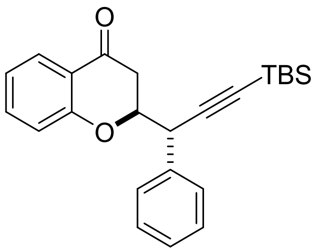

Yield: 98%, 74 mg

Colorless oil

$R_f(SiO_2)$ = 0.14 (Hexanes/ Ethyl acetate, (20:1)).

^{1}H NMR (300 MHz, $CDCl_3$) δ 7.89 (dd, *J* = 7.8 and 1.7 Hz, 1H), 7.60- 7.22 (m, 6H), 7.13-6.90 (m, 2H), 4.58 (dddd, *J* = 12.8, 5.7, 2.9 and 0.7 Hz, 1H), 4.34 (d, *J* = 5.6 Hz, 1H), 3.05 (dd, *J* = 16.9 and 12.8 Hz, 1H), 2.73 (ddd, *J* = 16.9, 2.8 and 0.7 Hz, 1H), 0.99 (s, 9H), 0.17 (s, 6H).

^{13}C NMR (75 MHz, $CDCl_3$) δ 192.6 (C), 161.6 (C), 136.7 (C), 136.5 (CH), 129.1 (2 x CH), 128.9 (2 x CH), 128.2 (CH), 127.3 (CH), 122.0 (CH), 121.4 (C), 118.4 (CH), 103.5 (C), 90.5 (C), 80.9 (CH), 44.3 (CH), 39.8 (CH_2), 26.6 (3 x CH_3), 17.0 (C), - 4.10 (CH_3), - 4.1F (CH_3).

HRMS (ESI) m/z $[M + H]^+$ Calcd. for $C_{24}H_{29}O_2Si$ 377.1931; Found 377.1946.

(R*)-2-[(R*)-3-(*tert*-butyldimethylsilyl)-1-(*p*-tolyl)prop-2-yn-1-yl]chroman-4-one (4b)

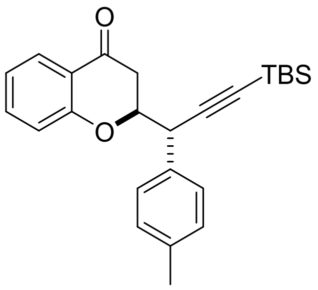

Yield: 99%, 77 mg

Colorless oil

R_f (SiO_2) = 0.30 (Hexanes/ Ethyl acetate, (20:1)).

^{1}H NMR (300 MHz, $CDCl_3$) δ 7.88 (dd, *J* = 7.8 and 1.7 Hz, 1H), 7.49 (ddd, *J* = 8.5, 7.2 and 1.8 Hz, 1H), 7.33 (d, *J* = 8.0 Hz, 2H), 7.19 (d, *J* = 7.8 Hz, 2H), 7.11-6.90 (m, 2H), 4.55 (ddd, *J* = 12.7, 5.7 and 2.9 Hz, 1H), 4.28 (d, *J* = 5.6 Hz, 1H), 3.03 (dd, *J* = 16.9 and 12.7 Hz, 1H), 2.72 (dd, *J* = 16.9 and 2.9 Hz, 1H), 2.37 (s, 3H), 0.98 (s, 9H), 0.16 (s, 3H), 0.15 (s, 3H).

^{13}C NMR (75 MHz, $CDCl_3$) δ 192.3 (C), 161.2 (C), 137.5 (C), 136.0 (CH), 133.1 (C), 129.3 (2 x CH), 128.3 (2 x CH), 126.9 (CH), 121.5 (CH), 121.0 (C), 118.0 (CH), 103.3 (C), 89.8 (C), 80.5 (CH), 43.5 (CH), 39.3 (CH_2), 26.1 (3 x CH_3), 21.1 (CH_3), 16.6 (C), - 4.5 (CH_3), - 4.6 (CH_3).

HRMS (ESI) m/z $[M + H]^+$ Calcd. for $C_{25}H_{31}O_2Si$ 391.2088; Found 391.2095.

(R*)-2-[(R*)-3-(*tert*-butyldimethylsilyl)-1-cyclopentylprop-2-yn-1-yl]chroman-4-one (4c)

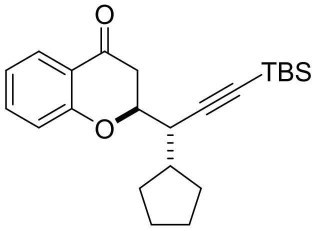

Yield: 97%, 71 mg; (7:1 dr)

Colorless oil

R_f (SiO_2) = 0.38 (Hexanes/ Ethyl acetate, (20:1)).

^{1}H NMR (300 MHz, $CDCl_3$) *(Major isomer)* 7.90 (dd, J = 7.8 and 1.8 Hz, 1H), 7.49 (ddd, J = 8.6, 7.2 and 1.8 Hz, 1H), 7.14-6.91 (m, 2H), 4.35 (ddd, J = 9.9, 7.2 and 5.6 Hz, 1H), 3.02-2.82 (m, 3H), 1.92-1.24 (m, 9H), 0.94 (s, 9H), 0.11 (s, 6H).

^{13}C NMR (75 MHz, $CDCl_3$) *(Major isomer)* δ 192.4 (C), 161.2 (C), 136.0 (CH), 126.9 (CH), 121.5 (C), 121.1 (CH), 117.9 (CH), 105.2 (C), 87.7 (C), 79.1 (CH), 40.8 (2 x CH_2), 38.1 (CH), 30.6 (CH_2), 29.1 (CH_2), 26.1(3 x CH_3), 22.4 (CH_2), 16.5 (C), 14.0 (CH), - 4.5 (2 x CH_3).

HRMS (EI) m/z [M - C_4H_9] Calcd. for $C_{19}H_{23}O_2Si$ 311.1467; Found 311.1470.

(R*)-2-[(R*)-1-(*tert*-butyldimethylsilyl)hept-1-yn-3-yl]chroman-4-one (4d)

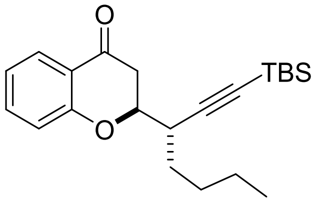

Yield: 77%, 55 mg

Colorless oil

R_f (SiO_2) = 0.27 (Hexanes/ Ethyl acetate, (20:1)).

^{1}H NMR (300 MHz, $CDCl_3$) *(Major isomer)* δ 7.90 (dd, *J* = 7.8, 1.8 Hz, 1H), 7.49 (ddd, *J* = 8.5, 7.2 and 1.7 Hz, 1H), 7.12-6.96 (m, 2H), 4.35 (ddd, *J* = 9.9, 7.2 and 5.6 Hz, 1H), 3.00-2.84 (m, 3H), 1.90-1.22 (m, 7H), 0.95 (t, *J* = 6.4 Hz, 3H), 0.94 (s, 9H), 0.11 (s, 6H).

^{13}C NMR (75 MHz, $CDCl_3$) *(Major isomer)* δ 192.4 (C), 161.2 (C), 136.0 (CH), 126.9 (CH), 121.5 (CH), 121.1 (C), 117.9 (CH), 105.2 (C), 87.7 (C), 79.0 (CH), 40.8 (CH_2), 38.1 (CH), 30.6 (CH_2), 29.1 (CH_2), 26.1 (3 x CH_3), 22.4 (CH_2), 16.5 (C), 14.0 (CH_3), - 4.5(CH_3), - 4.5 (CH_3).

HRMS (ESI) m/z $[M + H]^+$ Calcd for $C_{22}H_{33}O_2Si$ 357.2244; Found 357.2243.

(R*)-6-bromo-2-[(R*)-3-(*tert*-butyldimethylsilyl)-1-phenylprop-2-yn-1-yl]chroman-4-one (4e)

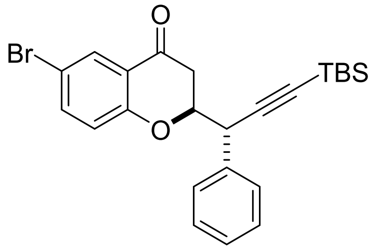

Yield: 97%, 80 mg

White solid: m.p.: 100-102ºC

R_f (SiO_2) = 0.35 (Hexanes/ Ethyl acetate, (20:1)).

^{1}H NMR (300 MHz, $CDCl_3$) δ 7.98 (d, *J* = 2.5 Hz, 1H), 7.56 (dd, *J* = 8.8 and 2.5 Hz, 1H), 7.50-7.23 (m, 5H), 6.91 (d, *J* = 8.8 Hz, 1H), 4.56 (ddd, *J* = 12.8, 5.7 and 2.9 Hz, 1H), 4.33 (d, *J* = 5.6 Hz, 1H), 3.03 (dd, *J* = 17.0 and 12.8 Hz, 1H), 2.72 (dd, *J* = 17.0 and 2.9 Hz, 1H), 0.97 (s, 9H), 0.16 (s, 3H), 0.16 (s, 3H).

^{13}C NMR (75 MHz, $CDCl_3$) δ 191.4 (C), 160.4 (C), 139.1 (CH), 136.3 (C), 129.7 (CH), 129.2 (2 x CH), 128.9 (2 x CH), 128.3 (CH), 122.6 (C), 120.5 (CH), 114.6 (C), 103.1 (C), 90.8 (C), 81.0 (CH), 44.2 (CH), 39.2 (CH_2), 26.5 (3 x CH_3), 17.0 (C), - 4.1 (2 x CH_3).

HRMS (ESI) m/z $[M + H]^+$ Calcd. for $C_{24}H_{28}BrO_2Si$ 455.1036; Found 455.1040.

(R*)-2-[(R*)-3-(*tert*-butyldimethylsilyl)-1-phenylprop-2-yn-1-yl]-7-hydroxychroman-4-one (4f)

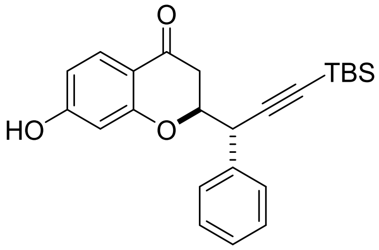

Yield: 99%, 78 mg

White solid: m.p.: 95-97ºC

R_f (SiO_2) = 0.17 (Hexanes/ Ethyl acetate, (5:1)).

^{1}H NMR (300 MHz, $CDCl_3$) δ 7.90 (bs, 1H), 7.79 (d, J = 8.6 Hz, 1H), 7.56-7.21 (m, 5H), 6.58 (dd, J = 8.7 and 2.3 Hz, 1H), 6.45 (d, J = 2.3 Hz, 1H), 4.65-4.46 (m, 1H), 4.30 (d, J = 5.7 Hz, 1H), 3.03 (dd, J = 17.0, 12.6 Hz, 1H), 2.68 (dd, J = 17.0 and 3.0 Hz, 1H), 0.97 (s, 9H), 0.16 (s, 3H), 0.15 (s, 3H).

^{13}C NMR (75 MHz, $CDCl_3$) δ 192.7 (C), 164.5 (C), 164.0 (C), 136.5 (C), 129.7 (CH), 129.1 (2 x CH), 128.9 (2 x CH), 128.2 (CH), 114.8 (C), 111.4 (CH), 103.8 (C), 103.4 (CH), 90.5 (C), 81.0 (CH), 44.2 (CH), 39.2 (CH_2), 26.5 (3 x CH_3), 17.0 (C), - 4.1 (2 x CH_3).

HRMS (ESI) m/z $[M + H]^+$ Calcd. for $C_{24}H_{29}O_3Si$ 393.1880; Found 393.1882.

(R*)-2-[(R*)-3-(*tert*-butyldimethylsilyl)-1-phenylprop-2-yn-1-yl]-6-nitrochroman-4-one (4g)

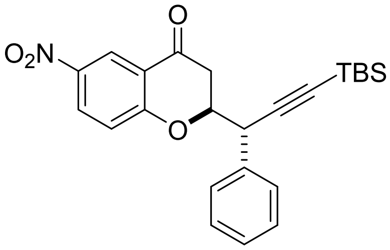

Yield: 87%, 73 mg.

White solid: m.p.: 144-146ºC

R_f (SiO_2) = 0.57 (Hexanes/ Ethyl acetate, (5:1)).

^{1}H NMR (300 MHz, $CDCl_3$) δ 8.77 (d, J = 2.8 Hz, 1H), 8.35 (dd, J = 9.1 and 2.9 Hz, 1H), 7.52-7.30 (m, 5H), 7.13 (d, J = 9.0 Hz, 1H), 4.70 (ddd, J = 12.5, 5.6 and 3.1 Hz, 1H), 4.38 (d, J = 5.6 Hz, 1H), 3.08 (dd, J = 17.0 and 12.5 Hz, 1H), 2.82 (dd, J = 17.0 and 3.1 Hz, 1H), 0.97 (s, 9H), 0.16 (s, 3H), 0.16 (s, 3H).

^{13}C NMR (75 MHz, $CDCl_3$) δ 190.3 (C), 165.4 (C), 142.6 (C), 135.8 (C), 130.8 (CH), 129.3 (2 x CH), 128.8 (2 x CH), 128.5 (CH), 123.9 (CH), 120.9 (C), 119.7 (CH), 102.6 (C), 91.3 (C), 81.6 (CH), 44.1 (CH), 39.0 (CH_2), 26.5 (3 x CH_3), 16.0 (C), - 4.2 (2 x CH_3).

HRMS (ESI) m/z $[M + H]^+$ Calcd. for $C_{24}H_{28}NO_4Si$ 422.1782; Found 422.1801.

(R*)-2-[(R*)-3-(*tert*-butyldimethylsilyl)-1-phenylprop-2-yn-1-yl]-4-oxochroman-7-yl benzoate (4h)

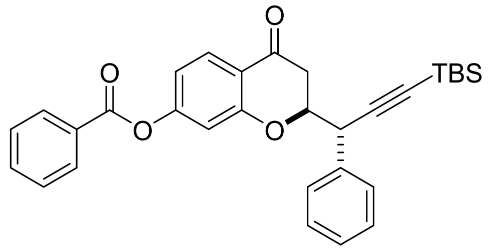

Yield: 99%, 98 mg

White solid: m.p.: 131-133ºC

R_f (SiO_2) = 0.26 (Hexanes/ Ethyl acetate, (10:1)).

^{1}H NMR (300 MHz, $CDCl_3$) δ 8.29-8.15 (m, 2H), 7.97 (d, *J* = 9.2 Hz, 1H), 7.74-7.60 (m, 1H), 7.61-7.23 (m, 7H), 6.93 (dq, *J* = 3.4 and 2.2 Hz, 2H), 4.63 (ddd, *J* = 12.8, 5.8 and 2.9 Hz, 1H), 4.33 (d, *J* = 5.9 Hz, 1H), 3.06 (dd, *J* = 16.9 and 12.8 Hz, 1H), 2.78 (dd, *J* = 16.9 and 2.9 Hz, 1H), 1.01 (s, 9H), 0.19 (d, *J* = 2.1 Hz, 6H).

^{13}C NMR (75 MHz, $CDCl_3$) δ 191.4 (C), 164.7 (C), 162.6 (C), 157.3 (C), 136.5 (C), 134.4 (CH), 130.7 (2 x CH), 129.3 (C), 129.1 (4 x CH), 128.9 (2 x CH), 128.8 (CH), 128.2 (CH), 119.4 (C), 116.2 (CH), 111.6 (CH), 103.4 (C), 90.5 (C), 81.4 (CH), 44.3 (CH), 39.7 (CH_2), 26.6 (3 x CH_3), 17.0 (C), - 4.1 (2 x CH_3).

HRMS (ESI) m/z $[M + H]^+$ Calcd. for $C_{31}H_{33}O_4Si$ 497.2143; Found 497.2145

(R*)-2-[(R*)-3-(*tert*-butyldimethylsilyl)-1-phenylprop-2-yn-1-yl]-8-methylchroman-4-one (4i)

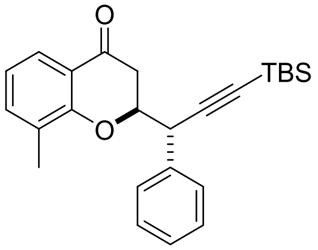

Yield: 99%, 77 mg

Colorless oil

R_f (SiO_2) = 0.25 (Hexanes/ Ethyl acetate, (20:1)).

^{1}H NMR (300 MHz, $CDCl_3$) δ 7.75 (dd, *J* = 7.9 and 1.7 Hz, 1H), 7.58-7.21 (m, 6H), 6.92 (t, *J* = 7.5 Hz, 1H), 4.56 (ddd, *J* = 11.3, 6.9 and 4.0 Hz, 1H), 4.25 (d, *J* = 6.9 Hz, 1H), 3.06-2.81 (m, 2H), 2.13 (s, 3H), 1.00 (s, 9H), 0.18 (s, 6H).

^{13}C NMR (75 MHz, $CDCl_3$) δ 192.4 (C), 159.4 (C), 136.9 (CH), 136.9 (C), 128.8 (2 x CH), 128.6 (2 x CH), 127.8 (CH), 127.4 (C), 124.5 (CH), 121.0 (CH), 120.8 (C), 103.6 (C), 89.4 (C), 80.5 (CH), 44.1 (CH), 40.4 (CH_2), 26.2 (3 x CH_3), 16.7 (C), 15.7 (CH_3), - 4.4 (2 x CH_3).

HRMS (ESI) m/z $[M + H]^+$ Calcd. for $C_{25}H_{31}O_2Si$ 391.2088; Found: 391.2098.

(R*)-2-[(R*)-3-(*tert*-butyldimethylsilyl)-1-phenylprop-2-yn-1-yl]-2-methylchroman-4-one (4j)

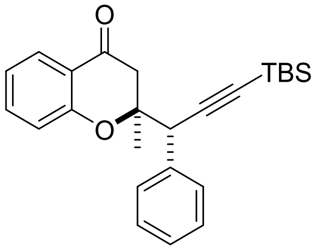

Yield: 99%, 77 mg

Colorless oil

R_f (SiO_2) = 0.13 (Hexanes/ Ethyl acetate, (20:1)).

^{1}H NMR (300 MHz, $CDCl_3$) δ 7.91 (dd, *J* = 7.8 and 1.8 Hz, 1H), 7.65-7.42 (m, 1H), 7.36 (m, 5H), 7.03 (t, *J* = 7.6 Hz, 1H), 6.93 (d, *J* = 8.3 Hz, 1H), 4.21 (s, 1H), 3.27 (d, *J* = 16.6 Hz, 1H), 2.85 (d, *J* = 16.6 Hz, 1H), 1.40 (s, 3H), 1.00 (s, 9H), 0.16 (s, 3H), 0.16 (s, 3H).

^{13}C NMR (75 MHz, $CDCl_3$) δ 192.2 (C), 159.7 (C), 136.8 (CH), 136.3 (C), 130.3 (2 x CH), 128.4 (2 x CH), 128.0 (CH), 126.9 (CH), 121.5 (CH), 120.8 (C), 118.9 (CH), 105.0 (C), 89.2 (C), 82.8 (C), 47.3 (CH), 46.7 (CH_2), 26.6 (3 x CH_3), 21.5 (CH_3), 17.2 (C), - 4.2 (2 x CH_3).

HRMS (ESI) m/z $[M + H]^+$ Calcd. for $C_{25}H_{31}O_2Si$ 391.2088; Found 391.2099.

(R*)-2-[(R*)-3-(*tert*-butyldimethylsilyl)-1-phenylprop-2-yn-1-yl]-2-(*p*-trifluoromethylphenyl)methylchroman-4-one (4k)

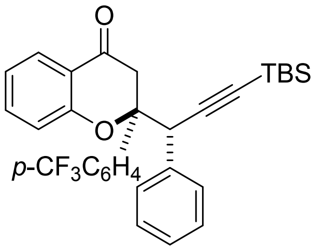

Yield: 65%, 68 mg

Yellowish oil

R_f (SiO_2) = 0.39 (Hexanes/ Ethyl acetate, (10:1)).

^1H NMR (300 MHz, $CDCl_3$) δ 7.73 (dd, J = 7.8 and 1.8 Hz, 1H), 7.50 (d, J = 1.4 Hz, 1H), 7.40 (d, J = 8.2 Hz, 2H), 7.34-6.86 (m, 9H), 4.42 (s, 1H), 3.56 (d, J = 1.7 Hz, 2H), 0.98 (s, 9H), 0.17 (s, 3H), 0.17 (s, 3H).

^{13}C NMR (75 MHz, $CDCl_3$) δ 190.6 (C), 159.2 (C), 141.8 (C), 136.4 (CH), 134.6 (C), 130.5 (C), 130.0 (2 x CH), 127.9 (2 x CH), 127.8 (CH), 127.8 (2 x CH), 126.7 (CH), 124.6 (q, $J_{C\text{-}F}$ = 3.8 Hz, 2 x CH), 121.7 (CH), 121.4 (C), 118.2 (CH), 103.7 (C), 90.3 (C), 85.6 (C), 50.8 (CH), 44.5 (CH_2), 26.1 (3 x CH_3), 16.7 (C), - 4.6 (2 x CH_3). (CF_3 not asigned due to partial occlusion of the quartet of signals under aromatic carbons)

HRMS (ESI) m/z $[M + H]^+$ Calcd. for $C_{31}H_{32}F_3O_2Si$ 521.2118; Found 521.2133.

(R*)-2-[(R*)-1-phenyl-3-(triisopropylsilyl)-1-prop-2-yn-1-yl]chroman-4-one (4l)

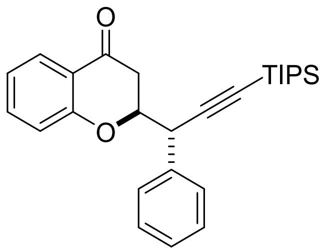

Yield: 73%, 61 mg

Colorless oil

$R_f(SiO_2)$ = 0.14 (Hexanes/ Ethyl acetate, (20:1)).

^{1}H NMR (300 MHz, $CDCl_3$) δ 7.89 (dd, J = 7.8, 1.8 Hz, 1H), 7.57-7.26 (m, 6H), 7.11-6.93 (m, 2H), 4.61 (ddd, J = 12.9, 5.7, 2.8 Hz, 1H), 4.37 (d, J = 5.7 Hz, 1H), 3.05 (dd, J = 16.8 and 12.9 Hz, 1H), 2.72 (dd, J = 16.8 and 2.9 Hz, 1H), 1.28-0.93 (m, 3H), 1.12 (s, 18H).

^{13}C NMR (75 MHz, $CDCl_3$) δ 192.2 (C), 161.2 (C), 136.3 (C), 136.1 (CH), 128.6 (2 x CH), 128.5 (2 x CH), 127.7 (CH), 126.9 (CH), 120.9 (C), 121.5 (CH), 118.0 (CH), 104.2 (C), 87.9 (C), 80.5 (CH), 43.9 (CH), 39.3 (CH_2), 18.7 (6 x CH_3), 11.2 (3 x CH).

HRMS (ESI) m/z $[M + H]^+$ Calcd. for $C_{27}H_{35}O_2Si$ 419.2401; Found 419.2401.

3. Experimental procedure for the one-pot synthesis of tricyclic compounds 5.

Method A: In a polypropylene Falcon^{TM}-type tube inside a schlenck, and under argon atmosphere at 25ºC, 0.2 mmol of chromone **1** and 0.3 mmol (1.5 equivalents) of propargylsilane **2** (3 equivalents for **5e**) were dissolved in 1mL of dry diethyl ether. Over this solution, 2.25 mg (1 mol%) of phosphite gold catalyst were added. After 1 hour of stirring at 25ºC, 3.9 mg (5 mol%) of dichloro(2-pyridinecarboxylate)gold catalyst, dissolved in 3 mL of dry 1,4-dioxane, were added and the mixture was stirred for 48 hours. After that period, 161 μL (128 mg; 4 mmol; 20 equivalents) of methyl alcohol was added and the mixture stirred for additional 10 minutes. Removal of the solvents under vacuum and chromatographic purification of the residue through silica-gel, get raised to cyclopentenbenzopyranone derivatives **5**, obtained as pure compounds.

Method B: In a polypropylene Falcon^{TM}-type tube inside a schlenck, and under argon atmosphere at 25ºC, 0.2 mmol of chromone **1** and 0.3 mmol (1.5 equivalents) of propargylsilane **2** were added. Over this mixture, a solution of 5.6 mg (2.5 mol%) of phosphite gold catalyst dissolved in 1 mL of dry diethyl ether and 3.9 mg (5 mol%) of dichloro(2-pyridinecarboxylate)gold, dissolved in 3 mL of dry 1,4-dioxane, were added. The mixture was stirred for 48 hours. At this point, experimental procedure continues as described in Method A.

***cis,cis*-1-(*tert*-butyldimethylsilyl)-3-phenyl-3a,9a-dihydrocyclopenta[*b*]chromen-9(3*H*)-one (5a)**

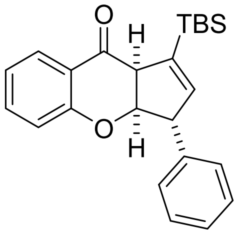

Yield: 81%, 61 mg

Colorless oil

$R_f (SiO_2)$ = 0.38 (Hexanes/ Ethyl acetate, (20:1)).

^{1}H NMR (300 MHz, $CDCl_3$) δ 7.91 (dd, *J* = 7.8 and 1.8 Hz, 1H), 7.48 (ddd, *J* = 8.6, 7.2 and 1.8 Hz, 1H), 7.42-7.13 (m, 5H), 7.12-6.89 (m, 2H), 6.27-6.14 (m, 1H), 4.93 (dt, *J* = 5.1 and 1.0 Hz, 1H), 4.43 (d, *J* = 1.4 Hz, 1H), 3.90 (ddd, *J* = 5.1, 2.0 and 1.0 Hz, 1H), 0.85 (s, 9H), 0.09 (s, 3H), - 0.29 (s, 3H).

^{13}C NMR (75 MHz, $CDCl_3$) δ 190.5 (C), 161.1 (C), 148.2 (C), 138.5 (C), 138.5 (CH), 136.3 (CH), 128.8 (2 x CH), 127.9 (2 x CH), 127.3 (CH), 127.0 (CH), 121.3 (CH), 120.6 (C), 118.1 (CH), 88.0 (CH), 64.7 (CH), 57.0 (CH), 26.6 (3 x CH_3), 17.1 (C), -5.5 (CH_3), - 5.9 (CH_3).

HRMS (ESI) m/z $[M + H]^+$ Calcd. for $C_{24}H_{29}O_2Si$ 377.1931; Found 377.1938.

***cis,cis*-7-bromo-1-(*tert*-butyldimethylsilyl)-3-phenyl-3a,9a-dihydrocyclopenta[*b*]chromen-9(3*H*)-one (5b)**

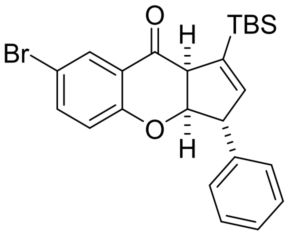

Yield: 92%, 84 mg

Colorless oil

$R_f (SiO_2)$ = 0.43 (Hexanes/ Ethyl acetate, (20:1)).

^{1}H NMR (300 MHz, $CDCl_3$) δ 8.00 (d, *J* = 2.5 Hz, 1H), 7.55 (dd, *J* = 8.8 and 2.6 Hz, 1H), 7.38-7.24 (m, 3H), 7.22-7.15 (m, 2H), 6.88 (d, *J* = 8.8 Hz, 1H), 6.14 (dt, *J* = 2.0 and 0.9 Hz, 1H), 4.93 (dt, *J* = 5.1, 1.0 Hz, 1H), 4.42 (s, 1H), 3.89 (ddd, *J* = 5.1, 2.0, 1.0 Hz, 1H), 0.83 (s, 9H), 0.08 (s, 3H), - 0.31 (s, 3H).

^{13}C NMR (75 MHz, $CDCl_3$) δ 189.3 (C), 159.9 (C), 148.7 (C), 138.9 (CH), 138.1 (C), 137.9 (CH), 129.4 (CH), 128.8 (2 x CH), 127.9 (2 x CH), 127.5 (CH), 121.7 (C), 120.2 (CH), 113.8 (C), 88.2 (CH), 64.6 (CH), 56.6 (CH), 26.6 (3 x CH_3), 17.1 (C), - 5.5 (CH_3), - 5.9 (CH_3).

HRMS (ESI) m/z $[M + Na]^+$ Calcd. for $C_{24}H_{27}BrNaO_2Si$ 477.0856; Found 477.0869.

***cis,cis*-1-(*tert*-butyldimethylsilyl)-5-methyl-3-phenyl-3a,9a-dihydrocyclopenta[*b*]chromen-9(3*H*)-one (5c)**

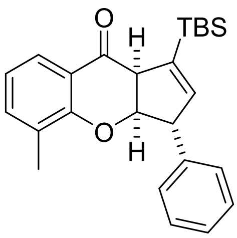

Yield: 73%, 57 mg

Colorless oil

R_f (SiO_2) = 0.33 (Hexanes/ Ethyl acetate, (30:1)).

^{1}H NMR (300 MHz, $CDCl_3$) δ 7.73 (dd, *J* = 8.1 and 1.8 Hz, 1H), 7.41-7.14 (m, 6H), 6.88 (t, *J* = 7.6 Hz, 1H), 6.21-6.11 (m, 1H), 4.92 (dt, *J* = 5.1 and 1.1 Hz, 1H), 4.41 (d, *J* = 1.4 Hz, 1H), 3.86 (ddd, *J* = 5.1, 2.1 and 1.0 Hz, 1H), 2.24 (s, 3H), 0.81 (s, 9H), 0.07 (s, 3H), - 0.33 (s, 3H).

^{13}C NMR (75 MHz, $CDCl_3$) δ 190.9 (C), 159.2 (C), 148.2 (C), 138.6 (CH), 137.1 (CH), 128.7 (2 x CH), 128.0 (2 x CH), 127.3 (CH), 124.6 (CH), 120.5 (CH), 120.1 (C), 87.8 (CH), 64.6 (CH), 56.6 (CH), 26.6 (3 x CH_3), 17.1 (C), 15.8 (CH_3), - 5.6 (CH_3), - 6.0 (CH_3).

HRMS (ESI) m/z $[M + H]^+$ Calcd. for $C_{25}H_{31}O_2Si$ 391.2088; Found 391.2083.

***cis,cis*-1-(*tert*-butyldimethylsilyl)-9-oxo-3-phenyl-3,3a,9,9a-tetrahydrocyclopenta[*b*]chromen-6-yl benzoate (5d)**

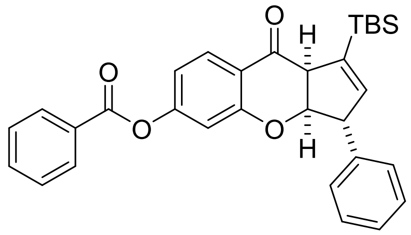

Yield: 75%, 59 mg

Colorless oil

R_f (SiO_2) = 0.46 (Hexanes/ Ethyl acetate, (10:1)).

^{1}H NMR (300 MHz, $CDCl_3$) δ 8.19 (dt, *J* = 7.2 and 1.4 Hz, 2H), 7.96 (d, *J* = 9.1 Hz, 1H), 7.65 (d, *J* = 7.5 Hz, 1H), 7.60-7.47 (m, 2H), 7.39-7.22 (m, 3H), 7.22-7.13 (m, 2H), 6.89 (dt, *J* = 4.1 and 2.1 Hz, 2H), 6.17 (dt, *J* = 1.9, 0.9 Hz, 1H), 4.96 (dd, *J* = 5.1 and 1.0 Hz, 1H), 4.41 (s, 1H), 3.95-3.80 (m, 1H), 2.17 (s, 1H), 0.83 (s, 9H), 0.07 (s, 3H), - 0.31 (s, 3H).

^{13}C NMR (75 MHz, $CDCl_3$) δ 189.5 (C), 164.3 (C), 162.1 (C), 157.1 (C), 148.3 (C), 138.3 (CH), 134.0 (CH), 130.3 (2 x CH), 129.0 (C), 128.8 (2 x CH), 128.7 (2 x CH), 128.6 (CH), 127.9 (2 x CH), 127.4 (CH), 118.5 (C), 115.5 (CH) 111.13 (CH), 88.6 (CH), 64.6 (CH), 56.8 (CH), 26.6 (3 x CH_3), 17.1 (C), - 5.5 (CH_3), - 5.9 (CH_3).

HRMS (ESI) m/z $[M + H]^+$ Calcd. for $C_{31}H_{33}O_4Si$ [M+H]: Calc.: 497.2143; Found 497.2140.

***cis,cis*-1-(*tert*-butyldimethylsilyl)-3-(*p*-tolyl)-3a,9a-dihydrocyclopenta[*b*]chromen-9(3*H*)-one (5e)**

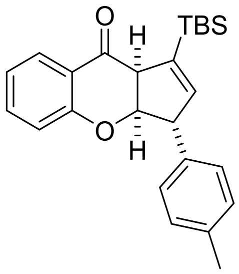

Yield: 72%, 56 mg

Colorless oil

$R_f(SiO_2)$ = 0.33 (Hexanes/ Ethyl acetate, (30:1)).

^{1}H NMR (300 MHz, $CDCl_3$) δ 7.88 (dd, *J* = 7.9 and 1.8 Hz, 1H), 7.46 (ddd, *J* = 8.8, 7.3 and1.8 Hz, 1H), 7.19-6.88 (m, 6H), 6.21-6.07 (m, 1H), 4.95-4.80 (m, 1H), 4.38 (d, *J* = 1.4 Hz, 1H), 3.86 (ddd, *J* = 5.1, 2.0 and 1.0 Hz, 1H), 2.33 (s, 3H), 0.82 (s, 9H), 0.06 (s, 3H), - 0.31 (s, 3H).

^{13}C NMR (75 MHz, $CDCl_3$) δ 190.7 (C), 161.1 (C), 148.4 (C), 138.2 (CH), 136.9 (C), 136.3 (CH), 135.3 (C), 129.4 (2 x CH), 127.8 (2 x CH), 127.0 (CH), 121.2 (CH), 120.6 (C), 118.1 (CH), 88.1 (CH), 64.2 (CH), 56.9 (CH), 26.6 (CH_3), 21.1 (3 x CH_3), 17.1 (C), - 5.5 (CH_3), - 5.8 (CH_3).

HRMS (ESI) m/z $[M + H]^+$ Calcd. for $C_{25}H_{31}O_2Si$ 391.2088; Found 391.2085.

4. NMR Spectra

^{1}H-NMR for previously reported starting compounds:

***Tert*-butyldimethyl(3-phenyl-3-(trimethylsilyl)prop-1-yn-1-yl)silane**

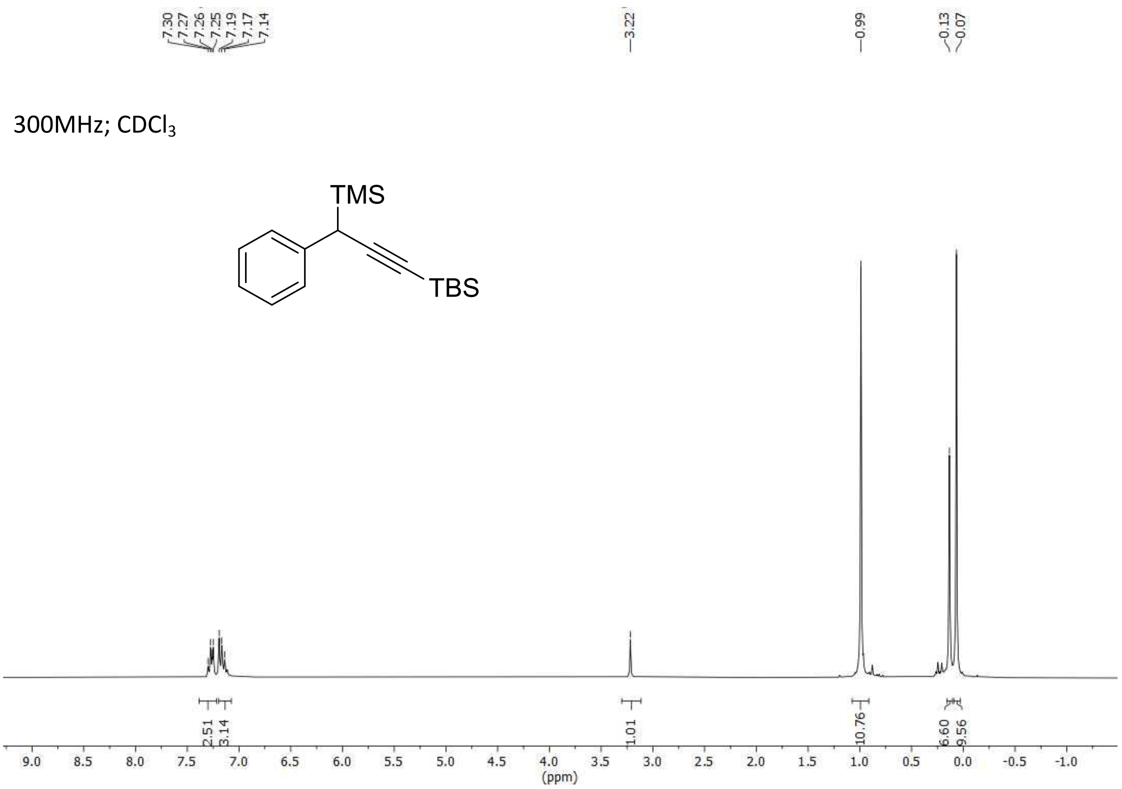

***Tert*-butyldimethyl(3-(*p*-tolyl)-3-(trimethylsilyl)prop-1-yn-1-yl)silane**

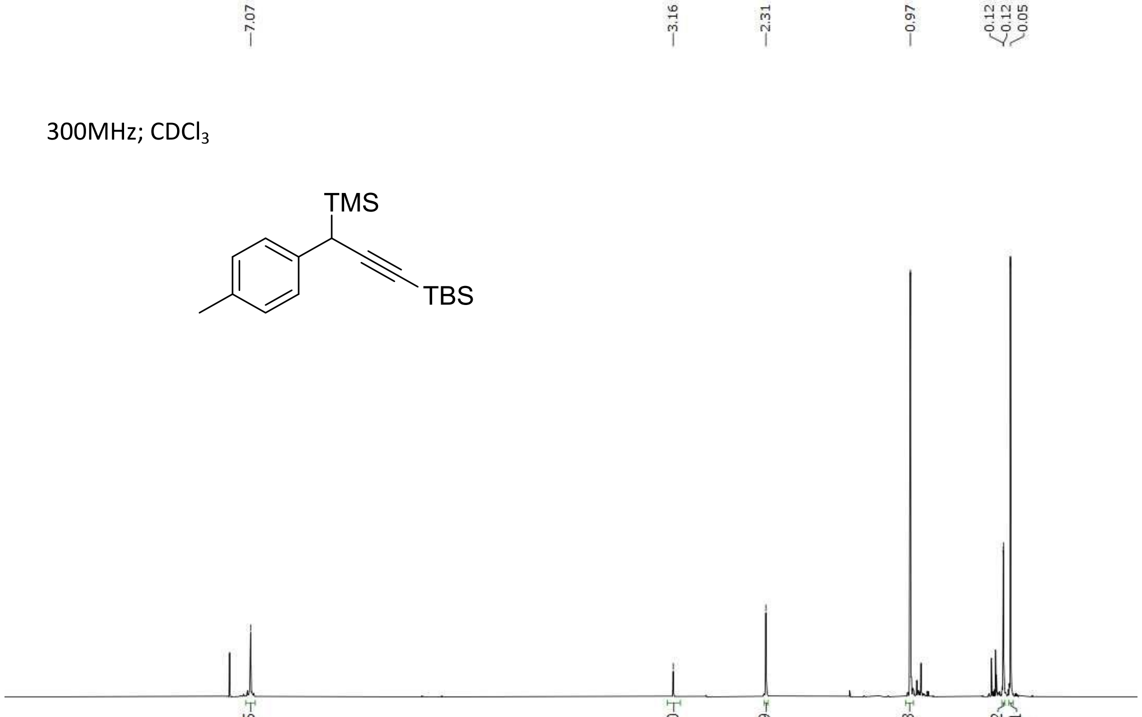

***Tert*-butyl(3-cyclopentyl-3-(trimethylsilyl)prop-1-yn-1-yl)dimethylsilane**

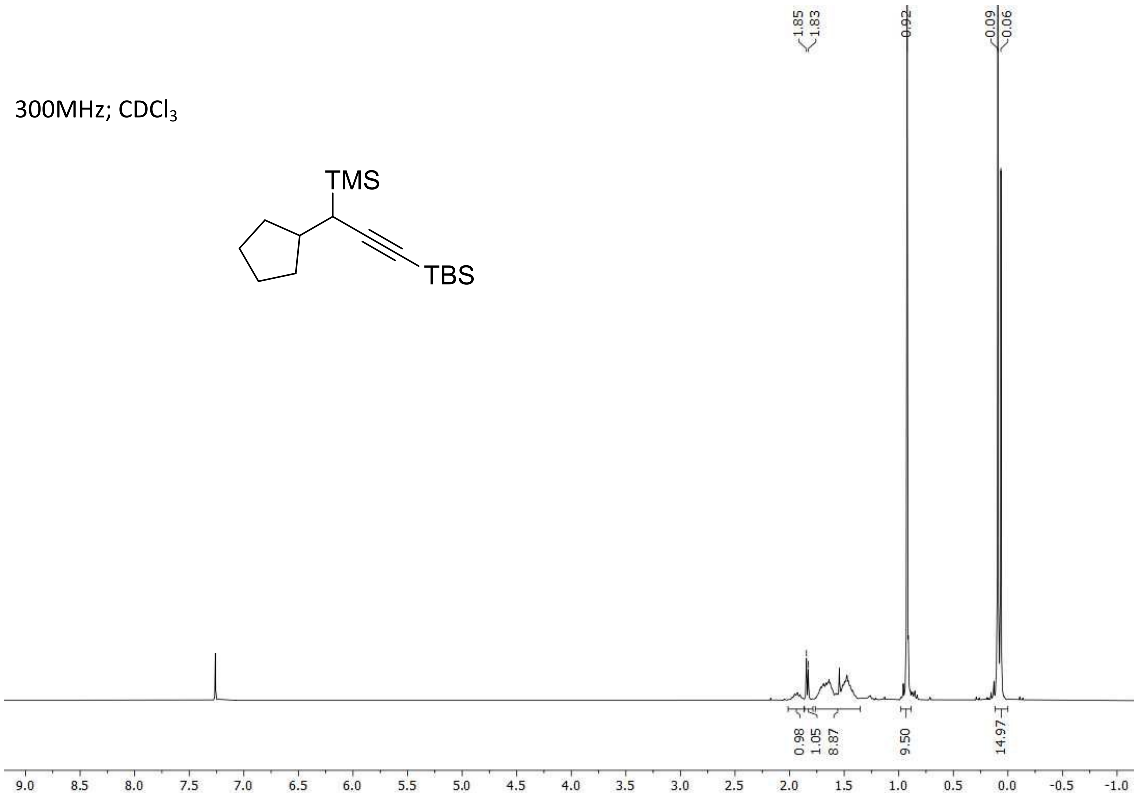

***Tert*-butyldimethyl(3-(trimethylsilyl)hept-1-yn-1-yl)silane**

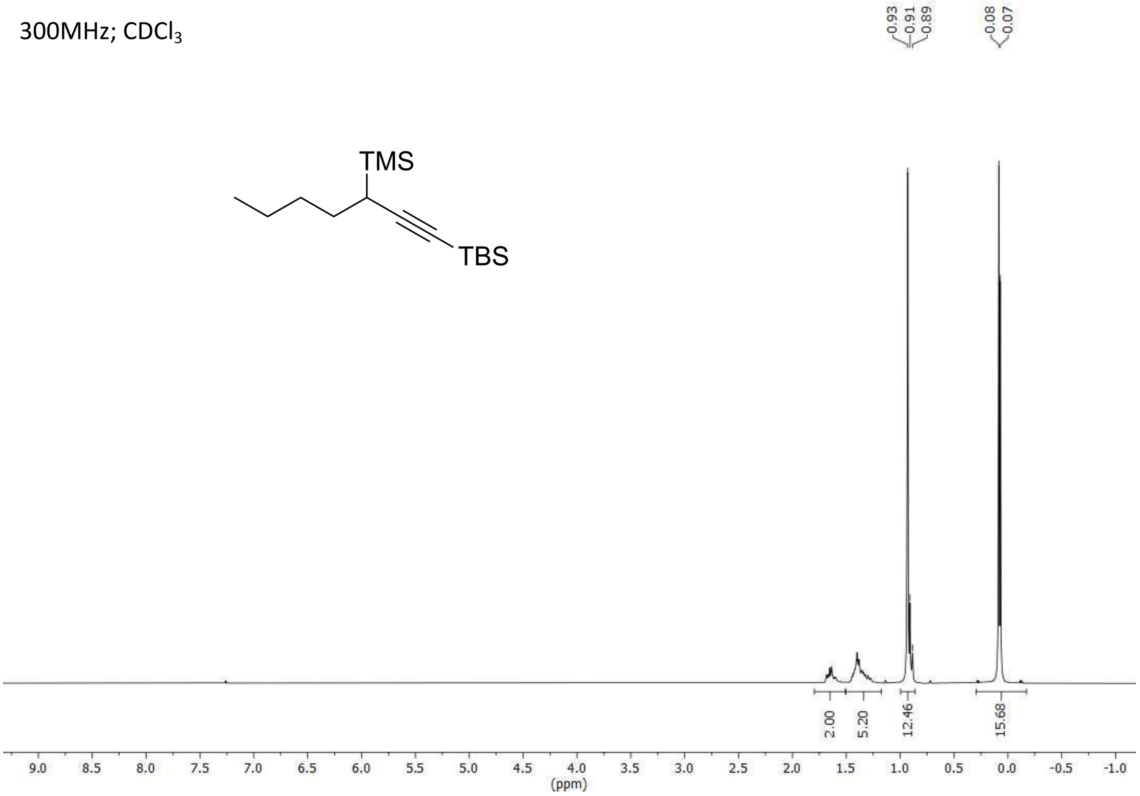

Triisopropyl(3-phenyl-3-(trimethylsilyl)prop-1-yn-1-yl)silane

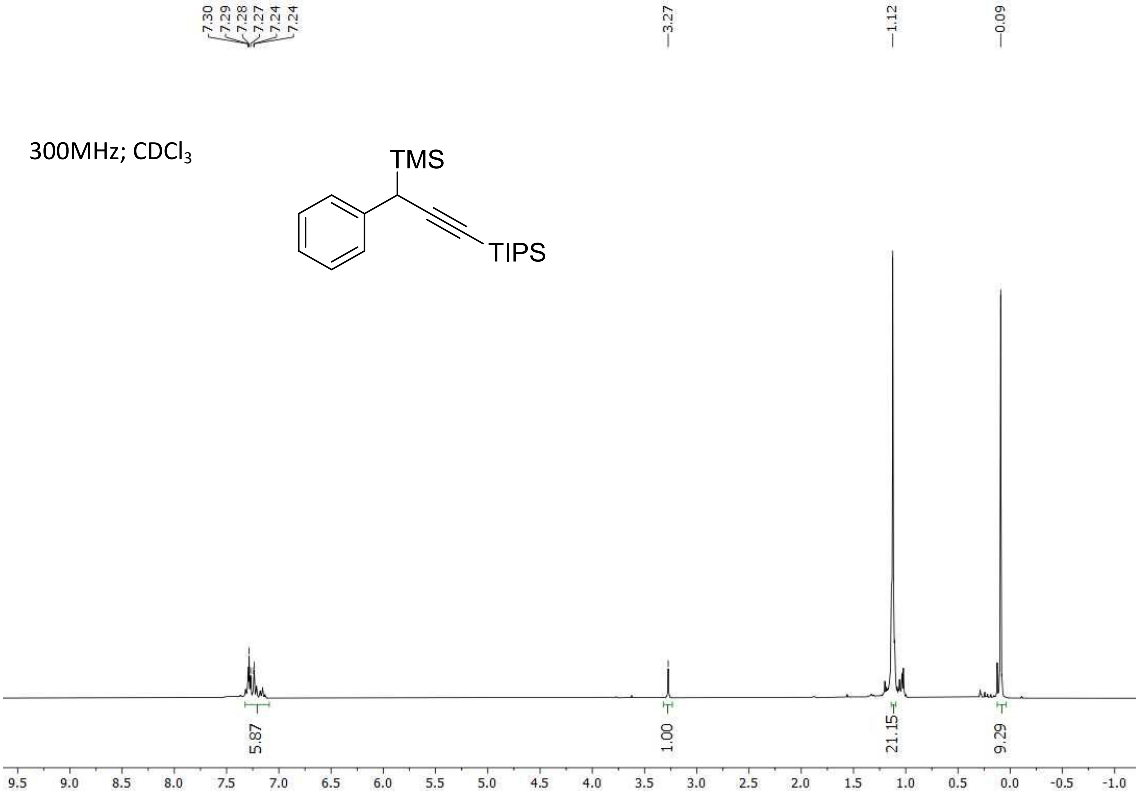

8-Methyl-4*H*-chromen-4-one

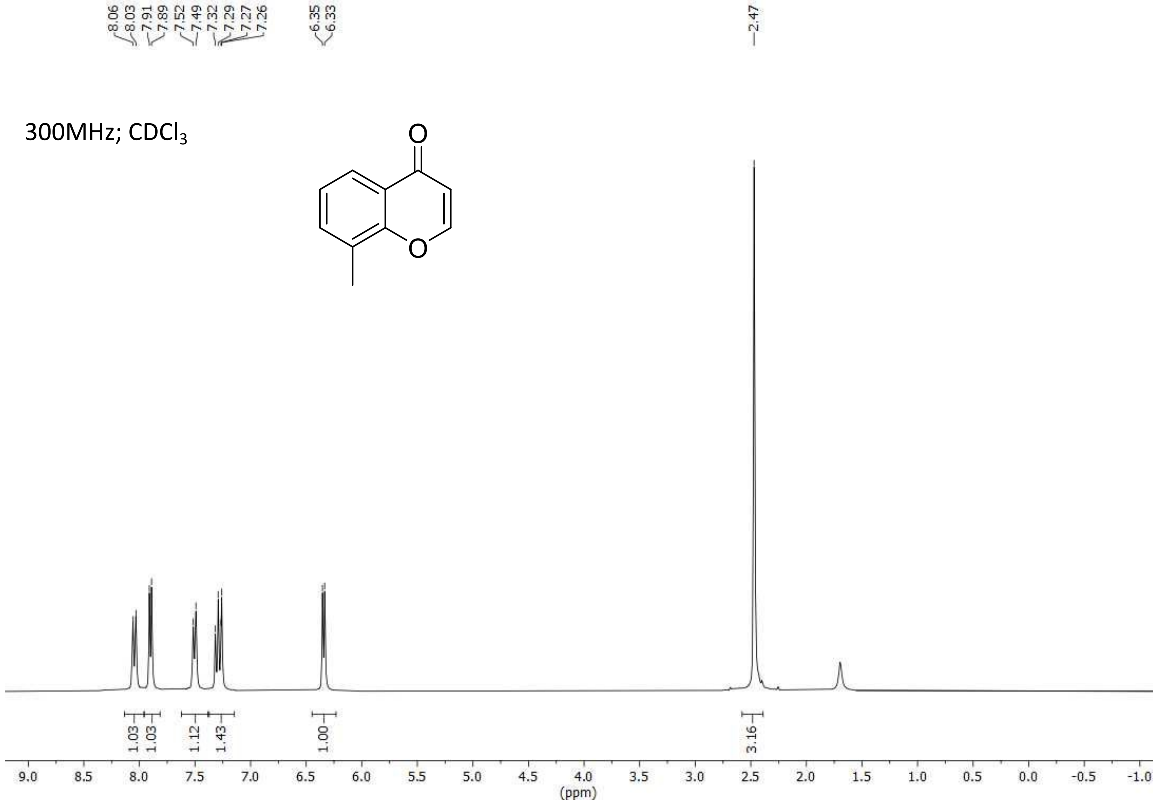

2-Methyl-4*H*-chromen-4-one

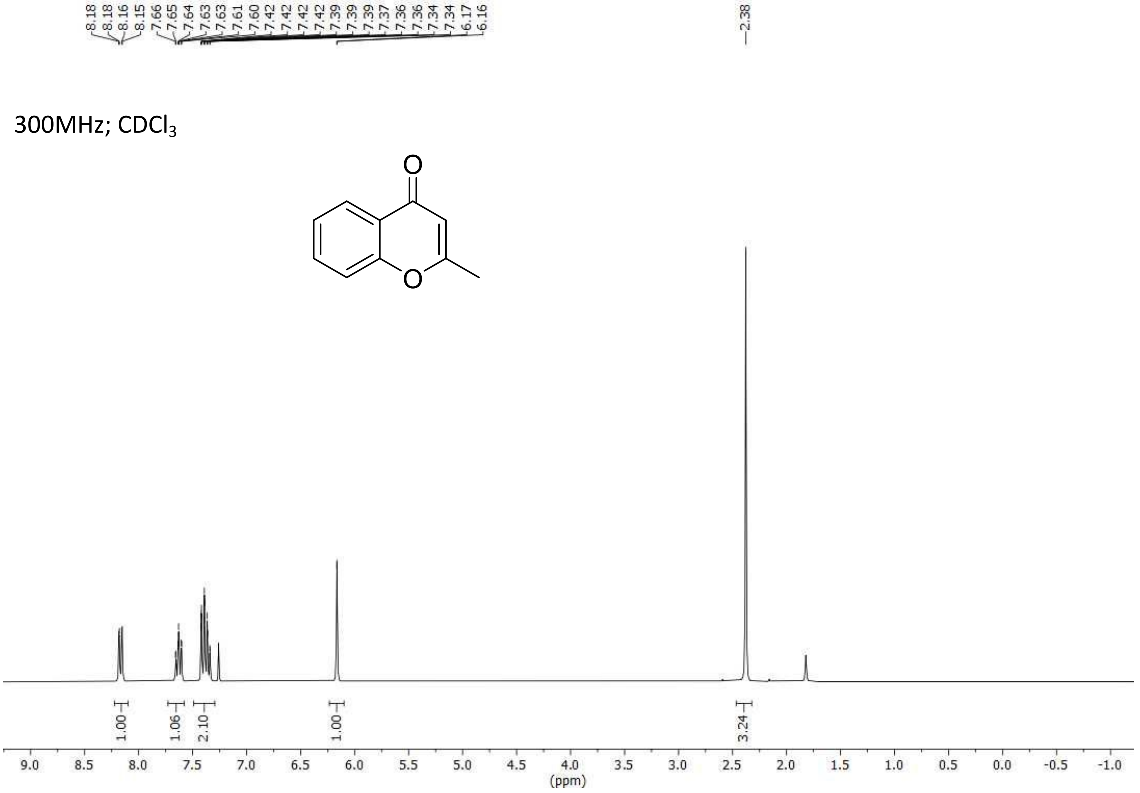

2-(4-(Trifluoromethyl)phenyl)-4*H*-chromen-4-one

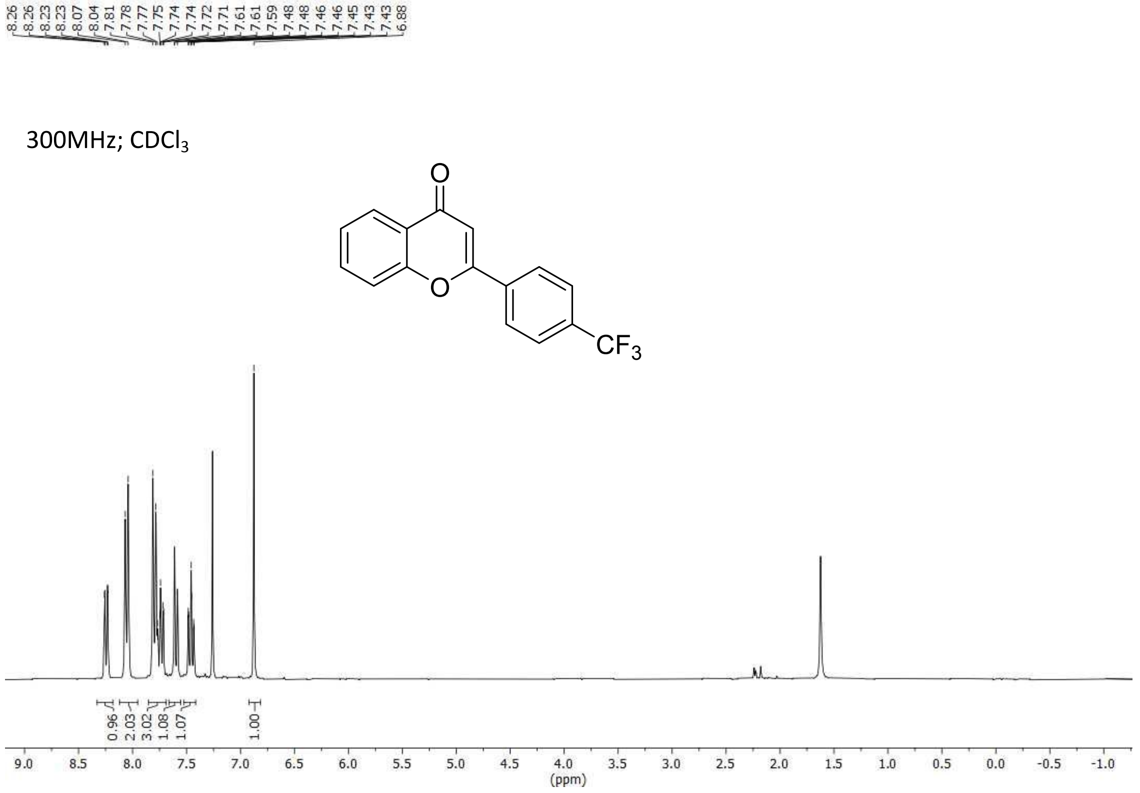

New compounds:

4-Oxo-4*H*-chromen-7-yl benzoate

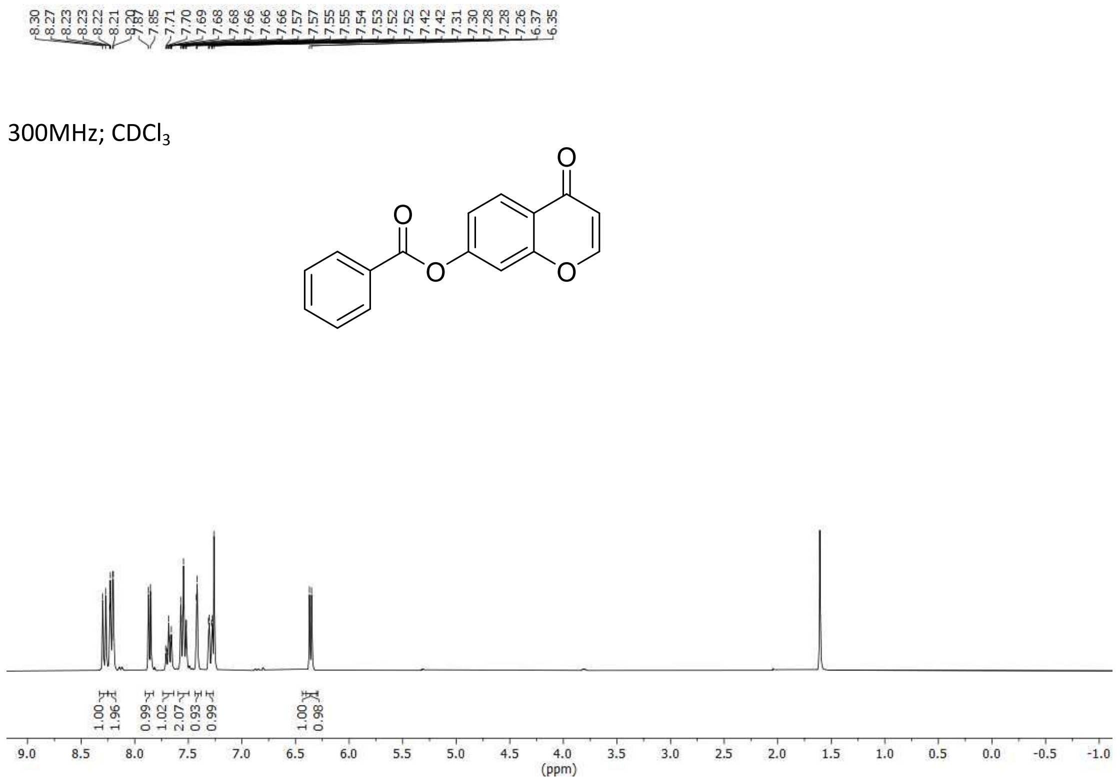

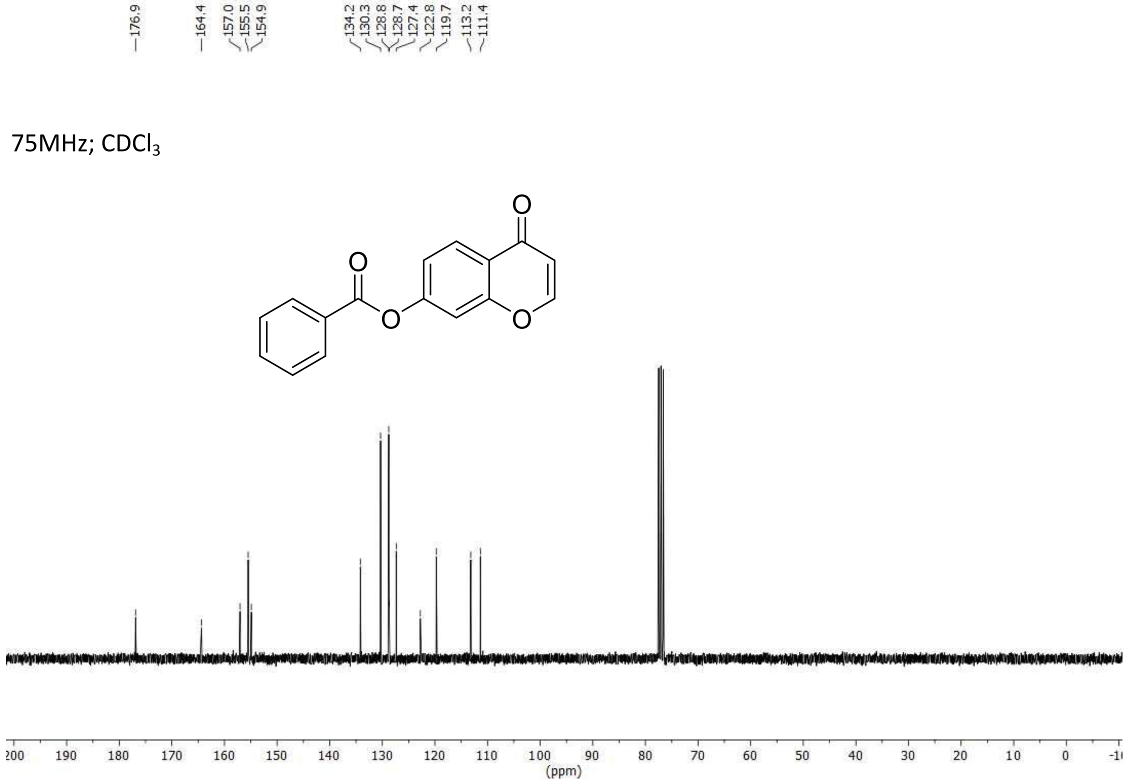

(R*)-2-[(R*)-3-(*tert*-butyldimethylsilyl)-1-phenylprop-2-yn-1-yl]chroman-4-one (4a)

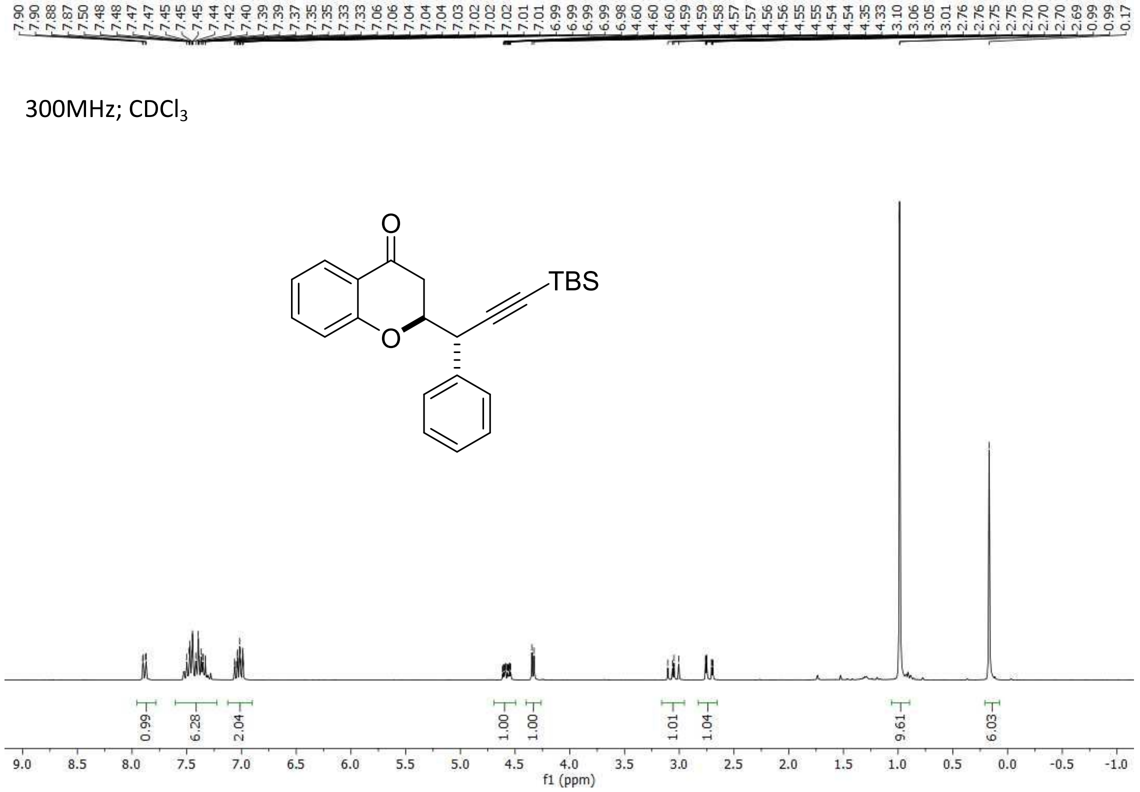

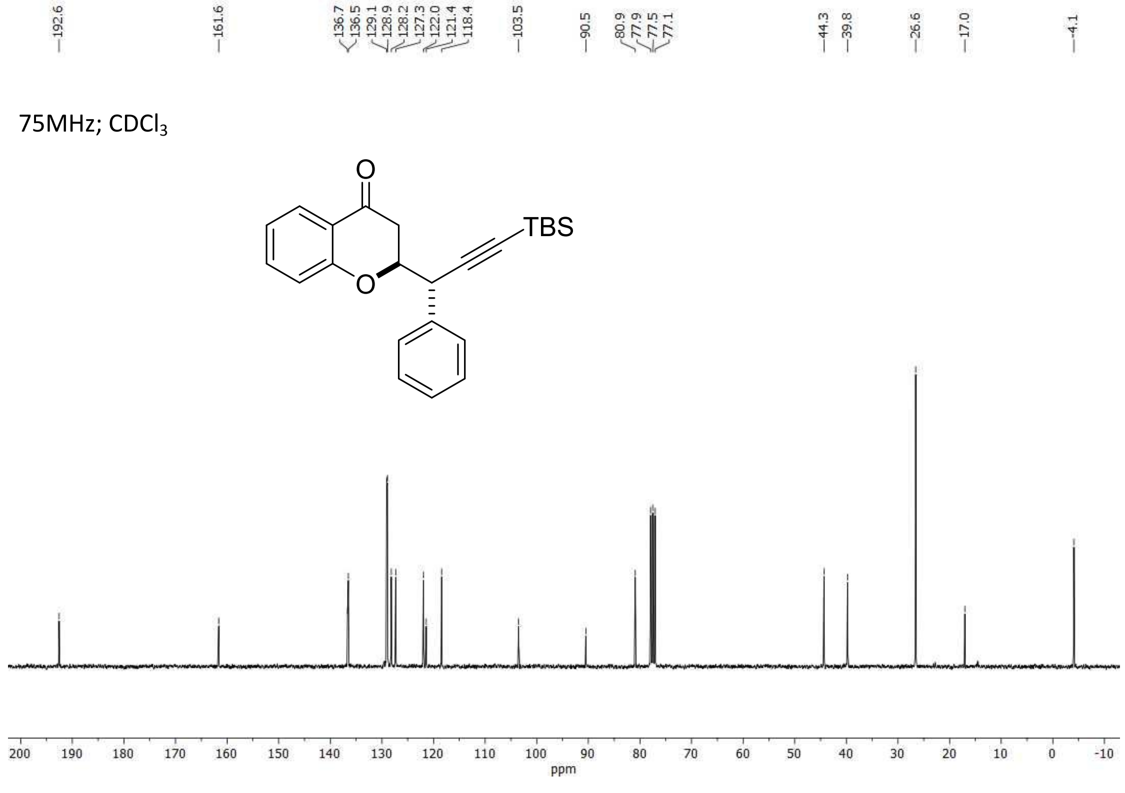

(R*)-2-[(R*)-3-(*tert*-butyldimethylsilyl)-1-(*p*-tolyl)prop-2-yn-1-yl]chroman-4-one (4b)

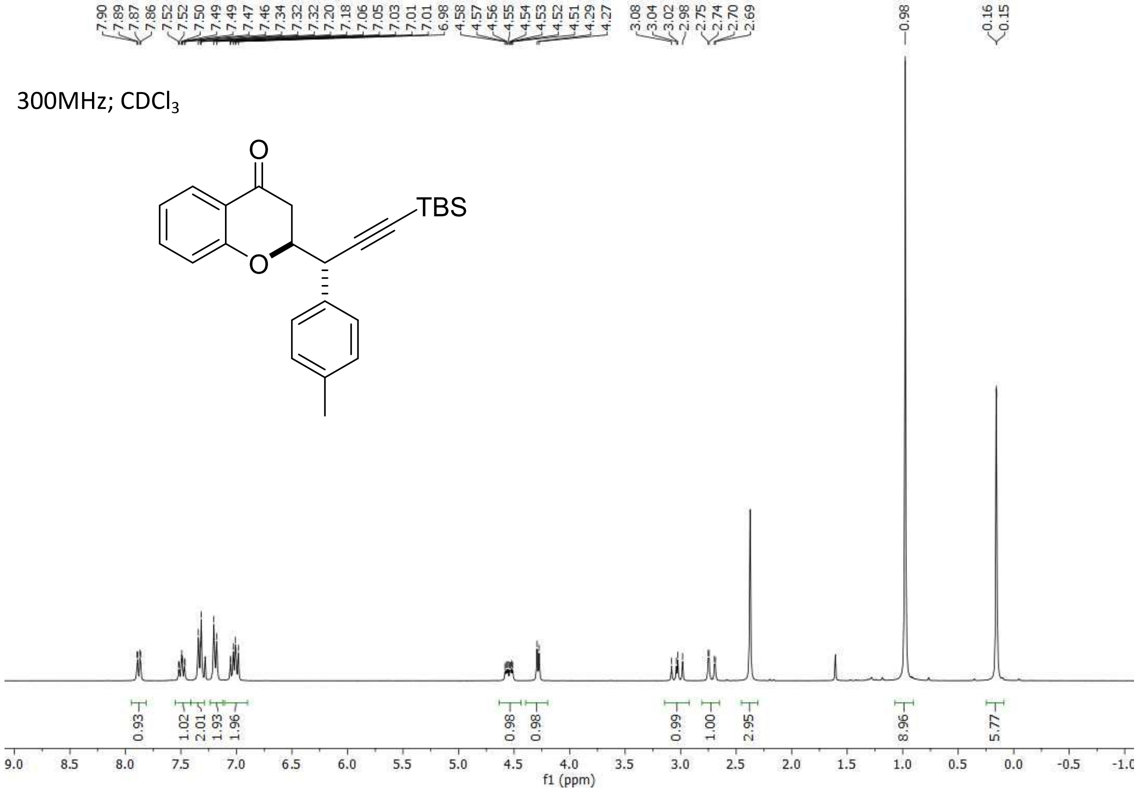

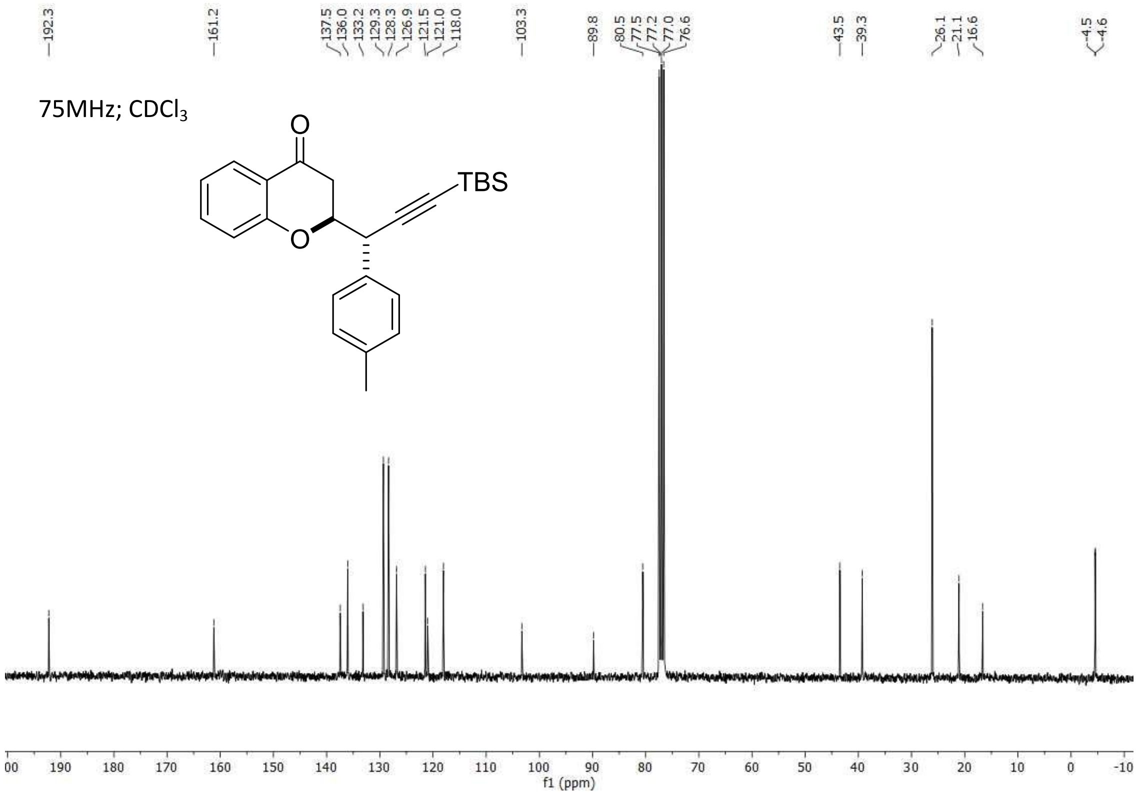

(R*)-2-[(R*)-3-(*tert*-butyldimethylsilyl)-1-cyclopentylprop-2-yn-1-yl]chroman-4-one (4c)

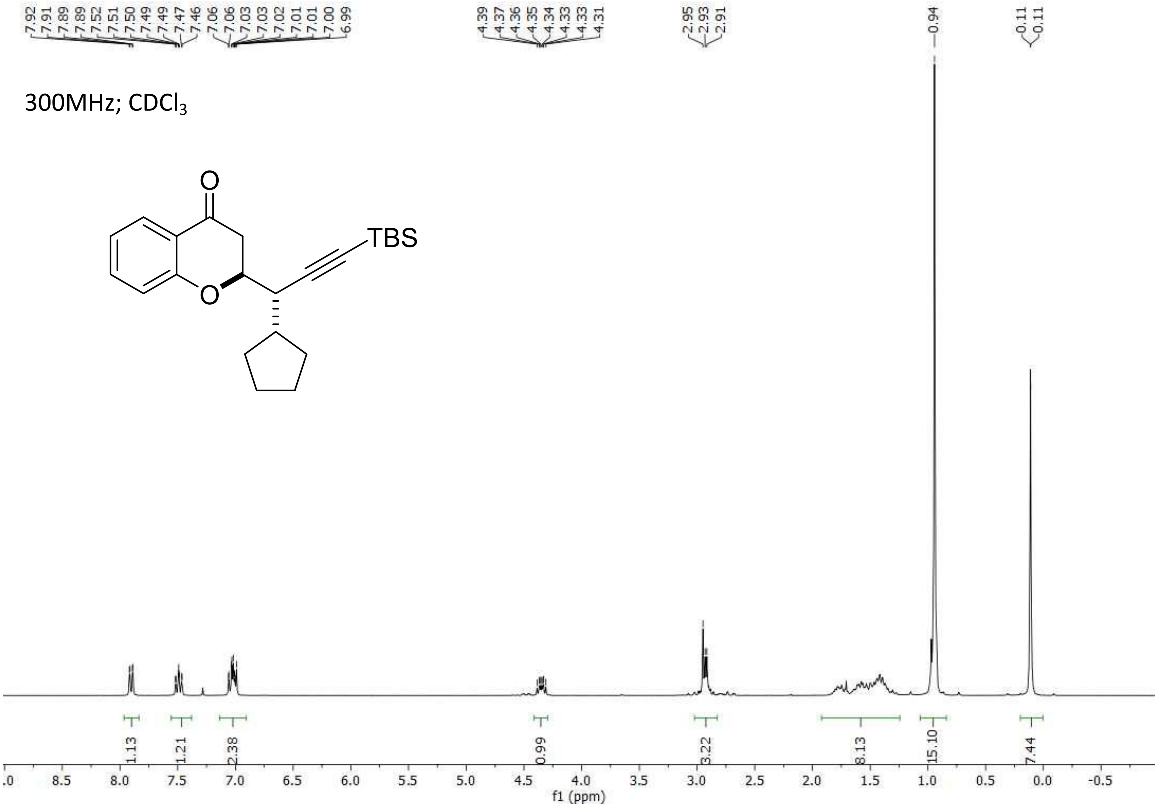

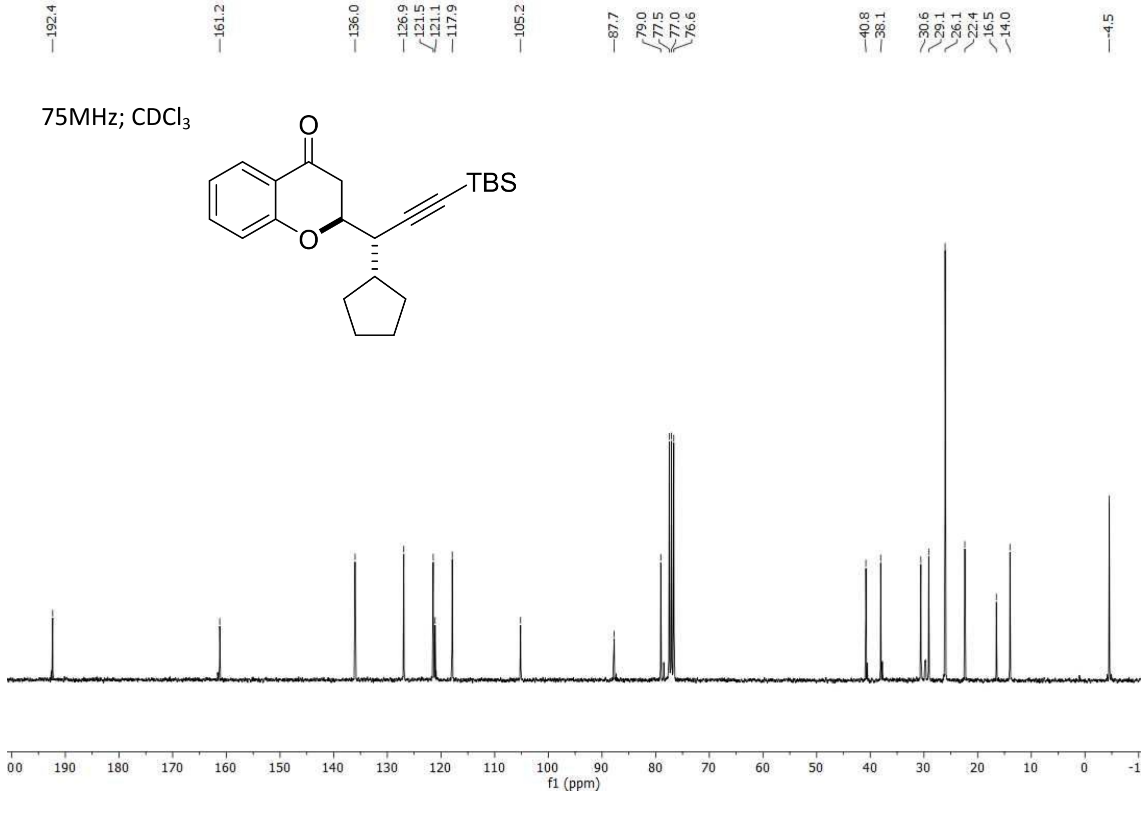

(R*)-2-[(R*)-1-(*tert*-butyldimethylsilyl)hept-1-yn-3-yl]chroman-4-one (4d)

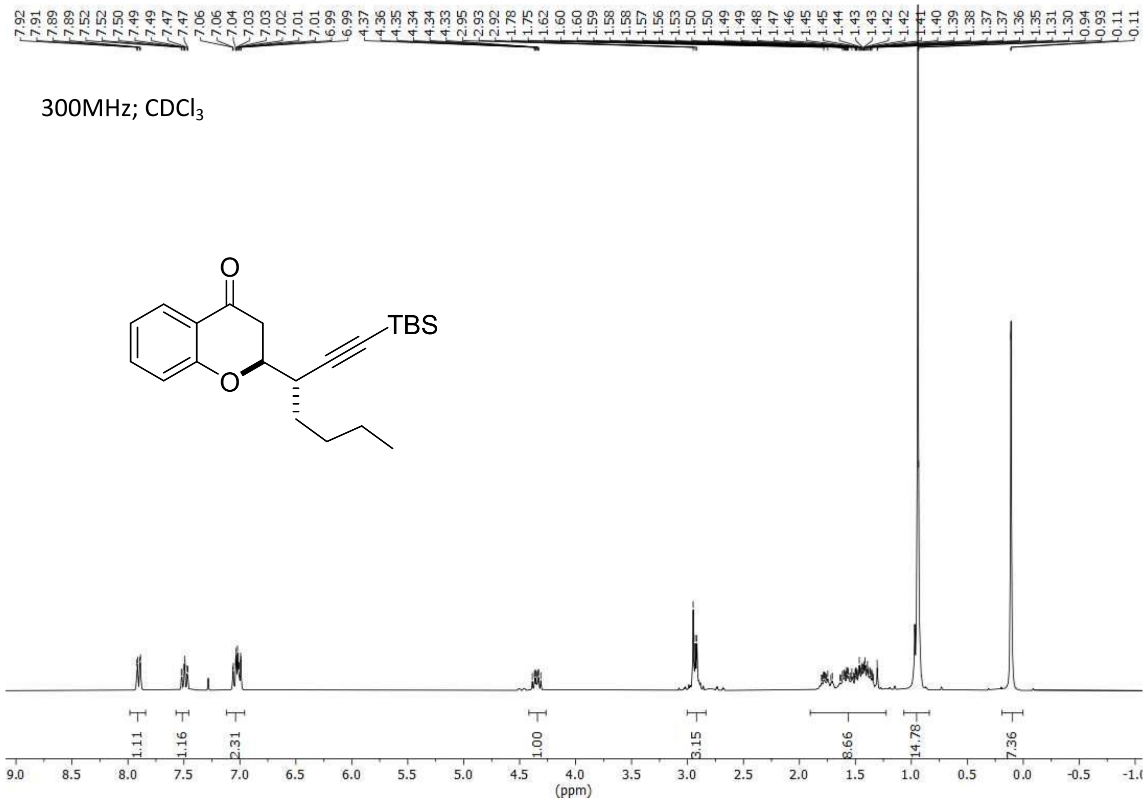

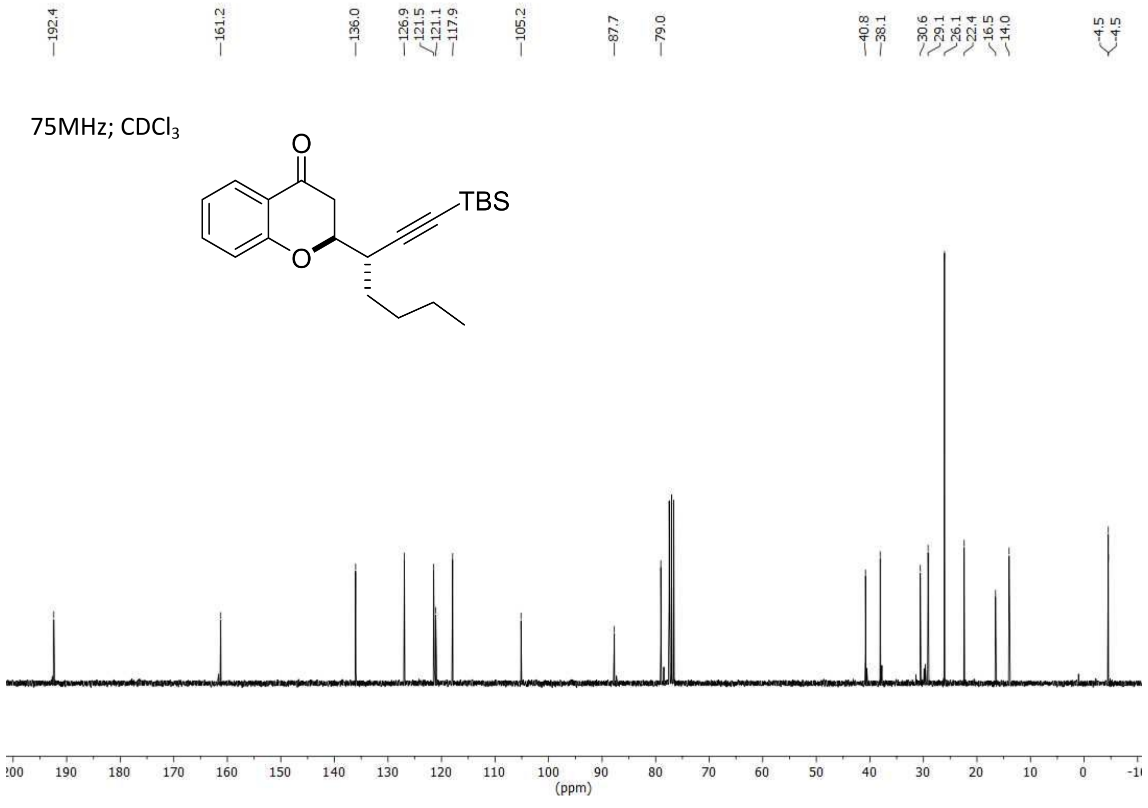

(R*)-6-bromo-2-[(R*)-3-(*tert*-butyldimethylsilyl)-1-phenylprop-2-yn-1-yl]chroman-4-one (4e)

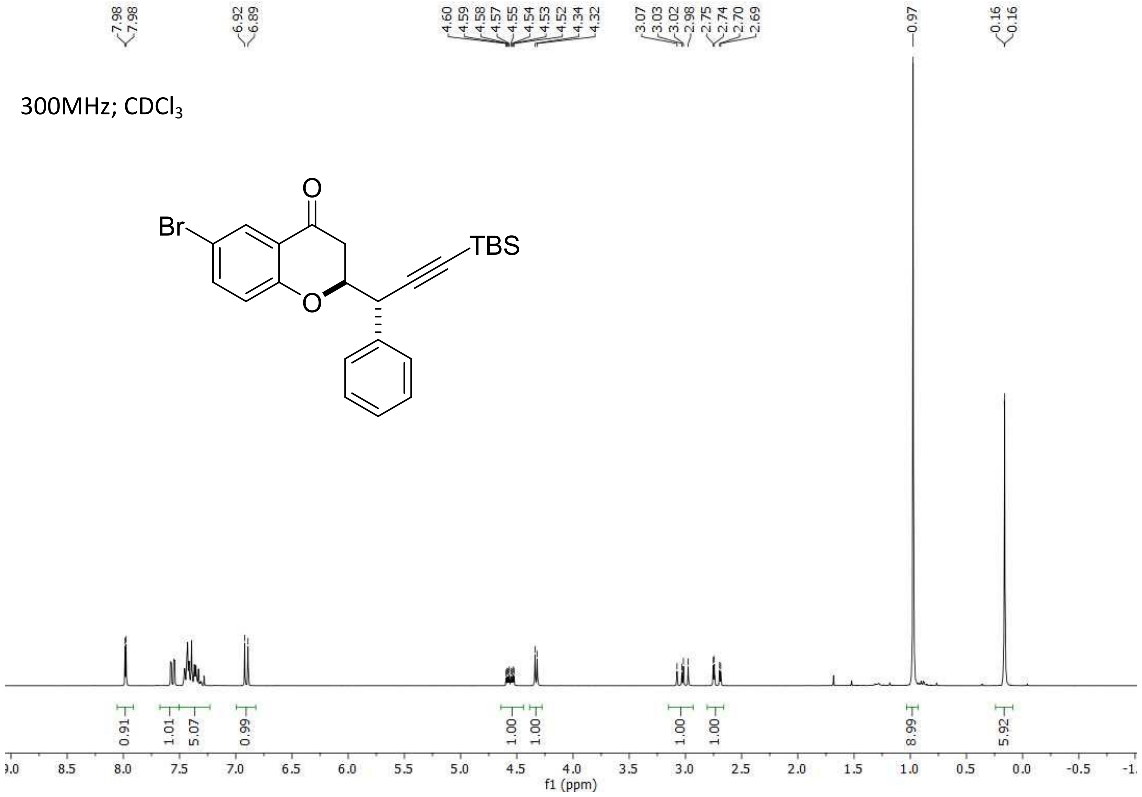

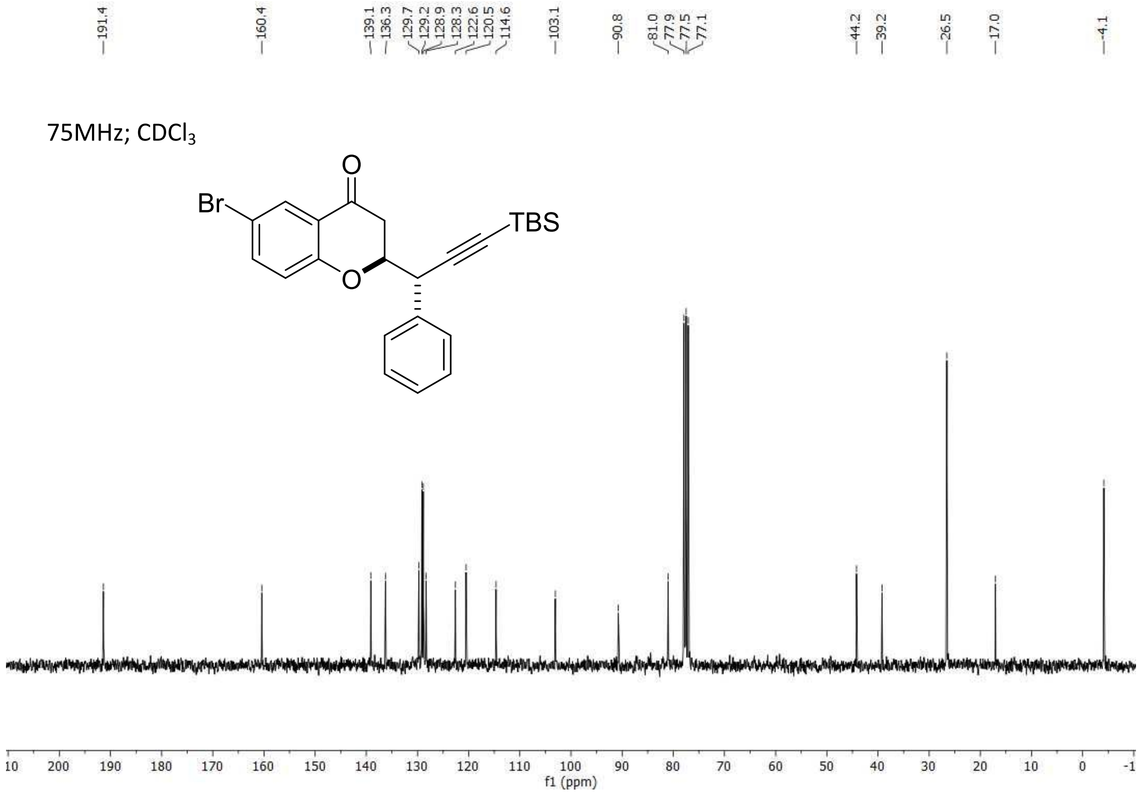

(R*)-2-[(R*)-3-(*tert*-butyldimethylsilyl)-1-phenylprop-2-yn-1-yl]-7-hydroxychroman-4-one (4f)

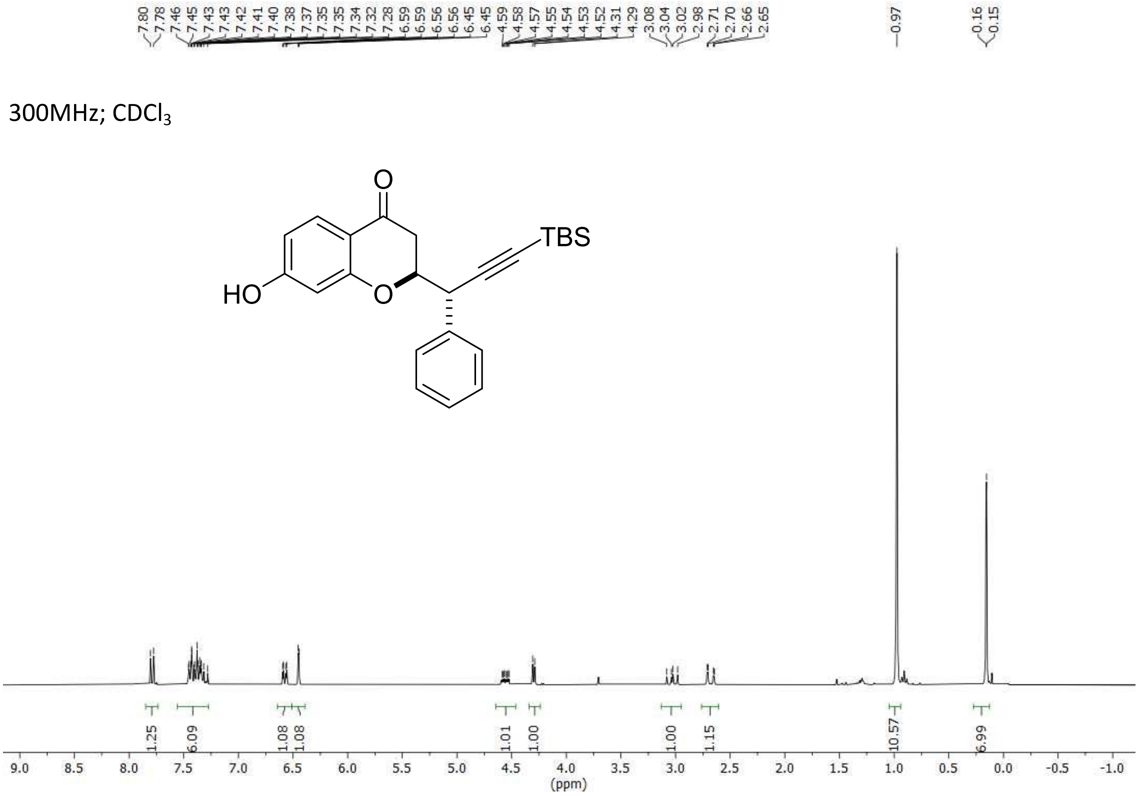

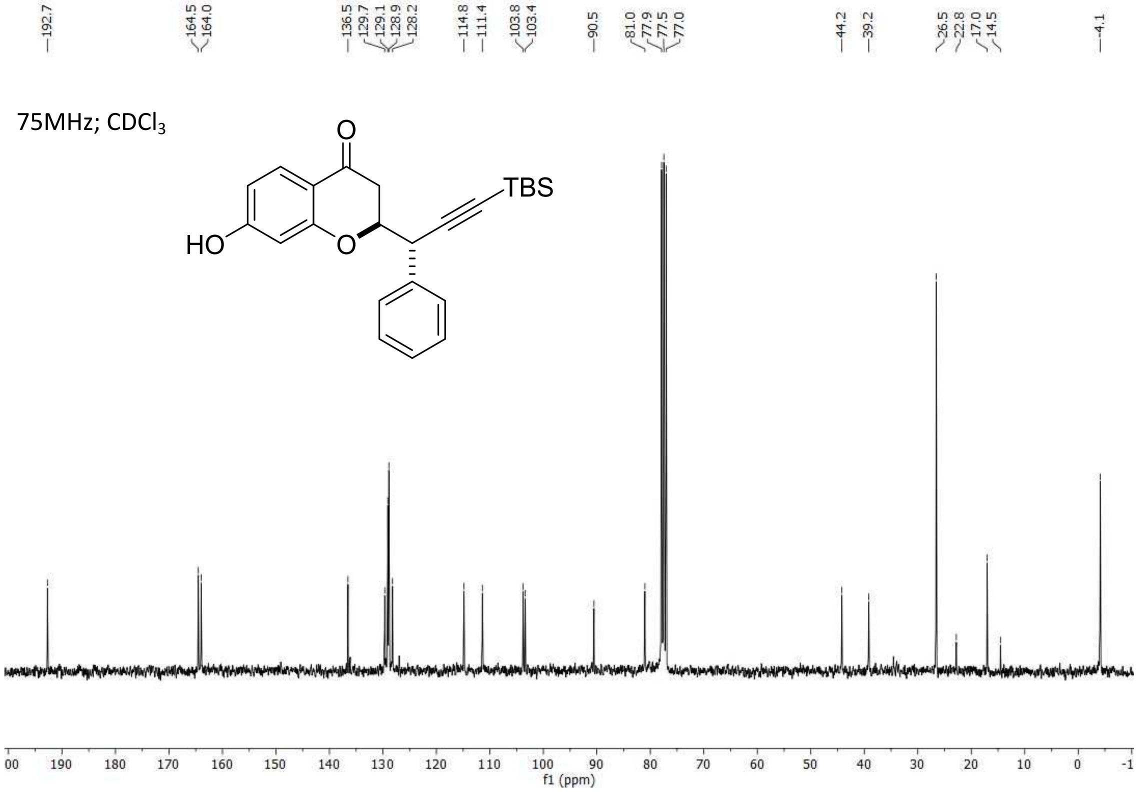

(R*)-2-[(R*)-3-(*tert*-butyldimethylsilyl)-1-phenylprop-2-yn-1-yl]-7-nitrochroman-4-one (4g)

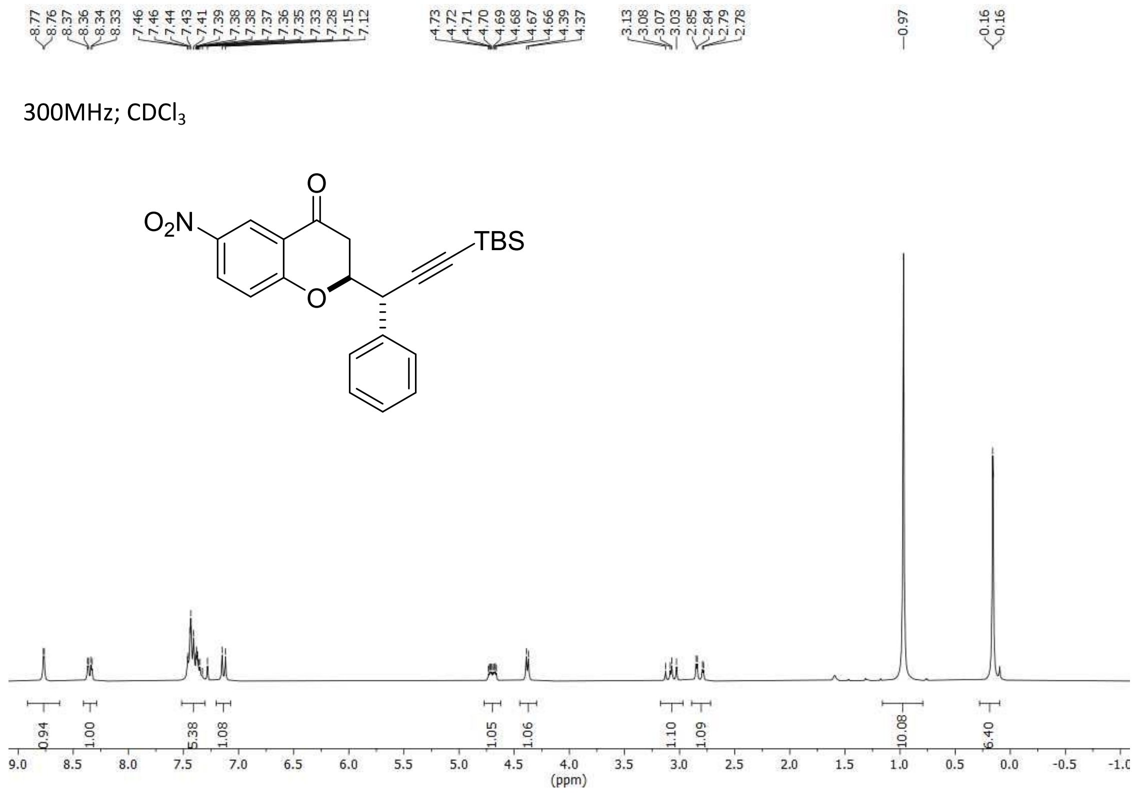

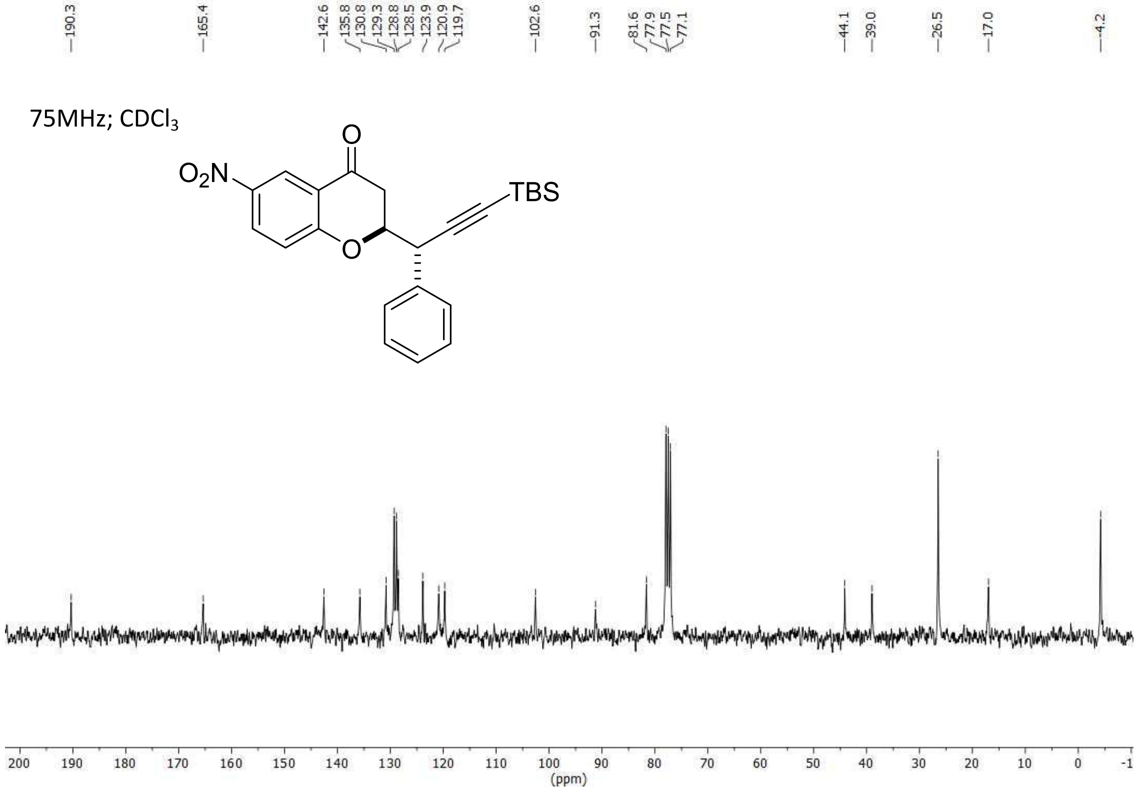

(R*)-2-[(R*)-3-(*tert*-butyldimethylsilyl)-1-phenylprop-2-yn-1-yl]-4-oxochroman-7-yl benzoate (4h)

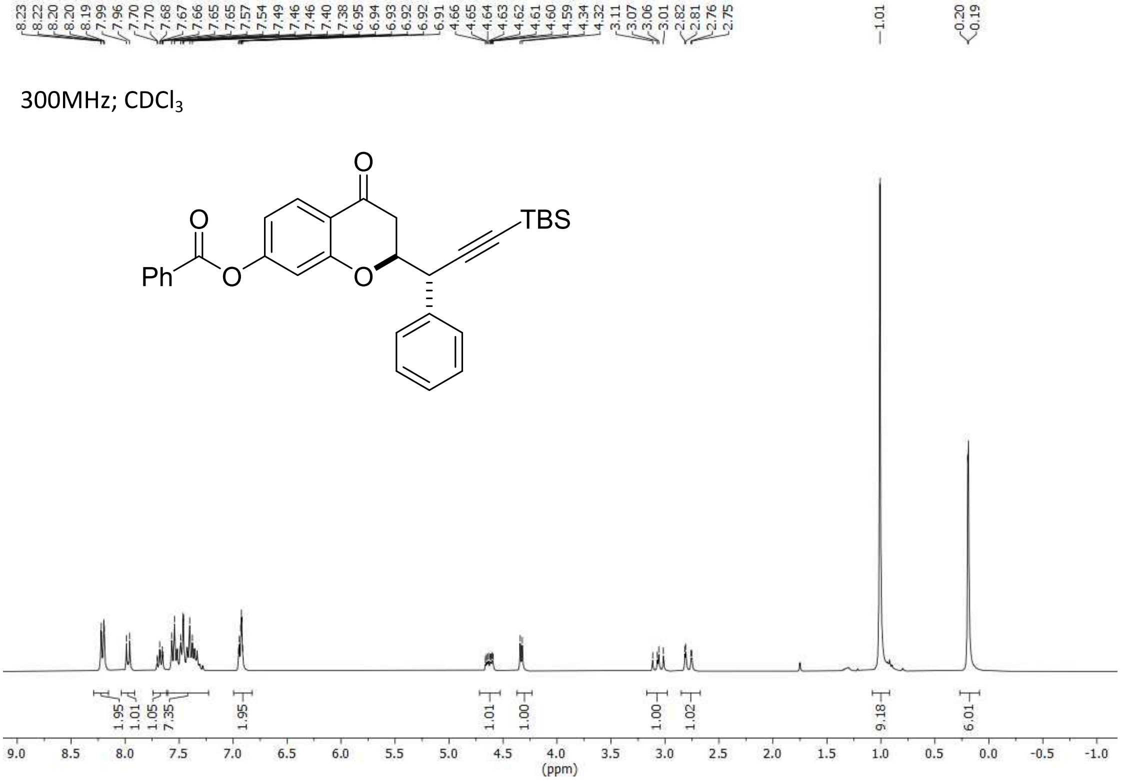

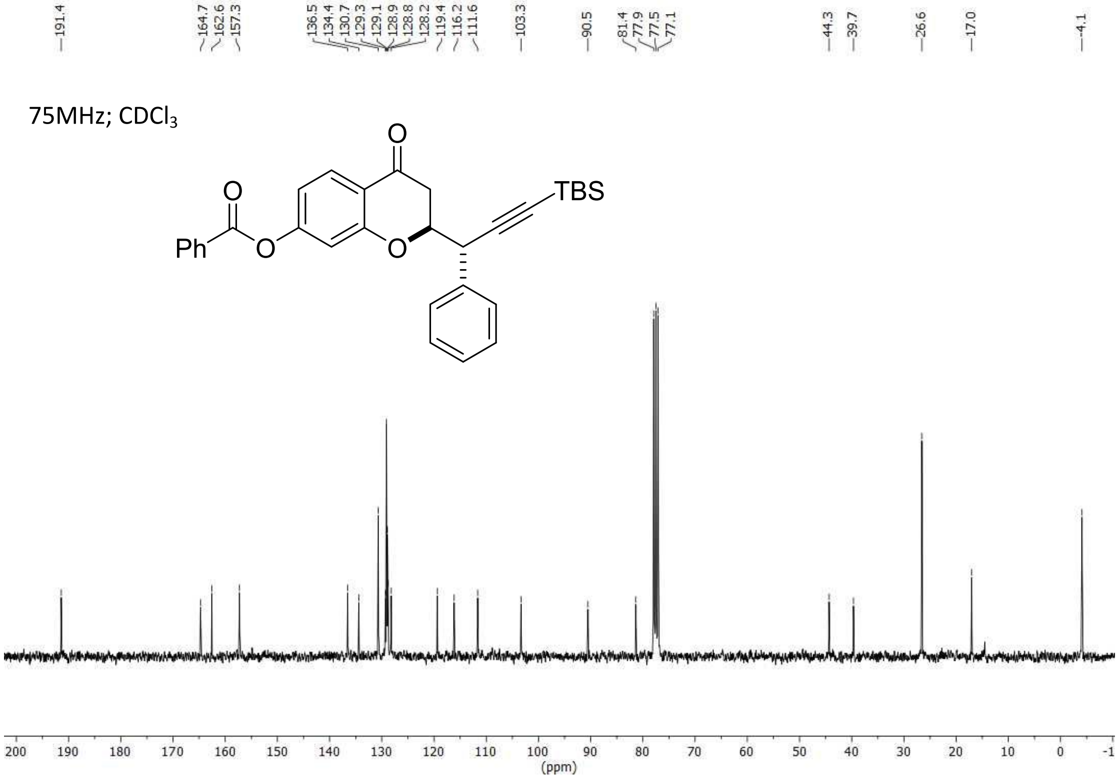

(R*)-2-[(R*)-3-(*tert*-butyldimethylsilyl)-1-phenylprop-2-yn-1-yl]-8-methylchroman-4-one (4i)

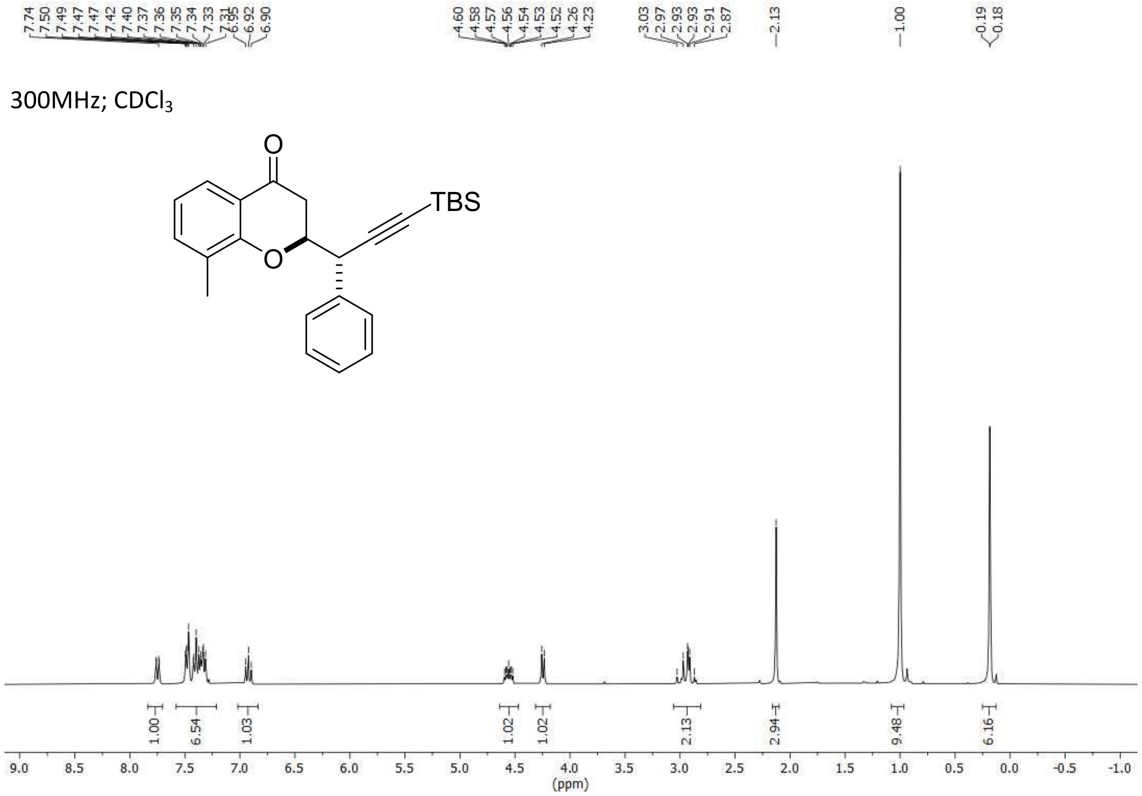

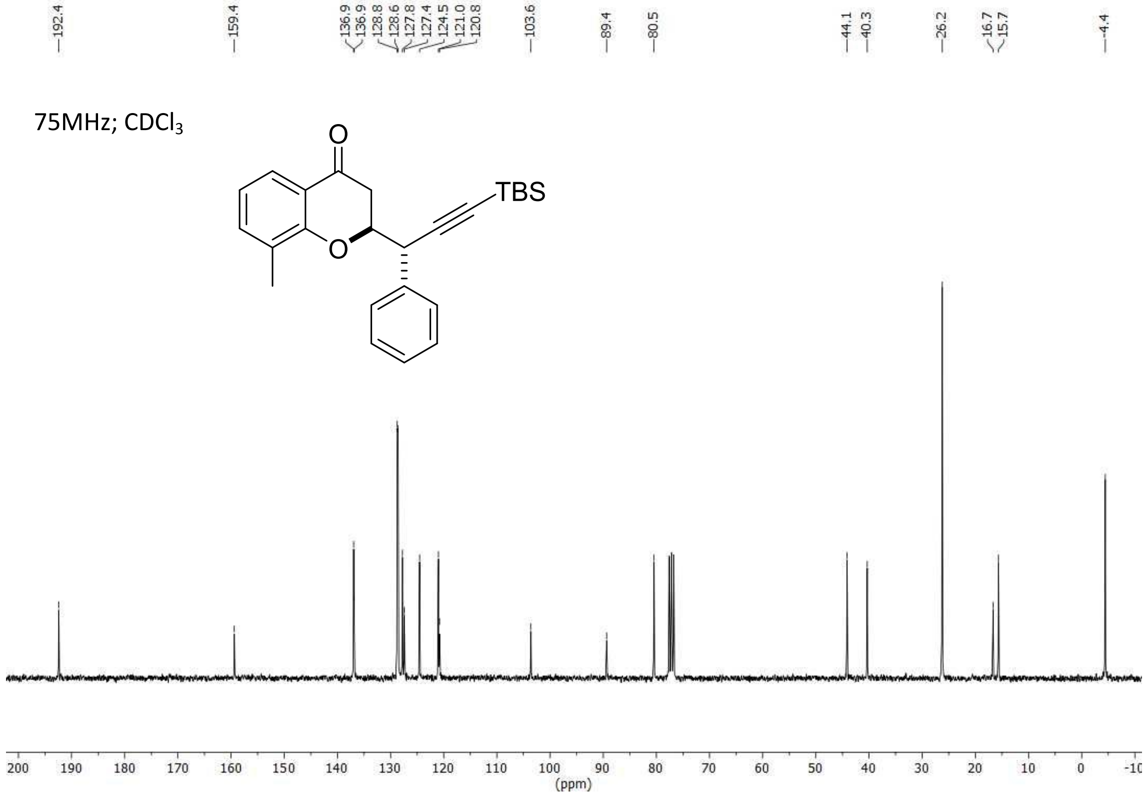

(R*)-2-[(R*)-3-(*tert*-butyldimethylsilyl)-1-phenylprop-2-yn-1-yl]-2-methylchroman-4-one (4j)

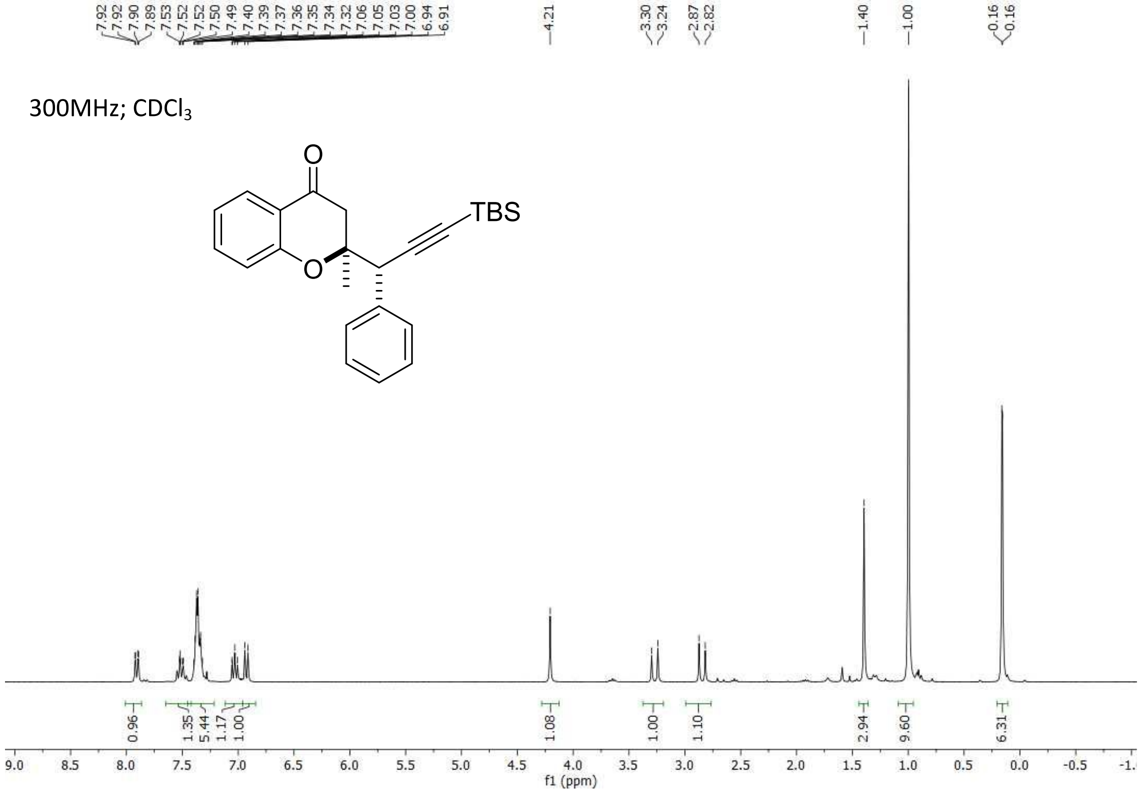

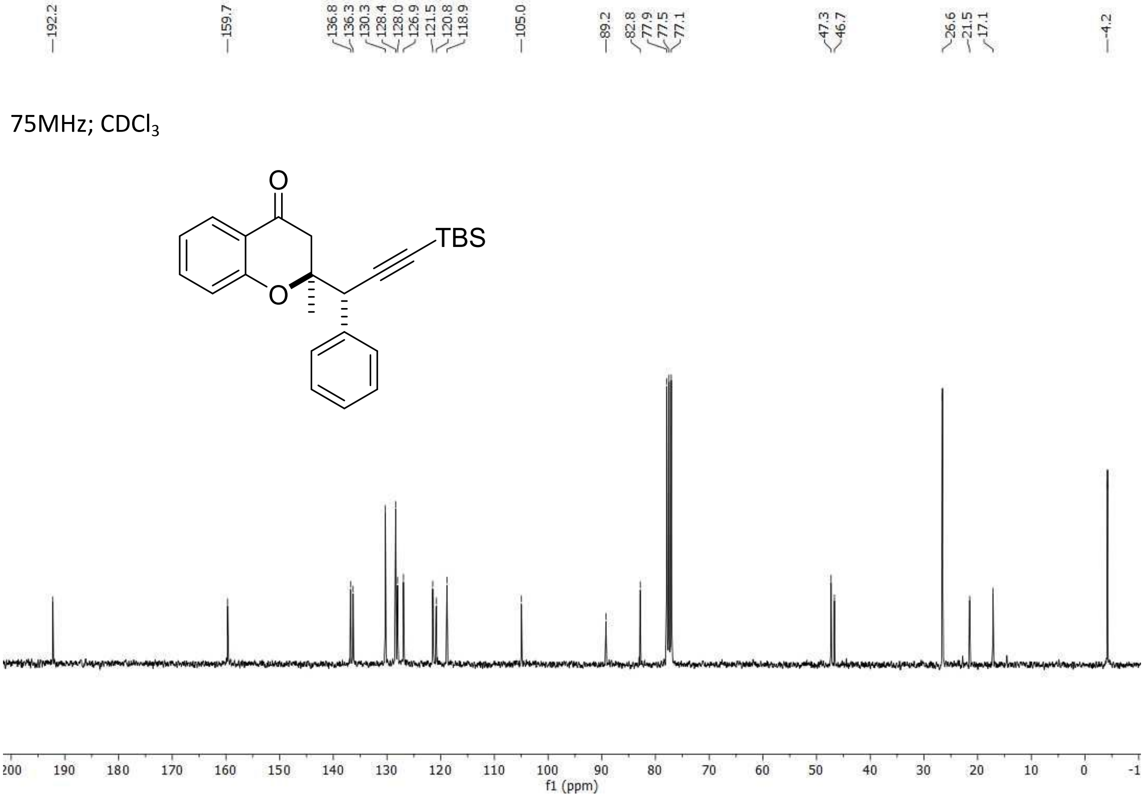

(R*)-2-[(R*)-3-(*tert*-butyldimethylsilyl)-1-phenylprop-2-yn-1-yl]-2-(*p*-trifluoromethylphenyl)methylchroman-4-one (4k)

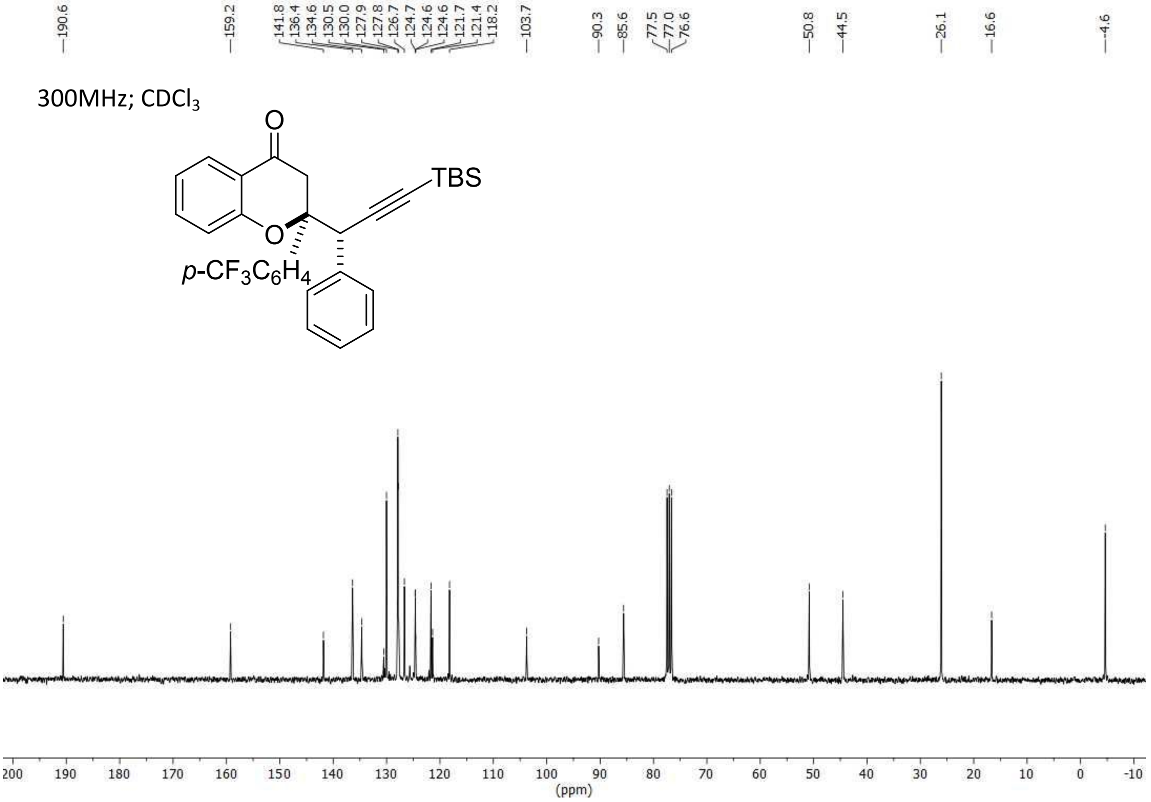

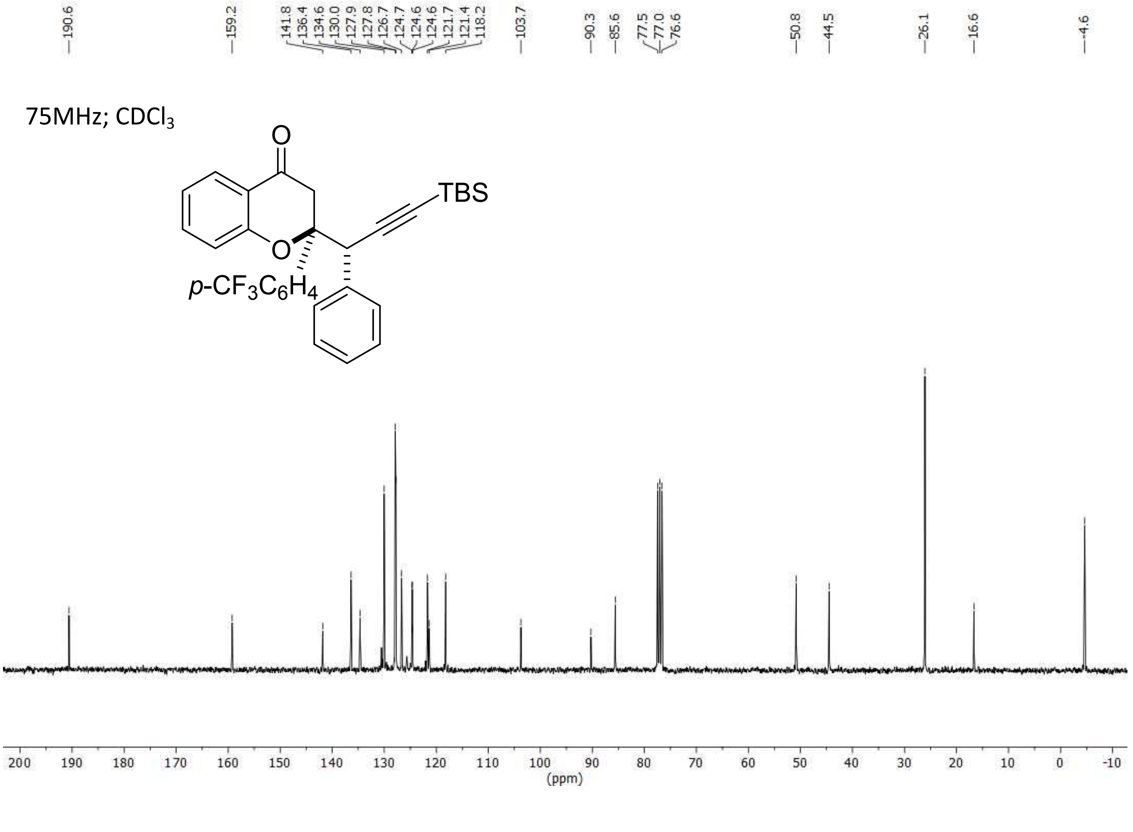

(R*)-2-[(R*)-1-phenyl-3-(triisopropylsilyl)-1-prop-2-yn-1-yl]chroman-4-one (4l)

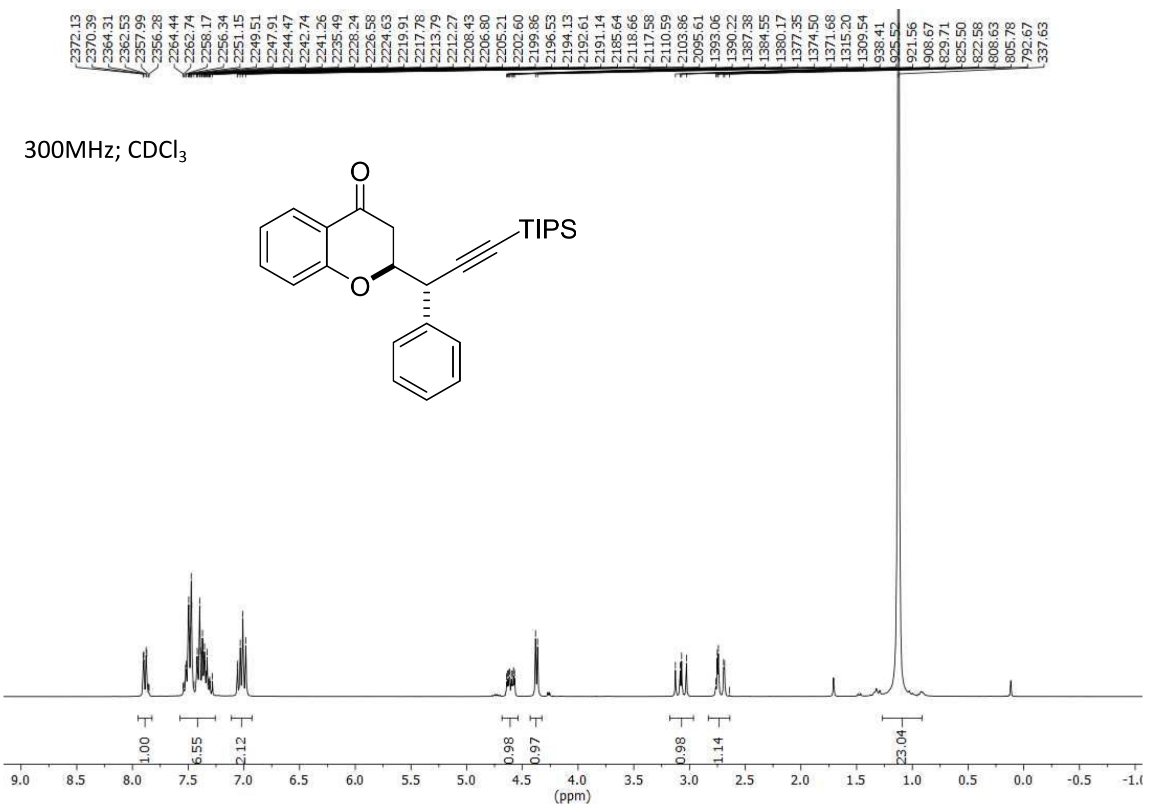

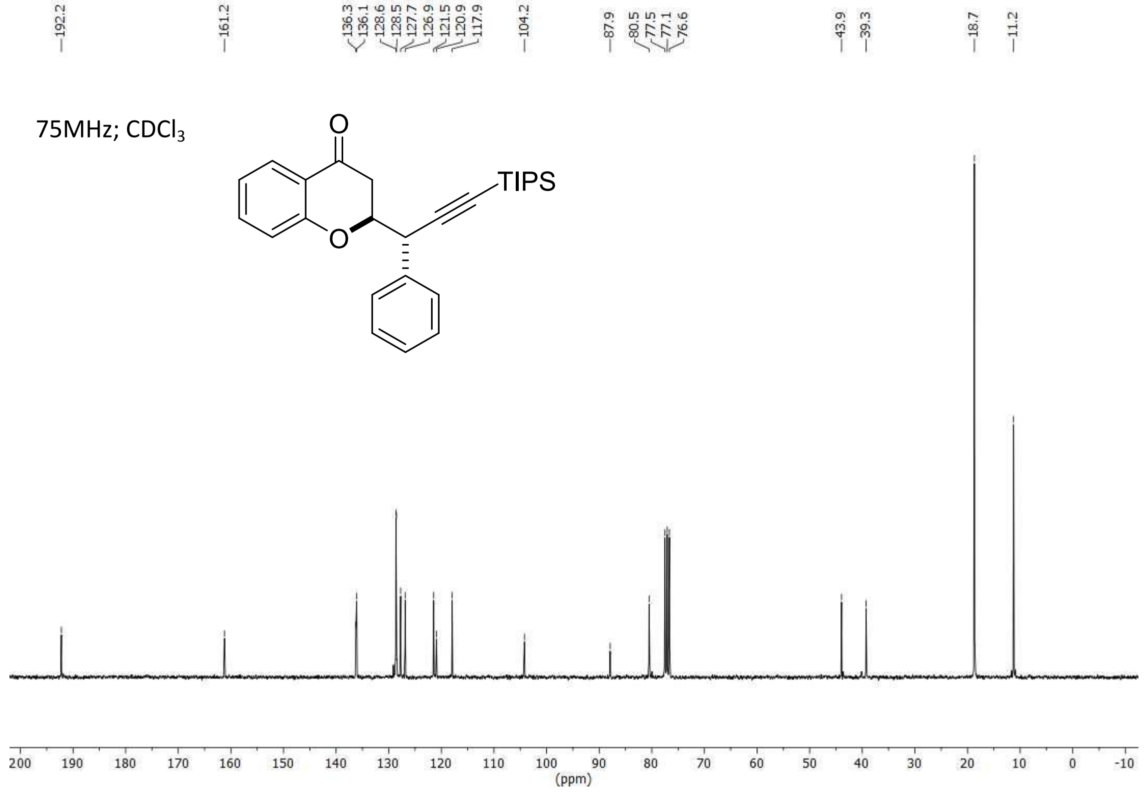

cis,cis-1-(_tert_-butyldimethylsilyl)-3-phenyl-3a,9a-dihydrocyclopenta[_b_]chromen-9(3_H_)-one (5a)

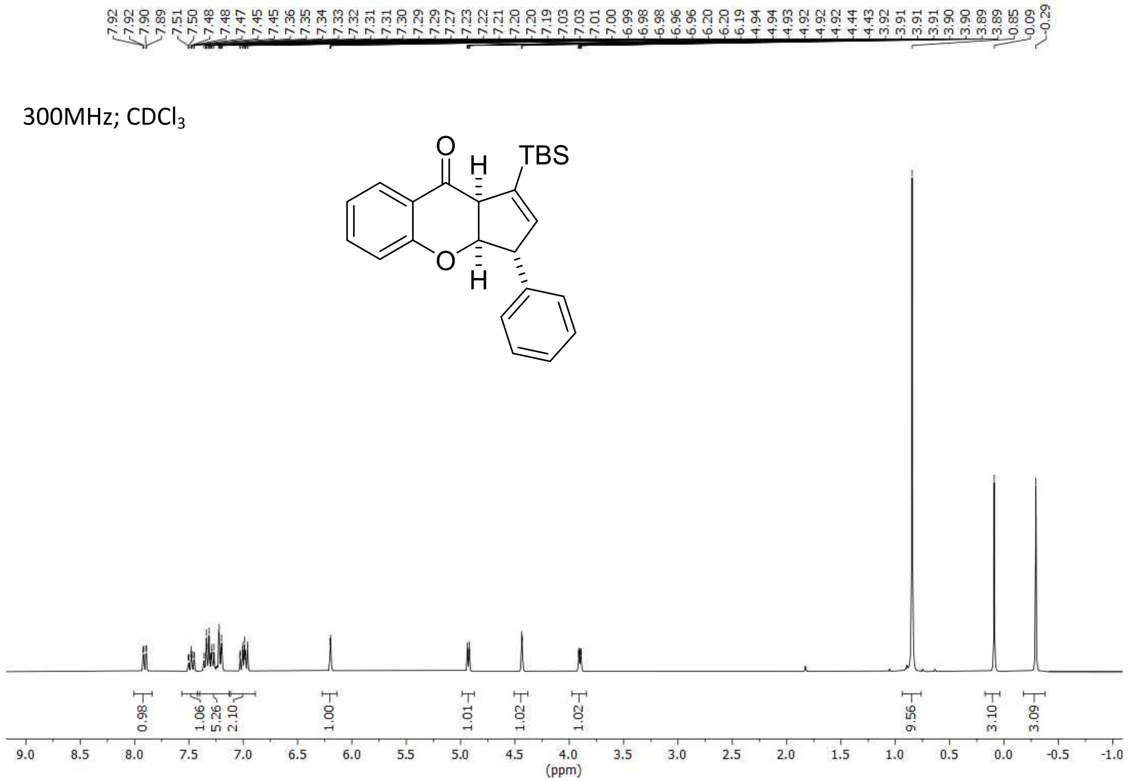

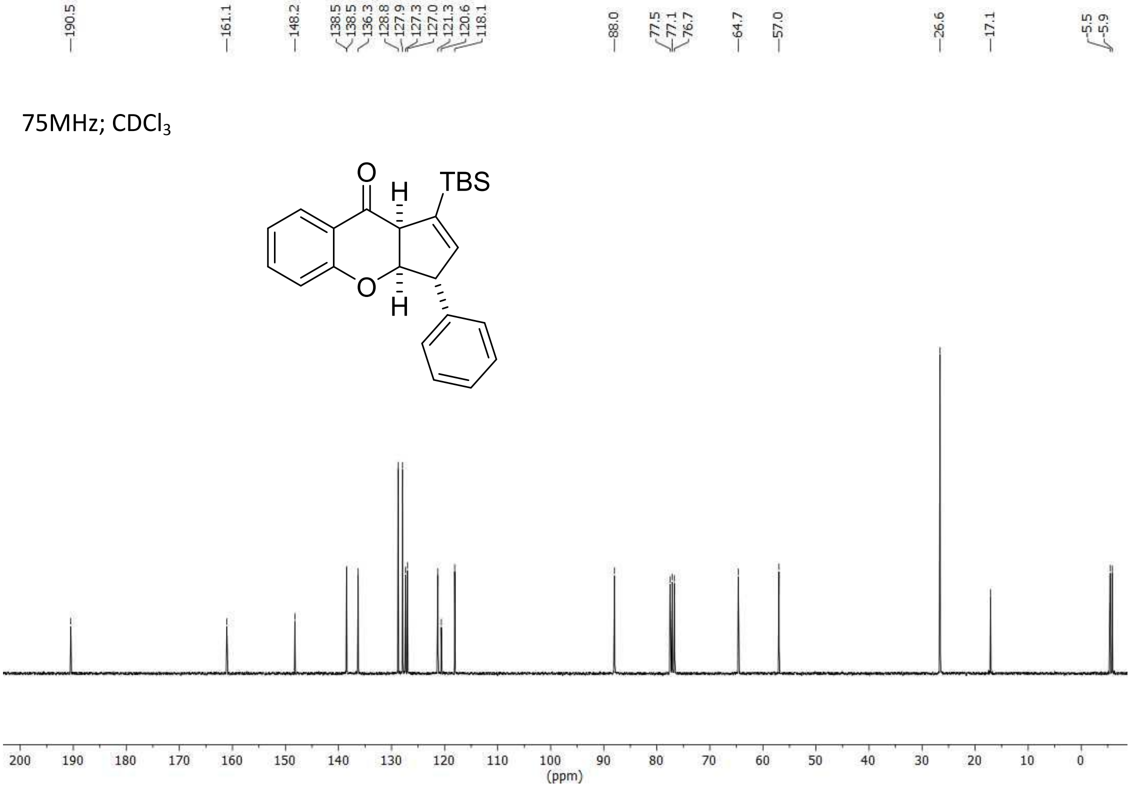

cis,cis-7-bromo-1-(_tert_-butyldimethylsilyl)-3-phenyl-3a,9a-dihydrocyclopenta[_b_]chromen-9(3_H_)-one (5b)

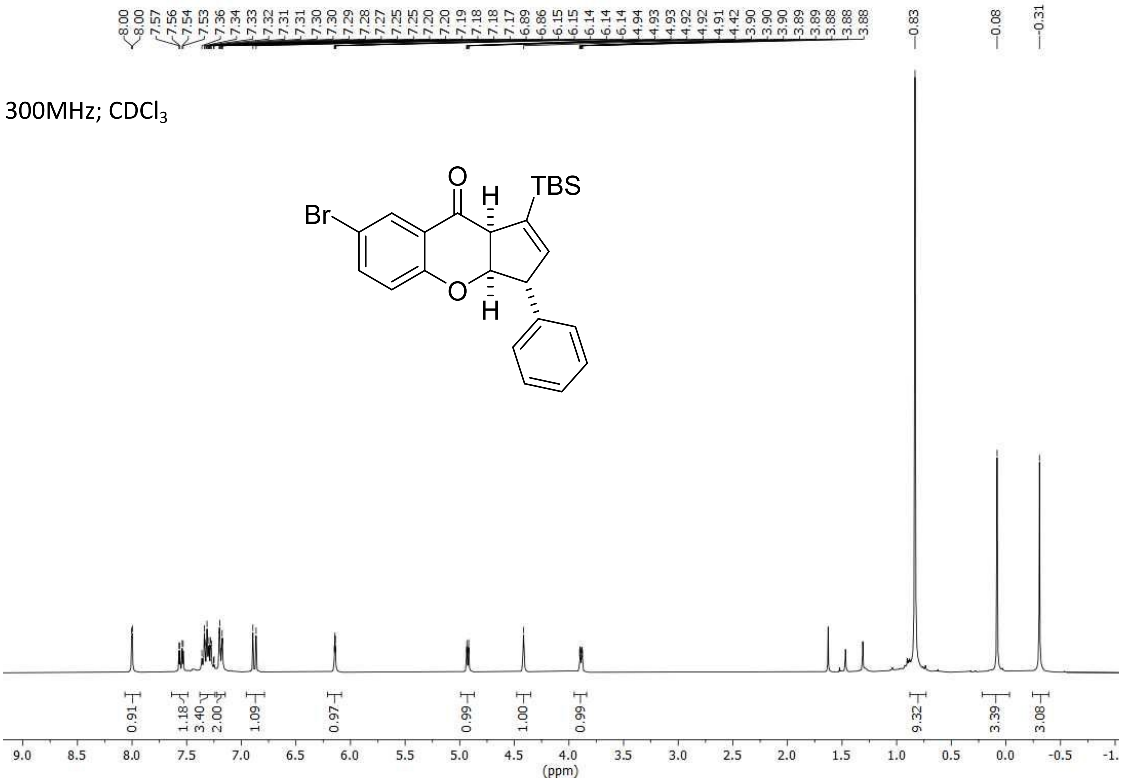

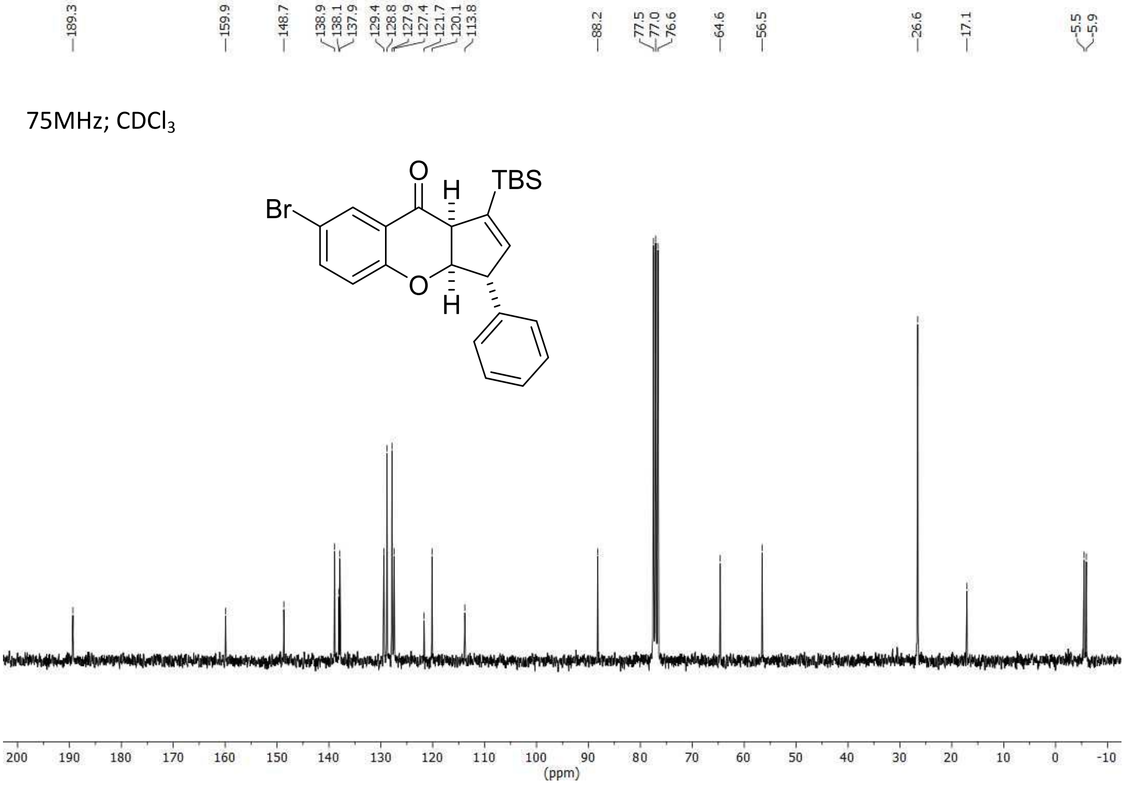

***cis,cis*-1-(*tert*-butyldimethylsilyl)-5-methyl-3-phenyl-3a,9a-dihydrocyclopenta[*b*]chromen-9(3*H*)-one (5c)**

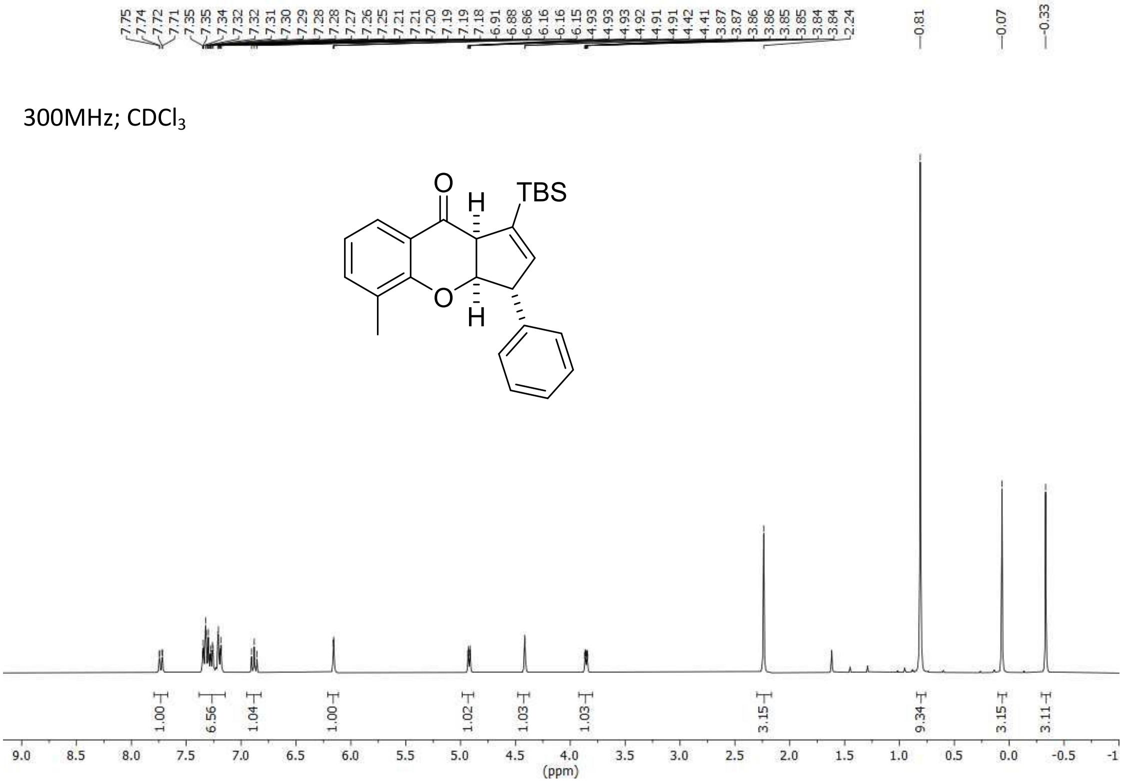

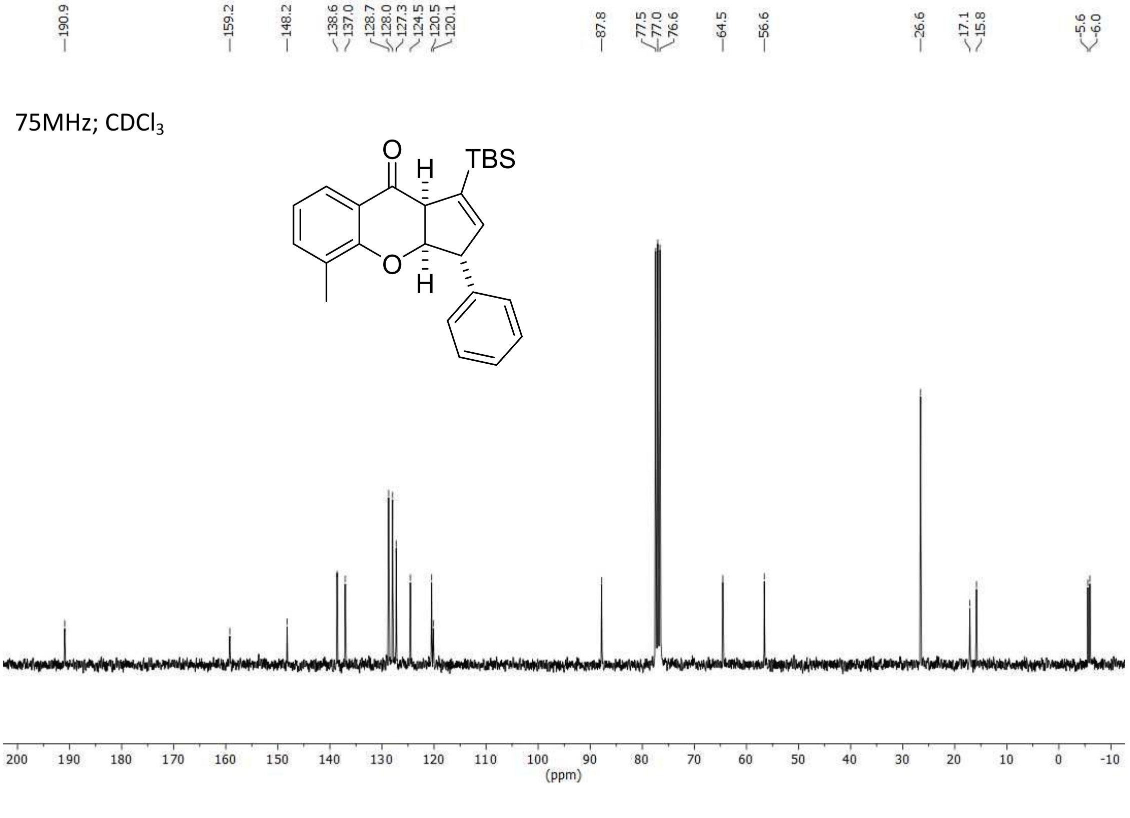

***cis,cis*-1-(*tert*-butyldimethylsilyl)-9-oxo-3-phenyl-3,3a,9,9a-tetrahydrocyclopenta[*b*]chromen-6-yl benzoate (5d)**

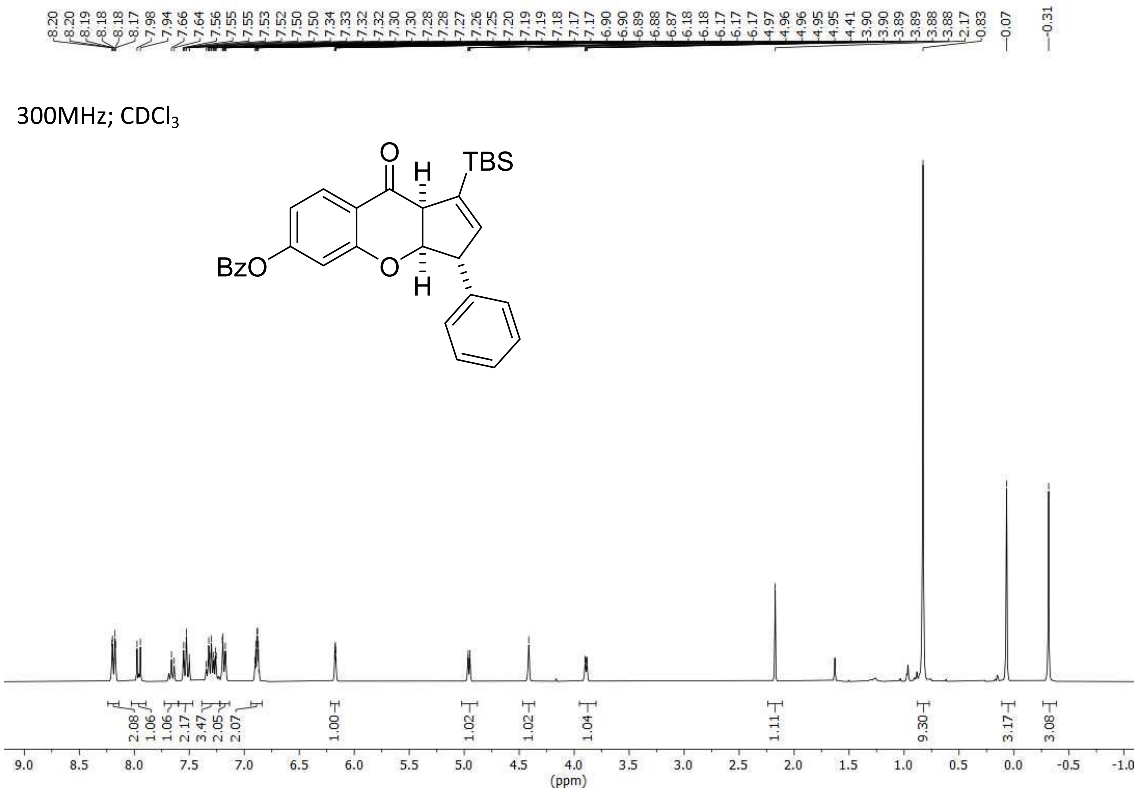

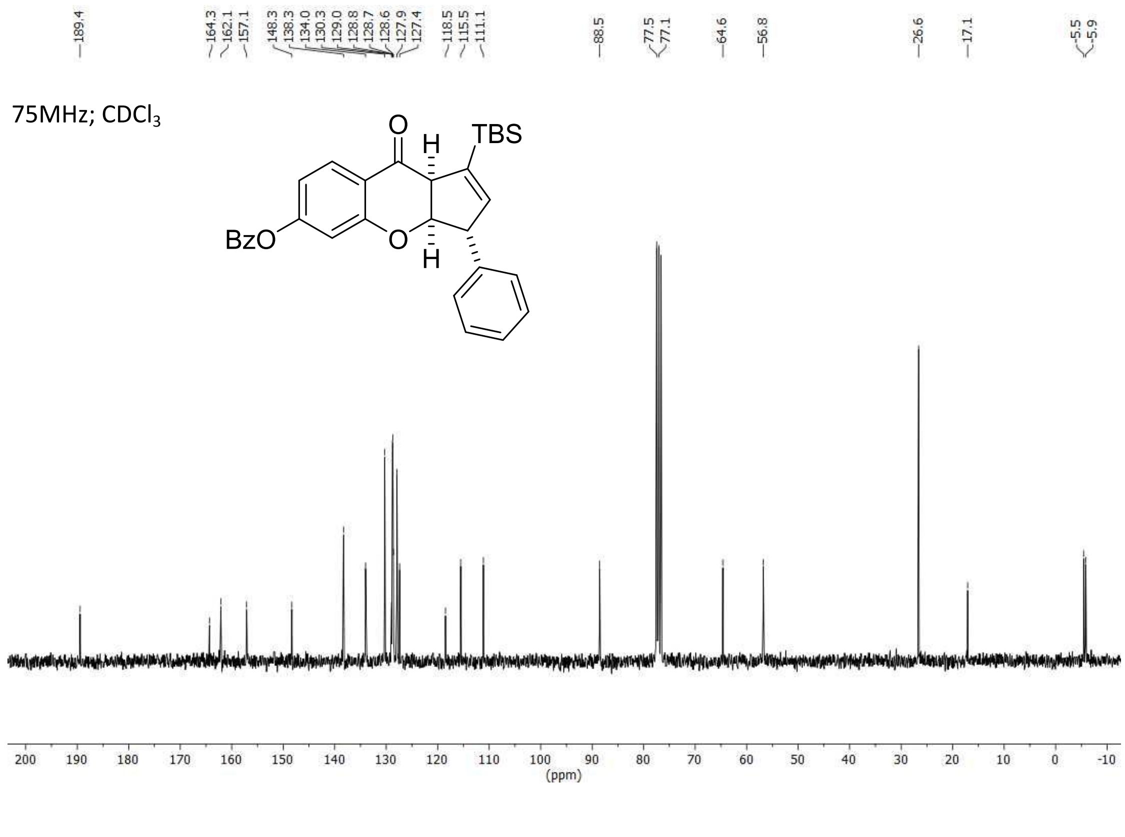

cis,cis-1-(_tert_-butyldimethylsilyl)-3-(_p_-tolyl)-3a,9a-dihydrocyclopenta[_b_]chromen-9(3_H_)-one (5e)

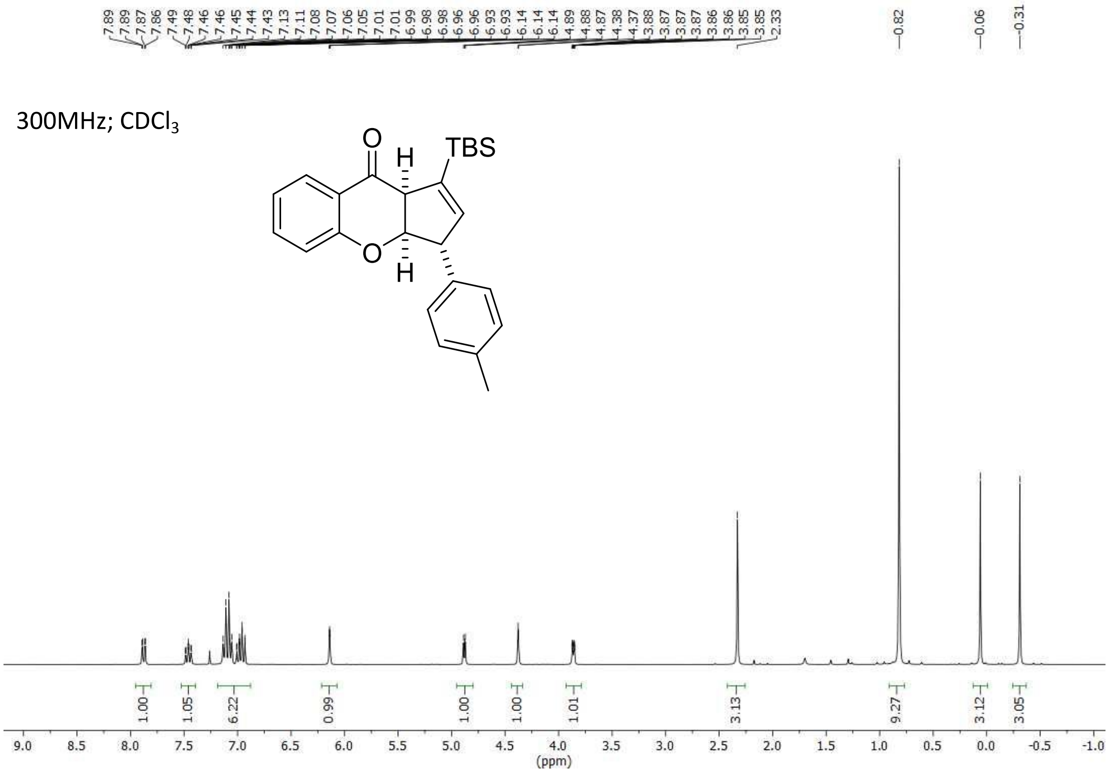

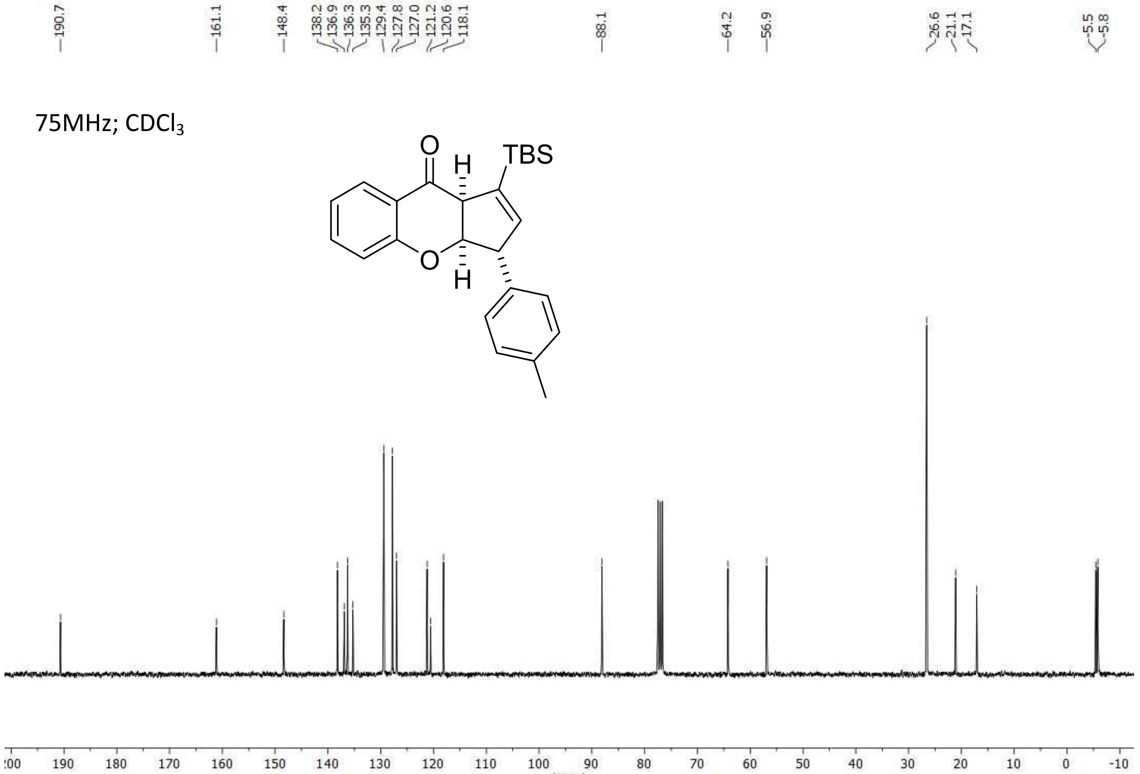

5. X-Ray diffraction analysis data.

Crystallographic data for compound 4f ($C_{24}H_{28}O_3Si$).

(CCDC-2389107) (Crystal obtained from partial evaporation of a methanol solution)

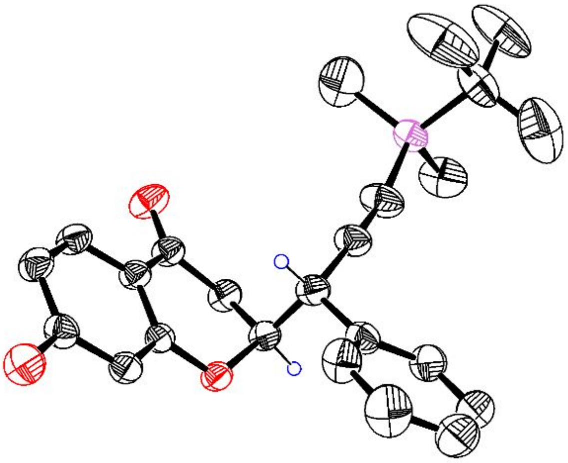

**Empirical formula:** $C_{24}H_{28}O_3Si$		**Formula weight:**	424.59
**Temperature:** 298 K		**Wavelength:**	1.54184 Å
**Crystal system / space group:**			Triclinic / P-1
**Unit cell dimensions:**	a = 6.8966 Å		α = 97.990 deg.
	b = 10.4960 Å		β = 95.845 deg.
	c = 17.0156 Å		γ = 91.659 deg.
**Volume:**	1212.24 (12) A^3	**Z / Calculated density:**	2 / 1.163 Mg/m^3
**F(000):**	456	**Absorption coefficient:**	1.065 mm-1
**Crystal size:**			0.506 x 0.182 x 0.122 mm
**Theta range for data collection:**			4.258 to 69.604 deg.
**Limiting indices:**			-8<=h<=6, -12<=k<=12, -20<=l<=20
**Reflections collected / unique:**			10282/4476 [R(int) = 0.0267]
**Completeness to theta =** 67.684			99.5 %
**Absorption correction:**			Empirical using spherical harmonics
**Max. and min. Transmission:**			1.000 and 0.523
**Refinement method:**			Full-matrix least-squares on F2
**Data / restraints / parameters:**			4476 / 0 / 335
**Goodness-of-fit on F2:**			1.020
**Final R indices [I>2σ(I)]:**			R1 = 0.0515, wR2 = 0.1507
**R indices (all data):**			R1 = 0.0600, wR2 = 0.1610
**Largest diff. peak and hole:**			0.286and -0.200 e.A^{-3}

6. Computational Details

All calculations were carried out with the Gaussian16 program package,[4] All the optimizations were performed via DFT calculations using the M06 functional,[5] the SDD basis set and the associated pseudopotential[6] for Au and the 6-31+G(d)[7] basis set for the remaining atoms. Frequency calculations were carried out at standard conditions and all the stationary points were characterized as minima (no imaginary frequencies) or transition states (1 imaginary frequency). Additionally, transition states were further characterized via relaxation to reactants and products and through IRC calculations when needed. Potential energies were further refined using a larger basis set to reduce the BSSE. In this case, single points were calculated using the same functional and basis set and pseudopotential for Au (SDD) and the larger 6-311++G(3d,2p)[8] basis set for the other atoms. Solvation was included in geometry optimizations, frequencies and single point calculations using diethyl ether as solvent and the SMD implicit solvent model.[9]

---

[4] M. J. Frisch, G. W. Trucks, H. B. Schlegel, G. E. Scuseria, M. A. Robb, J. R. Cheeseman, G. Scalmani, V. Barone, G. A. Petersson, H. Nakatsuji, X. Li, M. Caricato, A. V. Marenich, J. Bloino, B. G. Janesko, R. Gomperts, B. Mennucci, H. P. Hratchian, J. V. Ortiz, A. F. Izmaylov, J. L. Sonnenberg, Williams, F. Ding, F. Lipparini, F. Egidi, J. Goings, B. Peng, A. Petrone, T. Henderson, D. Ranasinghe, V. G. Zakrzewski, J. Gao, N. Rega, G. Zheng, W. Liang, M. Hada, M. Ehara, K. Toyota, R. Fukuda, J. Hasegawa, M. Ishida, T. Nakajima, Y. Honda, O. Kitao, H. Nakai, T. Vreven, K. Throssell, J. A. Montgomery Jr., J. E. Peralta, F. Ogliaro, M. J. Bearpark, J. J. Heyd, E. N. Brothers, K. N. Kudin, V. N. Staroverov, T. A. Keith, R. Kobayashi, J. Normand, K. Raghavachari, A. P. Rendell, J. C. Burant, S. S. Iyengar, J. Tomasi, M. Cossi, J. M. Millam, M. Klene, C. Adamo, R. Cammi, J. W. Ochterski, R. L. Martin, K. Morokuma, O. Farkas, J. B. Foresman, D. J. Fox, 2016, Gaussian 16, Revision C.01, Gaussian, Inc., Wallin.
[5] Y. Zhao, D. G. Truhlar, *Theor. Chem. Acc.* **2008**, *120*, 215.
[6] P. Fuentealba, H. Stoll, L. von Szentpaly, P. Schwerdtfeger, H. Preuss *J. Phys. B: At. Mol. Phys*. **1983**, *16*, L323-L328.
[7] R. Ditchfield, W. J. Hehre, J. A. Pople, *J. Chem. Phys.* **1971**, *54*, 724-728.
[8] P. C. Hariharan, J. A. Pople, Theor. Chim. Acta 1973, 28, 213.
[9] A. V Marenich, C. J. Cramer, D. G. Truhlar, *J. Phys. Chem. B* **2009**, *113*, 6378–6396.

3D structures of adducts III and IV

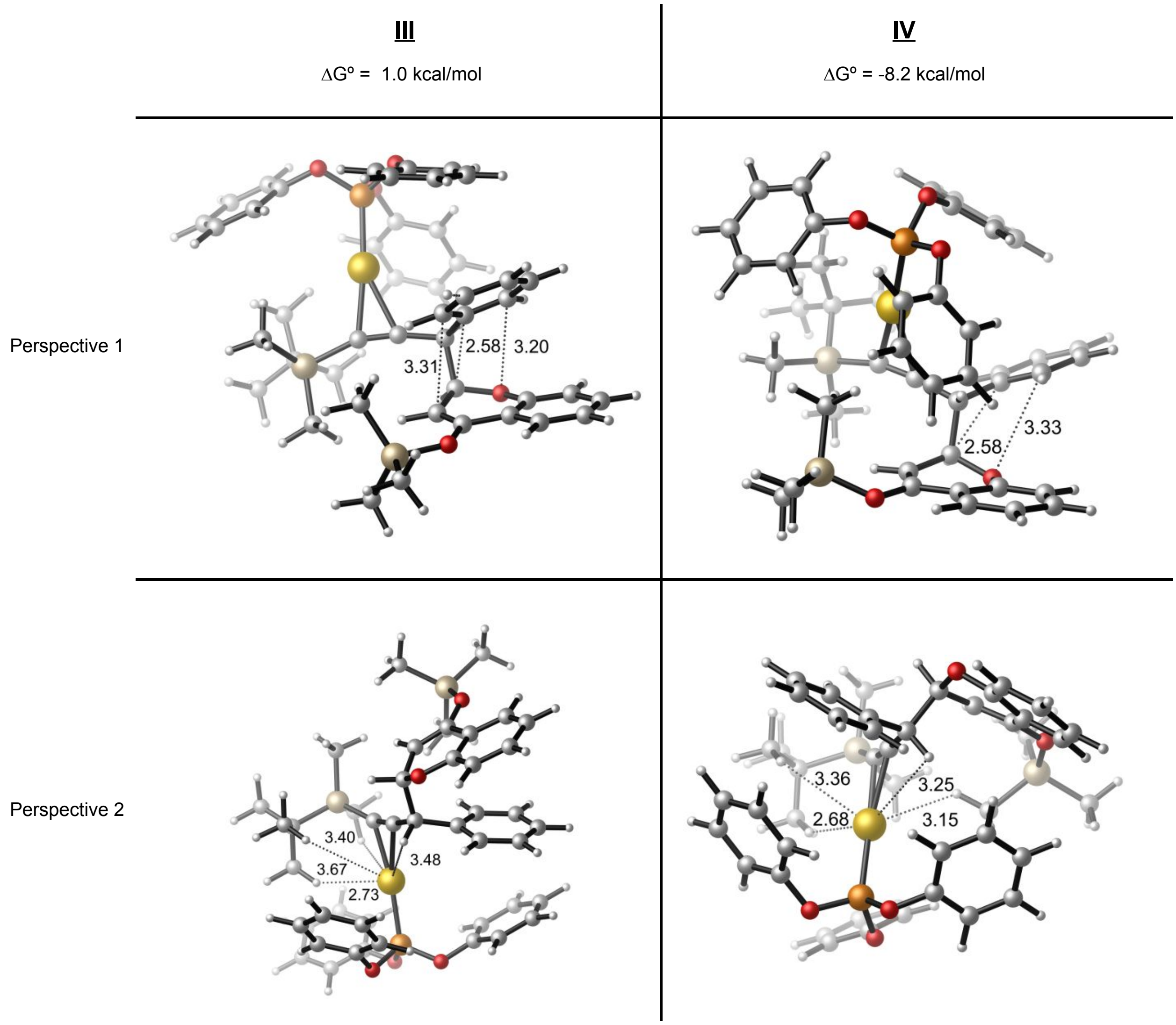

Figure S1. 3D structures of adducts **III** (left) and **IV** (right) and the corresponding Gibbs free energy. Distances in angstroms. Perspective 1: steric hindrance of phenyl group with the chromone. Perspective 2: non covalent interactions between the gold center and hydrogen atoms of the TMS and TBS groups.

Energies and Cartesian coordinates

Final free energy is the sum of E (6-31+G(d)/6-311++G(3d,2p)) + G_{corr} (6-31+G(d)). [Au] = $(PhO)_3PAu$

I

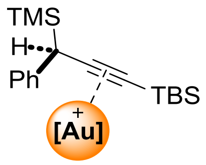

E= -2680.013608

G_{Corr}= 0.632897

1 1

Au	2.59240000	3.55144000	4.21455600
P	0.34359000	4.08416500	4.28079500
O	-0.36636500	3.83103200	5.70486700
O	-0.59883400	3.26751000	3.26582500
C	0.05460100	2.69766900	6.41625400
C	-0.62890100	1.50205200	6.24113800
C	-0.20189800	0.38136100	6.95181500
H	-0.72921100	-0.56364000	6.83180100
C	0.89210600	0.46956300	7.81101300
C	1.56002100	1.68317200	7.97240600
H	2.40996500	1.75488300	8.65008600
C	1.14259500	2.81352600	7.27327100
H	1.64537500	3.77623400	7.38957700
C	-0.04442500	2.72896300	2.09891500
C	0.35746400	3.56905200	1.06780200
C	0.87445400	2.99643900	-0.09375800
H	1.18748600	3.63985900	-0.91443400
C	0.98107800	1.61168000	-0.20783800
C	0.57335700	0.78989700	0.84247600
H	0.65787800	-0.29246800	0.75585400
C	0.05866900	1.34683800	2.01073300
H	-0.27062700	0.72621500	2.84358900
O	-0.08908600	5.59960100	3.96924100
C	0.65132800	6.64646300	4.53596200
C	0.24550900	7.17524000	5.75364400
C	1.73117200	7.15986900	3.82702300
C	0.94043800	8.26826400	6.26798600
H	-0.61175200	6.74918900	6.27217400
C	2.41753900	8.25085100	4.35621000
H	2.00652100	6.72538600	2.86575400
C	2.02102000	8.80712000	5.57202900
H	0.62849600	8.70211700	7.21677800
H	3.25762100	8.67481100	3.80747900
H	2.55452200	9.66618300	5.97668300
H	-1.48524100	1.46118300	5.56919300
H	1.22136700	-0.40944100	8.36285600
H	1.38356600	1.17061500	-1.11834200
H	0.24202700	4.64860200	1.16426900
C	4.66148100	2.72768600	4.12445800
C	4.97298200	3.79440400	4.68632100
C	5.36215400	4.98223900	5.39409300
H	5.10576800	5.86714800	4.78447000
C	4.72306500	5.09781400	6.75892700
C	4.16074500	6.31322900	7.15885700
C	4.70519700	4.01946300	7.65091400
C	3.60004600	6.45364200	8.42676900
H	4.15607800	7.15744500	6.46574500
C	4.15257500	4.16296200	8.92120900
H	5.12592300	3.05728200	7.34947500
C	3.59904500	5.38039100	9.31541500
H	3.16363800	7.40822900	8.72000100
H	4.15814600	3.31813000	9.60981100
H	3.16726100	5.49032100	10.30919700
C	6.28652100	0.16137300	3.78285700
C	4.27184000	1.15598400	1.58421800
H	5.13079900	1.57867200	1.04450000
H	4.03412300	0.18109600	1.13422500
H	3.41006900	1.81859400	1.41295100
C	3.19847900	0.11880900	4.30333100
H	3.24761900	0.28024100	5.39103600
H	2.22632600	0.49908300	3.95235400
H	3.21224600	-0.96598100	4.12142200
C	7.43419800	1.06036300	3.32627800
H	7.46817800	2.00476400	3.88915800
H	8.40002600	0.55157000	3.48397800
H	7.36963300	1.30999300	2.25567600
C	6.34832900	-1.16097600	3.01202700
H	6.34128500	-1.00362100	1.92301200
H	7.28076300	-1.69618000	3.25771100
H	5.51464600	-1.83543500	3.26212200
C	6.40568000	-0.11544900	5.28210700
H	6.25061400	0.78890300	5.89106300
H	5.67762000	-0.86863800	5.61848100
H	7.41058000	-0.50294700	5.52012500
Si	4.61249800	0.97607700	3.41572300
Si	7.31416800	5.09353500	5.48684300
C	7.90885500	5.12140400	3.71061200
H	9.00732800	5.13148500	3.66765000
H	7.56273300	4.24705000	3.14037800
H	7.55013300	6.02283400	3.19266300
C	7.99230200	3.64010400	6.45000600
H	9.09177800	3.66757700	6.42716600
H	7.68575200	3.67799200	7.50460300
H	7.68275700	2.66954600	6.03667500
C	7.67919000	6.70881700	6.35631600
H	8.76169100	6.90198400	6.35189300
H	7.19009400	7.55926200	5.85959000
H	7.34966100	6.68927400	7.40448800

II

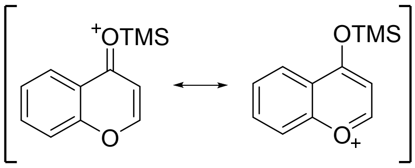

E= -905.905152

G_{Corr}= 0.198862

1 1

C	0.98850800	-2.58587600	0.18327400
C	2.36651100	-2.68478800	0.13877000
C	3.10435900	-1.51216500	0.01231300
C	2.49341300	-0.24839700	-0.06919100
C	1.08660500	-0.18009500	-0.01940100
C	0.34777500	-1.33603800	0.10421700
H	0.39262800	-3.49098900	0.28061800
H	2.88151000	-3.64100700	0.19776400
H	0.60326900	0.79183300	-0.07993200
H	-0.73794000	-1.28514800	0.14177800
O	4.45959600	-1.63657200	-0.03079500
C	3.33078200	0.90747400	-0.19723400
C	4.72781800	0.70548800	-0.23723100
H	5.43066100	1.52738000	-0.33600500
C	5.21950300	-0.56214600	-0.15151900
H	6.27870400	-0.80380500	-0.17485900
O	2.76474600	2.06556100	-0.26892300
Si	3.41075200	3.70334400	-0.41201800
C	1.85012900	4.69641200	-0.49924900
H	2.08288000	5.76864000	-0.55658700
H	1.25709600	4.43574800	-1.38564900
H	1.22608300	4.53772800	0.38997100
C	4.39733900	3.99949300	1.13525600
H	3.80477900	3.77026800	2.03128800
H	5.32250400	3.40960600	1.17430300
H	4.68329200	5.05924000	1.19392000
C	4.40431500	3.74093200	-1.98483300
H	3.89213000	3.20086500	-2.79310100
H	4.52493600	4.78190100	-2.31648500
H	5.41092200	3.31809500	-1.87108800

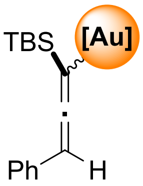

E= -2270.936107

G_{Corr}= 0.518991

0 1

Au	2.27835000	3.23249000	4.20130100
P	0.02648800	3.68674300	4.60354700
O	-0.63406700	3.14260800	5.98000000
O	-1.09595800	3.20338800	3.53678300
C	-0.16091500	1.93688500	6.50017200
C	-0.49785200	0.73491600	5.88870800
C	-0.02947900	-0.45412700	6.44674000
H	-0.28713100	-1.40324500	5.97889200
C	0.75532400	-0.42988400	7.59798300
C	1.08241600	0.78805100	8.19311800
H	1.70437900	0.81073300	9.08703500
C	0.63023100	1.98360700	7.64119900
H	0.88483600	2.94773800	8.08018800
C	-0.74009400	3.24766000	2.18597800
C	-1.05702300	4.37512800	1.43957800
C	-0.71821100	4.39840000	0.08768600
H	-0.96354900	5.27403600	-0.51148500
C	-0.06897600	3.31083700	-0.49402300
C	0.24293900	2.19019800	0.27494100
H	0.75189900	1.33977600	-0.17648400
C	-0.09288000	2.15188800	1.62581300
H	0.14037500	1.28571400	2.24583100
O	-0.30715500	5.27235600	4.70137100
C	0.74642900	6.07689500	5.15457400
C	0.93621100	6.24458600	6.52033700
C	1.59476700	6.65298300	4.21645200
C	2.01755600	7.00986800	6.95524600
H	0.24535000	5.78124700	7.22397800
C	2.66983300	7.41621100	4.66532400
H	1.41839100	6.48376300	3.15403100
C	2.88501100	7.59061800	6.03190300
H	2.18704100	7.14474100	8.02290700
H	3.35393200	7.86079800	3.94346800
H	3.73610500	8.17655200	6.37711900
H	-1.13200500	0.73270700	5.00267900
H	1.11754300	-1.36146600	8.03029300
H	0.19486600	3.33608800	-1.55014900
H	-1.56550900	5.21239300	1.91557700
C	4.32542700	2.97721400	3.90257600
C	5.02422000	4.06644600	3.92734700
C	5.72264100	5.19935100	4.00491400
H	5.99135600	5.72193800	3.08211400
C	6.11418900	5.83385600	5.26215400
C	6.95806500	6.95739100	5.24775800
C	5.64036700	5.38649100	6.50911400
C	7.32712200	7.59684500	6.42787900
H	7.32901400	7.32681400	4.28979500
C	6.01433100	6.02243400	7.68724100
H	4.95438100	4.53709100	6.54457600
C	6.86240700	7.13152200	7.65808700
H	7.98623900	8.46435000	6.38618400
H	5.63170900	5.65411600	8.63980500
H	7.15330900	7.62901900	8.58273500
C	5.35415200	0.50179600	5.44902800
C	6.62184500	1.32330900	2.72147000
H	7.33464300	2.04080600	3.15279300
H	7.11940400	0.34399000	2.66055700
H	6.40698300	1.65031900	1.69342400
C	3.76579300	0.20290900	2.78476100
H	2.87656400	-0.01657200	3.39625300
H	3.42314600	0.74141300	1.88784700
H	4.19589100	-0.75484700	2.45591900
C	6.59492000	1.15517300	6.05839800
H	6.50811900	2.25264500	6.10711700
H	6.75373500	0.79329200	7.08957500
H	7.50580600	0.91990800	5.48744000
C	5.59207400	-1.00373200	5.31399200
H	6.41722800	-1.23867800	4.62294700
H	5.85738500	-1.43946300	6.29342300
H	4.69531600	-1.53189500	4.95586600
C	4.15779700	0.73443400	6.37140100
H	3.99171400	1.80458300	6.56979300
H	3.22068200	0.32635600	5.95774400
H	4.32418000	0.23990100	7.34568100
Si	5.02733600	1.24680300	3.71926700

III

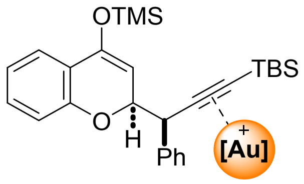

E= -3176.875409

G_{Corr}= 0.757926

1 1

Au	0.06292800	2.17444100	-1.77993900
P	2.00150400	3.32489900	-1.22895800
O	2.38154900	4.67095600	-2.02151900
O	3.31320200	2.40899700	-1.43799500
C	2.22167600	4.68654900	-3.41472800
C	3.25948400	4.24187900	-4.22344800
C	3.09921500	4.31015300	-5.60612300
H	3.90503600	3.97261600	-6.25602300
C	1.92249000	4.81793500	-6.15513800
C	0.89501600	5.25794000	-5.32206000
H	-0.02316000	5.65952200	-5.74899500
C	1.03911300	5.19345400	-3.93773900
H	0.25560600	5.54512200	-3.26580600
C	3.11474900	1.02247200	-1.46146500
C	2.74760900	0.35511400	-0.29884100
C	2.51616800	-1.01845900	-0.36383200
H	2.23159500	-1.55740300	0.53911700
C	2.65246100	-1.69787900	-1.57323800
C	3.03325900	-1.00698600	-2.72382600
H	3.14478300	-1.53611600	-3.66929200
C	3.26378900	0.36574500	-2.67534500
H	3.54867000	0.92905500	-3.56362800
O	2.16741200	3.80305200	0.29224100
C	1.06831000	4.23581600	1.04042700
C	0.22690500	5.23453300	0.56597000
C	0.88747900	3.64796000	2.28533200
C	-0.83282200	5.65109500	1.37173900
H	0.41165800	5.70512200	-0.39951500
C	-0.16634500	4.08464000	3.08212400
H	1.57310700	2.87179800	2.62207700
C	-1.02788500	5.08239700	2.62737100
H	-1.49830300	6.43584600	1.01528100
H	-0.31532600	3.63760400	4.06379800
H	-1.85341900	5.41555900	3.25503500
H	4.17714700	3.86703400	-3.77308500
H	1.80748900	4.87439600	-7.23629200
H	2.47066300	-2.77050400	-1.61778200
H	2.65447400	0.90129600	0.64014000
C	-1.67713700	1.02600000	-2.59023300
C	-1.58283300	0.51809500	-1.46096500
C	-1.66505200	-0.24147200	-0.22172500
H	-0.70205100	-0.75148900	-0.05389600
C	-2.01050300	0.58937200	0.99094400
C	-2.87495900	1.68537900	0.90775000
C	-1.54036200	0.18621000	2.24294900
C	-3.30507600	2.33030700	2.06411800
H	-3.22983300	2.02690800	-0.06636400
C	-1.97283800	0.82855800	3.40034000
H	-0.85552400	-0.66164500	2.31058700
C	-2.86834900	1.89234900	3.31315000
H	-3.98704400	3.17735700	1.98822800
H	-1.61522300	0.49053000	4.37255000
H	-3.21426300	2.39163500	4.21803400
C	-0.69313500	0.42148500	-5.38030900
C	-2.36428100	3.02916500	-4.76692600
H	-1.50325600	3.62298700	-4.42936700
H	-2.49969400	3.21221100	-5.84265000
H	-3.25865900	3.40747700	-4.25087400
C	-3.73153300	0.25244400	-4.59935500
H	-3.59887200	-0.81749200	-4.38253900
H	-4.50064300	0.64806300	-3.91992500
H	-4.12127400	0.34369800	-5.62355400
C	0.54399800	1.31636100	-5.32172000
H	0.88779300	1.48684100	-4.28569200
H	1.38256600	0.84509900	-5.86381200
H	0.36596300	2.30039400	-5.78303600
C	-1.13724900	0.26994700	-6.83910900
H	-1.42985300	1.23012300	-7.29208600
H	-0.30848600	-0.13317700	-7.44446500
H	-1.98262600	-0.42625500	-6.94327000
C	-0.35985100	-0.95754100	-4.80949600
H	0.01117100	-0.90053200	-3.77430000
H	-1.22873400	-1.63412700	-4.81769500
H	0.42891000	-1.43539400	-5.41486700
Si	-2.13099300	1.20798600	-4.41659600
C	-2.71491200	-1.39634900	-0.50206700
O	-2.66960000	-2.30951500	0.58336700
C	-4.07961600	-0.88169800	-0.80353500
H	-2.31480800	-1.95309500	-1.36420700
C	-3.56916300	-2.18346200	1.60048000
C	-5.02882200	-0.84276700	0.15160400
H	-4.23888700	-0.47278300	-1.80035400
C	-3.28502300	-2.85241800	2.78599200
C	-4.75546200	-1.45059000	1.45353700
O	-6.25293300	-0.30968400	0.01820600
C	-4.17279100	-2.75988000	3.85391600
H	-2.36201800	-3.42654700	2.85980800
C	-5.63089100	-1.36489000	2.53930500
Si	-6.89577500	0.57882400	-1.28592500
C	-5.34299200	-2.00869400	3.73794100
H	-3.94490600	-3.27727700	4.78511200
H	-6.54875600	-0.79081300	2.42523800
C	-7.06066800	-0.53423900	-2.77966200
C	-8.56441100	1.11708700	-0.66039900
C	-5.80144500	2.06278400	-1.62303200
H	-6.03287200	-1.93339300	4.57674700
H	-7.34849000	0.05487900	-3.66329200
H	-6.13771800	-1.07599100	-3.02617400
H	-7.84802900	-1.28319900	-2.61342000
H	-9.18465900	0.25214300	-0.38823700
H	-8.47297800	1.76107000	0.22489200
H	-9.10316300	1.68358200	-1.43333700
H	-4.77116600	1.79481700	-1.89819800
H	-6.22424900	2.65261000	-2.45085200
H	-5.75832400	2.71915700	-0.7412390

IV

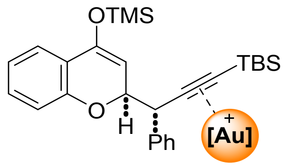

E= -3176.885247

GCorr= 0.753096

1 1

Au	6.72981600	4.17890800	3.85996700
P	7.39945100	5.05193300	1.81350600
O	8.82481000	4.58480900	1.23164700
O	7.49008300	6.65836300	1.76540700
C	9.84231000	4.29996200	2.15147100
C	10.69196900	5.31749400	2.56340100
C	11.71404100	5.00537100	3.45848100
H	12.39314400	5.78868700	3.79153200
C	11.87056200	3.69955800	3.92160800
C	11.00625200	2.69437200	3.48872200
H	11.13078600	1.67230500	3.84516800
C	9.97760600	2.99148500	2.59733900
H	9.28967000	2.22564400	2.23736200
C	6.75910000	7.35486200	2.74041900
C	5.37033200	7.35936900	2.69192000
C	4.67147200	8.01823100	3.70214600
H	3.58168300	8.02118900	3.68585200
C	5.35977900	8.66617400	4.72599300
C	6.75414800	8.66446500	4.74075300
H	7.29453300	9.17512200	5.53667200
C	7.46692400	7.99839400	3.74615900
H	8.55627000	7.97172800	3.74676100
O	6.42654600	4.73073300	0.57194100
C	5.88067900	3.43944500	0.51717500
C	6.61807000	2.40328900	-0.04065800
C	4.60014300	3.25043100	1.02259700
C	6.04666100	1.13200600	-0.08603900
H	7.61236700	2.59454600	-0.44177400
C	4.04273700	1.97497100	0.97028600
H	4.05505800	4.09949300	1.43693700
C	4.76678700	0.91639400	0.42178500
H	6.60769000	0.30796000	-0.52449400
H	3.03629400	1.80393500	1.35480200
H	4.32410800	-0.07826700	0.38176900
H	10.56131400	6.32606000	2.17331200
H	12.67400300	3.46258300	4.61731600
H	4.80675100	9.17572000	5.51391000
H	4.84647500	6.86340300	1.87450400
C	6.23713400	3.38475000	5.89730800
C	5.12988200	3.30419600	5.34263100
C	3.76029200	3.12690200	4.87492700
H	3.78920800	2.79080700	3.82298800
C	2.95627400	4.40408000	4.97487700
C	3.01461000	5.20018300	6.12157200
C	2.11442500	4.77767100	3.92584900
C	2.24081400	6.35391500	6.21705700
H	3.67130600	4.91533500	6.94719200
C	1.34018000	5.93228500	4.01911800
H	2.05516900	4.15157100	3.03272300
C	1.40228400	6.72228600	5.16559900
H	2.29188200	6.96696800	7.11621600
H	0.68474700	6.21254400	3.19533600
H	0.79690400	7.62485000	5.24027000
C	8.26976500	5.04853700	7.43909400
C	8.84961900	2.06203000	6.64593300
H	9.22246400	2.30923900	5.64161300
H	9.71174700	2.04268000	7.32894800
H	8.43365100	1.04421800	6.61265800
C	6.63055600	2.69035800	8.73172700
H	5.84302400	3.39812600	9.02881300
H	6.14944000	1.72426500	8.51860800
H	7.29987800	2.54364500	9.59099500
C	9.18708400	5.39606300	6.26702000
H	8.65943200	5.35039800	5.29630100
H	9.57317500	6.42492400	6.37294300
H	10.05568800	4.72216100	6.20593000
C	9.07785000	5.08081300	8.74036300
H	9.89116000	4.33835000	8.74963000
H	9.54366900	6.07177900	8.87005400
H	8.44583700	4.90104100	9.62270400
C	7.13120700	6.06560300	7.52723400
H	6.55383700	6.12633800	6.59063200
H	6.42700900	5.83415000	8.34159700
H	7.53834500	7.07123600	7.72669400
Si	7.57091000	3.29661800	7.23044700
C	3.14993700	1.92638200	5.68433600
O	1.80603200	1.74762900	5.26567800
C	3.98114600	0.69638000	5.51429300
H	3.08657900	2.22749100	6.74012700
C	1.60347600	1.04295800	4.11748500
C	3.72957500	-0.13295400	4.48357400
H	4.83316000	0.55325000	6.17671900
C	0.41760300	1.26571300	3.42424500
C	2.52556400	0.07754900	3.67974900
O	4.49125600	-1.18369400	4.13214000
C	0.14614700	0.52470500	2.27730500
H	-0.27810200	2.01644600	3.79758800
C	2.22766300	-0.66258400	2.53154600
Si	6.18762600	-1.29799300	4.25375200
C	1.04911600	-0.44093200	1.82776500
H	-0.78012300	0.70222300	1.73250200
H	2.93522300	-1.42307600	2.20398700
C	6.68823600	-1.73157400	6.00183200
C	6.60398900	-2.66027000	3.05428400
C	6.95818400	0.32844600	3.72380600
H	0.82917600	-1.02296900	0.93425700
H	7.76872600	-1.93680300	6.04114700
H	6.47871900	-0.92298100	6.71718800
H	6.16865900	-2.63330100	6.35458800
H	6.07836900	-3.59180300	3.30485000
H	6.32124000	-2.37909700	2.02924300
H	7.68273900	-2.87163400	3.05916400
H	6.89557400	1.10263000	4.50108500
H	8.02505600	0.16165500	3.50491700
H	6.48201000	0.71829500	2.80881400

$TS_{I\text{-}II}$

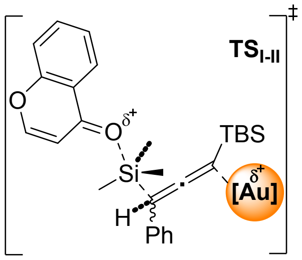

E= -3176.836303

GCorr= 0.747293

1 1

Au	4.04287100	5.07831600	3.90306000
P	1.87253700	5.82852000	3.61879500
O	0.72451900	4.69123200	3.71202500
O	1.52938400	6.53483400	2.20817200
C	1.04962500	3.44154200	3.17185700
C	0.73947500	3.17837800	1.84423900
C	1.06725200	1.93019700	1.31646200
H	0.82741800	1.70641600	0.27807700
C	1.69498300	0.97372600	2.11237800
C	1.99267000	1.25887000	3.44489400
H	2.47962300	0.51006100	4.06866200
C	1.67110500	2.50135800	3.98587700
H	1.89556600	2.74695500	5.02509000
C	2.58552200	6.99373700	1.41566300
C	3.24227700	8.17178100	1.74827300
C	4.28291000	8.60916100	0.92964400
H	4.80615100	9.53206200	1.17508600
C	4.65106200	7.87296000	-0.19523200
C	3.97836700	6.69238000	-0.50795000
H	4.26562600	6.11298300	-1.38441000
C	2.93780200	6.24305300	0.30145400
H	2.39800900	5.32353400	0.07711900
O	1.30713000	6.91889900	4.66448100
C	1.69171300	6.76541800	6.00300000
C	0.98282800	5.89660400	6.82311600
C	2.76878100	7.50866700	6.46887700
C	1.36241000	5.78439900	8.15891700
H	0.14271000	5.33233100	6.42154900
C	3.13363400	7.38909000	7.80810900
H	3.29927300	8.17422000	5.78796600
C	2.43030400	6.53131100	8.65229000
H	0.81827300	5.10694300	8.81545200
H	3.97057700	7.97171600	8.19080000
H	2.71745900	6.44097500	9.69932100
H	0.24060800	3.93863000	1.24454000
H	1.94920800	0.00085700	1.69455300
H	5.46619100	8.21888400	-0.82906300
H	2.93230000	8.73926000	2.62595200
C	6.02063900	4.25553800	4.10085600
C	6.10030300	4.28946200	5.36769500
C	6.07745800	4.27795000	6.73997200
H	6.26753200	5.24532100	7.22241900
C	5.05388500	3.43803900	7.39895400
C	4.59265100	3.76535300	8.68454800
C	4.49689900	2.30631900	6.77797000
C	3.62955600	2.99360100	9.32697500
H	4.99347900	4.65123100	9.18078200
C	3.53245000	1.53479600	7.42089100
H	4.82233000	2.02836900	5.77208200
C	3.09201500	1.86981500	8.70104300
H	3.28827100	3.28148600	10.32222500
H	3.12087300	0.66016500	6.91588100
H	2.33661300	1.26527100	9.20142200
C	8.16488800	2.64993300	2.40586000
C	7.08240200	5.46193900	1.56788700
H	7.98194600	5.92371500	2.00074500
H	7.24987400	5.33589500	0.48784400
H	6.25127000	6.17255400	1.69446100
C	5.23453300	2.98953200	1.48350700
H	4.76800500	2.21120700	2.10686900
H	4.45071500	3.71650500	1.21950100
H	5.57197900	2.51539800	0.54964000
C	9.24246300	3.15169000	3.36296500
H	8.88631800	3.17226200	4.40104000
H	10.12349000	2.48661900	3.33117900
H	9.58976800	4.16542200	3.10740600
C	8.75122000	2.59132300	0.99204700
H	9.17162200	3.55922200	0.68025100
H	9.57055100	1.85305600	0.95374000
H	8.00940600	2.28812100	0.23692400
C	7.71412900	1.24871000	2.82180500
H	7.18529900	1.24634000	3.78819700
H	7.04292000	0.79718500	2.07622900
H	8.58561700	0.57894000	2.92499100
Si	6.66218000	3.82298100	2.38511100
Si	8.40806300	3.52636000	7.35498500
C	9.02502300	5.02974000	6.39999900
H	10.08618300	4.93779300	6.13018400
H	8.46259600	5.21700600	5.47785000
H	8.91375700	5.92556800	7.03013700
C	8.07630000	1.84038400	6.61141500
H	8.93834500	1.48335000	6.03221800
H	7.92704600	1.13360000	7.44220800
H	7.18454400	1.80260300	5.97833200
C	7.89632600	3.78445600	9.14267300
H	8.68364900	3.46759700	9.83948500
H	7.65851600	4.83682800	9.35308100
H	7.00309200	3.19099200	9.37552900
O	10.20404700	2.91000000	7.79149100
C	11.17795300	3.42032600	8.41917000
C	12.39945300	2.65755200	8.56718000
C	11.17087300	4.71951500	9.00881500
C	13.47689100	3.22148600	9.26603100
C	12.55211900	1.36362200	8.03506900
C	12.27097100	5.16991700	9.66360100
H	10.30106000	5.36724000	8.94851900
C	14.67859700	2.54088400	9.44628100
O	13.39215800	4.47195400	9.80281900
C	13.73831000	0.68021300	8.20592100
H	11.71973200	0.92064400	7.49182600
H	12.33719700	6.14673000	10.13650400
C	14.80007400	1.27050800	8.91261800
H	15.48741900	3.01674500	9.99686500
H	13.85421200	-0.32017300	7.79419100
H	15.73196400	0.72378200	9.04371100

TS_{II-III}

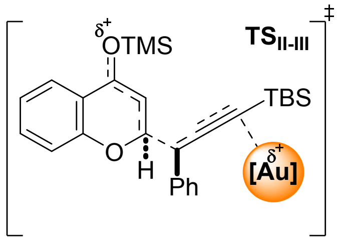

E= -3176.852648

G_{Corr}= 0.745617

1 1

Au	0.46007800	2.36550700	-1.80683900
P	2.35664200	3.61549600	-1.31471000
O	2.59554800	5.02617600	-2.06225200
O	3.75642900	2.85548000	-1.59120400
C	2.27166400	5.08858800	-3.42325900
C	3.23591000	4.75688500	-4.36681300
C	2.90427500	4.84593500	-5.71751100
H	3.64939400	4.59380300	-6.47042300
C	1.63061600	5.26122400	-6.10358900
C	0.68081800	5.59367000	-5.13846400
H	-0.31335000	5.92288700	-5.43850700
C	0.99611600	5.50538400	-3.78413400
H	0.27286500	5.76230100	-3.00922600
C	3.72377000	1.45705000	-1.63377700
C	3.57669900	0.73335600	-0.45705900
C	3.51647100	-0.65746300	-0.53206000
H	3.40222200	-1.23851300	0.38190000
C	3.61018200	-1.29978400	-1.76562700
C	3.77103500	-0.55208900	-2.93202800
H	3.84724300	-1.05139000	-3.89711800
C	3.82471100	0.83827200	-2.87281100
H	3.93768900	1.44312100	-3.77225500
O	2.51804900	4.05649800	0.22883600
C	1.33494700	4.38247900	0.90774900
C	0.82337900	5.66920600	0.80389700
C	0.70991200	3.39665700	1.66208300
C	-0.35411700	5.97506700	1.48543600
H	1.34639700	6.41582100	0.20775100
C	-0.46462700	3.71721500	2.33765700
H	1.13722700	2.39471200	1.71349200
C	-0.99831300	5.00230300	2.24748700
H	-0.76614100	6.98075600	1.41734900
H	-0.96962200	2.95430400	2.93063500
H	-1.91977400	5.24392900	2.77616200
H	4.22961500	4.44997400	-4.04369100
H	1.37932900	5.33215000	-7.16073900
H	3.56517000	-2.38647200	-1.81782300
H	3.52039400	1.25392100	0.49855100
C	-1.20251200	1.08845400	-2.23785900
C	-1.32818900	0.50797400	-1.11833000
C	-1.49098200	-0.11419600	0.09518600
H	-0.75179400	-0.88143900	0.34857300
C	-2.14492900	0.55378200	1.22294700
C	-3.05705600	1.60380700	1.03406700
C	-1.92667400	0.08587400	2.52899300
C	-3.74797100	2.14799300	2.11326200
H	-3.22053400	1.99859800	0.02957200
C	-2.61473200	0.63208800	3.60592900
H	-1.21775000	-0.72933800	2.68927800
C	-3.53885200	1.65980800	3.40194100
H	-4.45599600	2.96050300	1.94576300
H	-2.43910300	0.24739500	4.61045000
H	-4.08535300	2.08121800	4.24484300
C	-0.37242400	0.33321000	-5.07560600
C	-2.11670500	2.88495900	-4.49987700
H	-1.25782700	3.53433100	-4.27857200
H	-2.34937800	2.98237300	-5.57086000
H	-2.98030300	3.26992300	-3.93692600
C	-3.36909700	0.11973900	-4.23230400
H	-3.28499100	-0.92955500	-3.91377400
H	-4.17996300	0.59548000	-3.66207700
H	-3.68212400	0.12445600	-5.28674900
C	0.77495100	1.32565200	-5.26278700
H	1.20265900	1.65756800	-4.30094200
H	1.59471300	0.85756900	-5.83604700
H	0.45808800	2.22383600	-5.81572000
C	-0.93910700	-0.02782800	-6.45252800
H	-1.40079200	0.83527900	-6.95793800
H	-0.13054200	-0.39180800	-7.10865400
H	-1.69303400	-0.82646800	-6.39317800
C	0.14930700	-0.93635200	-4.40228500
H	0.63987200	-0.72153700	-3.44050800
H	-0.65459900	-1.66632600	-4.21208600
H	0.89284200	-1.43378300	-5.04859600
Si	-1.77761500	1.10034300	-4.03486400
C	-3.01812700	-1.60603100	-0.50405900
O	-2.96541700	-2.43003100	0.55786800
C	-4.18055300	-0.85830500	-0.76843300
H	-2.37407400	-1.95422500	-1.30859000
C	-3.90141500	-2.33055500	1.55176700
C	-5.15964700	-0.73918200	0.18931800
H	-4.22334900	-0.31437100	-1.70781100
C	-3.70739400	-3.11668200	2.68086100
C	-5.01580700	-1.49105800	1.41923100
O	-6.25506500	-0.01711600	0.06209200
C	-4.63527900	-3.04315500	3.71072400
H	-2.83292900	-3.76234500	2.73941000
C	-5.93540500	-1.42860800	2.47553400
Si	-6.83236900	0.89681900	-1.28668600
C	-5.74626700	-2.19714700	3.61274100
H	-4.49176900	-3.64997200	4.60314500
H	-6.79585600	-0.76922900	2.38279900
C	-7.05354800	-0.27881100	-2.71957500
C	-8.45438400	1.54790900	-0.65688400
C	-5.62666300	2.27793000	-1.65027300
H	-6.46320100	-2.14462600	4.42979000
H	-7.54273200	0.24666400	-3.55297100
H	-6.11213200	-0.69630100	-3.10172700
H	-7.70398600	-1.11778400	-2.43431900
H	-9.14288900	0.72944700	-0.40705500
H	-8.31410800	2.16177000	0.24331200
H	-8.94109300	2.17481500	-1.41711500
H	-4.60694500	1.92850500	-1.86463500
H	-5.97289200	2.83811500	-2.53222300
H	-5.57334400	2.98707400	-0.8119970

TS_{II-IV}

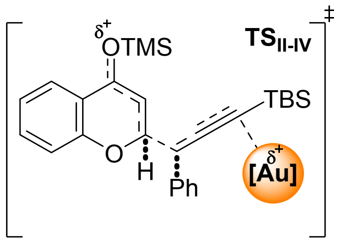

E= -3176.854255

G_{Corr}= 0.755814

1 1

Au	7.20771300	4.51534400	3.50061500
P	7.67954300	5.39089200	1.41243100
O	9.09757500	5.05724300	0.71244200
O	7.62302900	7.00554100	1.34127800
C	10.18158100	4.94557600	1.59381400
C	10.91684300	6.07965700	1.91087800
C	11.98607000	5.95434000	2.79677500
H	12.57534900	6.83299900	3.05445700
C	12.30060800	4.71354600	3.34813000
C	11.55026900	3.58720300	3.01138800
H	11.79973300	2.61613100	3.43689500
C	10.47735400	3.69742800	2.12978500
H	9.87304100	2.83313900	1.85057700
C	6.87903300	7.62107900	2.35889800
C	5.49085100	7.58161500	2.31514500
C	4.77322100	8.14325200	3.37027500
H	3.68395800	8.11115000	3.35712100
C	5.44447300	8.74149500	4.43541800
C	6.83820600	8.78342200	4.44911400
H	7.36423800	9.25047300	5.28074400
C	7.56849400	8.21196900	3.40944100
H	8.65849400	8.21080000	3.41253900
O	6.64206500	4.98012900	0.24206700
C	6.08571400	3.69771200	0.28603300
C	6.72597800	2.65831800	-0.37729900
C	4.89171100	3.50812500	0.97439700
C	6.13857800	1.39395900	-0.36458100
H	7.65800500	2.84811300	-0.90839200
C	4.31884900	2.23752100	0.98296200
H	4.41902200	4.34773200	1.48542100
C	4.93772700	1.18251500	0.31167800
H	6.62304300	0.57221100	-0.89011700
H	3.37784400	2.07256100	1.51442800
H	4.47778300	0.19488500	0.30816200
H	10.65661400	7.03748800	1.46200400
H	13.13636700	4.62267500	4.04012100
H	4.87883200	9.17822600	5.25738300
H	4.98531900	7.11752600	1.46848800
C	6.44404300	3.72888200	5.32671700
C	5.22026000	3.67657800	4.98797900
C	3.91590900	3.61929100	4.58795000
H	3.70007300	2.96952100	3.73245000
C	2.98761800	4.72924500	4.80592600
C	3.18933500	5.67666900	5.82340000
C	1.84473700	4.85043100	3.99961300
C	2.28019400	6.71005800	6.02110400
H	4.07243800	5.59897500	6.46177700
C	0.93812400	5.88777800	4.19646200
H	1.67501000	4.12117800	3.20330600
C	1.14984600	6.82265300	5.20973900
H	2.45441800	7.43496200	6.81629400
H	0.06073300	5.96671400	3.55500000
H	0.43822000	7.63188800	5.36808300
C	8.39080500	5.01474300	7.27732300
C	8.78689400	2.05905700	6.35951900
H	9.33568500	2.31890300	5.44296700
H	9.52248400	1.93128300	7.16780500
H	8.30685600	1.08229800	6.19676900
C	6.41187500	2.75704000	8.19715000
H	5.65011800	3.50252400	8.47141500
H	5.88682600	1.84184500	7.88149200
H	6.98209500	2.50704000	9.10342100
C	9.49453000	5.34340000	6.27047400
H	9.11713600	5.41280100	5.23393400
H	9.95135300	6.31922300	6.51120100
H	10.30019000	4.59343100	6.28103400
C	9.01469700	4.86470100	8.66817000
H	9.72603600	4.02571200	8.72408000
H	9.57282900	5.77841800	8.93387400
H	8.25337800	4.71245600	9.44771500
C	7.37078900	6.15321000	7.30907500
H	6.90943300	6.32293700	6.32371700
H	6.56064900	5.96341300	8.03097100
H	7.86074500	7.09499800	7.61165100
Si	7.52887100	3.37724100	6.81651400
C	3.26762900	1.80728000	5.83535700
O	1.99695200	1.77347400	5.41880200
C	4.19466300	0.83194500	5.42925500
H	3.36960800	2.35573100	6.76860900
C	1.68338000	1.06196100	4.29583900
C	3.90561300	0.03735600	4.34213000
H	5.16470500	0.81447800	5.92191200
C	0.41210800	1.24860800	3.76207100
C	2.60562800	0.17426800	3.72171600
O	4.72403000	-0.84440700	3.80822500
C	0.06114700	0.54159200	2.62199400
H	-0.27111000	1.94718500	4.24205200
C	2.22172800	-0.53603300	2.57226700
Si	6.44678400	-0.96774100	3.89937800
C	0.96390400	-0.35080600	2.02655700
H	-0.92709600	0.68300700	2.18817500
H	2.92852600	-1.23305900	2.12644800
C	6.91243000	-1.52550600	5.61692900
C	6.81558300	-2.26807900	2.62350900
C	7.13612900	0.69551000	3.41141300
H	0.67229300	-0.90131300	1.13428100
H	7.99787100	-1.69449000	5.67335500
H	6.64680700	-0.80869500	6.40583700
H	6.42091600	-2.47941900	5.85511300
H	6.29172800	-3.20869100	2.84132900
H	6.51579400	-1.93475100	1.61964100
H	7.89291900	-2.48439100	2.59493400
H	6.96039300	1.48176900	4.15718400
H	8.22373700	0.61870200	3.25750800
H	6.68753000	1.02627200	2.46020800

2a

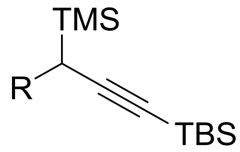

E= -1282.667133

G_{Corr}= 0.365244

0 1

C	4.16015400	2.70792000	4.62672700
C	3.04528400	2.76152200	5.64020700
C	2.60335200	3.97166700	6.18653800
H	3.10469200	4.90084000	5.91048700
C	1.53650900	4.00094900	7.08187000
H	1.21040800	4.95376500	7.49835000
C	0.89061800	2.82121800	7.44721600
H	0.05826500	2.84464000	8.14966300
C	1.32486100	1.60996800	6.91081900
H	0.83432100	0.67889900	7.19402900
C	2.39214800	1.58225600	6.01686400
H	2.73032700	0.62955100	5.60460300
C	5.20693200	3.69215700	4.80496600
C	6.06100200	4.56593600	4.86640000
C	8.94760300	5.28503300	5.48244400
H	9.74231600	6.03321000	5.34791100
H	8.89876300	5.02677500	6.54940900
H	9.24861200	4.38006300	4.93578300
C	7.49851000	6.42806400	3.02697900
H	8.11517900	7.33191600	2.91447200
H	7.98698700	5.62169100	2.46054200
H	6.52295900	6.61876100	2.55652300
C	6.64544600	7.36047400	5.89097300
C	5.42178100	7.97823100	5.21399300
H	4.62235800	7.23842600	5.04833000
H	4.99926500	8.78072400	5.84329700
H	5.67091800	8.42607300	4.23984400
C	7.73514400	8.42465100	6.03517800
H	8.61278200	8.04467100	6.57982400
H	8.08257700	8.80571600	5.06194700
H	7.35081400	9.29011600	6.60220800
C	6.25547600	6.85282900	7.27917300
H	7.10394100	6.38959500	7.80612800
H	5.90136600	7.68962000	7.90617100
H	5.44641900	6.10830000	7.23513200
Si	7.30711700	5.92370000	4.82891300
C	2.75030300	4.67770400	2.67380900
Si	3.48627700	2.95985000	2.82456400
H	5.48206400	1.82746700	1.83059200
H	4.60682300	2.77450700	0.61069300
H	5.66129300	3.59502800	1.78148500
H	2.58780100	0.63148200	2.63969000
C	2.19073000	1.64535700	2.48601500
H	1.30725900	1.76559400	3.12845900
H	1.85216000	1.71186800	1.44156000
C	4.94393400	2.77080300	1.65744800
H	4.59927600	1.69522400	4.62114800
H	2.41908700	4.85405100	1.63977400
H	3.49055800	5.45344700	2.92086300
H	1.87774500	4.81395700	3.32811000

3a´

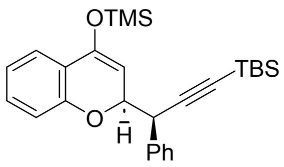

E= -1779.533154

G_{Corr}= 0.487019

0 1

C	1.37216400	1.57602200	-1.84385400
C	1.15108000	0.38525700	-1.69074200
Si	1.74999800	3.37813300	-2.01325400
C	3.46275800	3.54158400	-2.76923600
H	3.69459300	4.59424600	-2.98831200
H	4.24944000	3.15379000	-2.10512100
H	3.52055000	2.98667200	-3.71691100
C	0.47129400	4.14537500	-3.15207000
H	0.57095500	3.73424400	-4.16664700
H	-0.55297700	3.94125300	-2.81147800
H	0.59823200	5.23548600	-3.22207900
C	1.68778500	4.13570000	-0.26597700
C	2.16230300	5.58879400	-0.33249400
H	2.09421800	6.05807700	0.66403400
H	3.21137400	5.66680400	-0.65626600
H	1.55210000	6.19785100	-1.01753500
C	0.25407800	4.09475600	0.26292200
H	-0.42870600	4.70410200	-0.34839900
H	-0.14689800	3.06959500	0.29411200
H	0.21393400	4.49373800	1.29123100
C	2.59967100	3.35500100	0.68058700
H	2.28164200	2.30658400	0.79105300
H	3.64748400	3.35619600	0.34207800
H	2.58450900	3.80914800	1.68642000
C	0.98136600	-1.04262100	-1.44307700
H	-0.03997000	-1.21961400	-1.06699500
C	1.19683300	-1.90594400	-2.66740500
C	2.15203700	-1.58632500	-3.63613100
C	0.47585000	-3.09515900	-2.79968600
C	2.40469900	-2.45473700	-4.69522600
H	2.70369400	-0.64856700	-3.56121400
C	0.72493500	-3.96546000	-3.85778100
H	-0.27774000	-3.34997900	-2.05239600
C	1.69763400	-3.65100700	-4.80494200
H	3.15747200	-2.19373200	-5.43919300
H	0.15950900	-4.89334800	-3.93979800
H	1.89817500	-4.33212900	-5.63133000
C	1.93624200	-1.43458600	-0.26118800
O	1.65293500	-2.77589900	0.13975700
C	3.37829300	-1.19706900	-0.56162200
H	1.62419200	-0.81594200	0.59359000
C	2.41631300	-3.79582600	-0.33353700
C	4.19317000	-2.20680000	-0.91594400
H	3.71309700	-0.16101100	-0.54435700
C	1.90629500	-5.08451600	-0.20786400
C	3.68969600	-3.57996900	-0.88251100
O	5.48124600	-2.07981100	-1.28492400
C	2.65347200	-6.16770100	-0.66132100
H	0.91902700	-5.21803500	0.23342500
C	4.42407900	-4.67992400	-1.33234000
Si	6.34059500	-0.65293600	-1.63368800
C	3.91085800	-5.96939800	-1.23252200
H	2.24614300	-7.17416500	-0.57067800
H	5.41025900	-4.50804100	-1.76021900
C	8.02621900	-1.28944000	-2.11145400
C	5.51038700	0.23519700	-3.05528100
C	6.43331800	0.43503700	-0.11041500
H	4.49172700	-6.81738400	-1.59197300
H	8.68763200	-0.45758100	-2.39317200
H	8.50138000	-1.82714400	-1.27948400
H	7.96680600	-1.97581600	-2.96721700
H	5.41846400	-0.41968600	-3.93381300
H	4.50627600	0.59572000	-2.78955000
H	6.10734400	1.11028900	-3.35338700
H	6.45298100	-0.16592000	0.80993700
H	7.35523900	1.03461100	-0.13309400
H	5.58882000	1.13458500	-0.03688800

3a

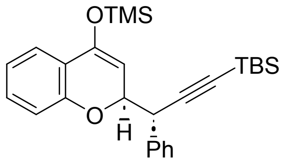

E= -1779.533684

G_{Corr}= 0.486956

0 1

C	1.57731500	1.37023600	-1.68275100
C	1.35072300	0.18026600	-1.53022800
Si	1.93678900	3.16166600	-1.97047200
C	3.63525500	3.28173200	-2.76512000
H	3.89461000	4.32669700	-2.98989900
H	4.42773600	2.86368500	-2.12756900
H	3.64537900	2.72761800	-3.71497500
C	0.63851400	3.82218800	-3.15434000
H	0.70576000	3.29833800	-4.11848300
H	-0.38013200	3.67624500	-2.76978400
H	0.78151000	4.89497100	-3.34921300
C	1.87193900	4.05443900	-0.28963900
C	2.23058500	5.52833400	-0.48925800
H	2.17344500	6.06800300	0.47169700
H	3.25446900	5.65796900	-0.87202200
H	1.54363500	6.03476900	-1.18480500
C	0.46273500	3.95244500	0.29424900
H	-0.28661400	4.44377700	-0.34468000
H	0.14840400	2.90650200	0.43637700
H	0.42145700	4.44421000	1.28150800
C	2.86897300	3.42325200	0.68178200
H	2.64140700	2.36299300	0.87463200
H	3.90531200	3.48620600	0.31363000
H	2.83815000	3.94587000	1.65353500
C	1.10313600	-1.24523200	-1.33294100
H	1.60434100	-1.79281300	-2.15189500
C	-0.37138400	-1.59081500	-1.35444700
C	-1.28868300	-0.86570500	-0.58942000
C	-0.82512400	-2.67129900	-2.11172200
C	-2.63564200	-1.21779200	-0.58010300
H	-0.94589600	-0.01249400	-0.00032500
C	-2.17304900	-3.02492500	-2.10647200
H	-0.11357000	-3.24069300	-2.71259300
C	-3.08176300	-2.29906600	-1.33936200
H	-3.34091300	-0.64306300	0.01948600
H	-2.51344700	-3.86929700	-2.70519300
H	-4.13628400	-2.57265400	-1.33466100
C	1.78401800	-1.71550700	-0.01176800
O	1.59060900	-3.12489900	0.12512300
C	3.23115800	-1.34199500	0.04518600
H	1.23402900	-1.27435100	0.83120700
C	2.42797500	-3.92741600	-0.58417000
C	4.14871800	-2.18314300	-0.46237300
H	3.50342000	-0.36481900	0.43967500
C	1.98543300	-5.20435700	-0.91596100
C	3.72771100	-3.50724700	-0.91865100
O	5.47026400	-1.91174100	-0.55088200
C	2.83415100	-6.06652500	-1.60563500
H	0.97680900	-5.50198400	-0.63098500
C	4.56253800	-4.38722500	-1.61282600
Si	6.09474000	-0.55469200	-1.36736300
C	4.12062800	-5.65948900	-1.96216200
H	2.48365900	-7.06324300	-1.87121900
H	5.57177000	-4.06165500	-1.86468600
C	7.88902800	-0.99610300	-1.61608300
C	5.17318200	-0.38253700	-2.98558300
C	5.90304200	0.98659700	-0.32364500
H	4.77921000	-6.33554700	-2.50512800
H	8.42499400	-0.18945600	-2.13602800
H	8.39432900	-1.16917400	-0.65585800
H	7.98964700	-1.91002300	-2.21862100
H	5.18410900	-1.32472700	-3.55381600
H	4.12354900	-0.09087500	-2.83129800
H	5.64268300	0.38860300	-3.61429100
H	6.21935600	0.81583700	0.71521800
H	6.52024200	1.80285400	-0.72880400
H	4.86004400	1.33391800	-0.31049300
